# Ontogenetic Changes in Feeding Behaviors in Tufted Capuchins

**DOI:** 10.1002/ajpa.70108

**Published:** 2025-08-20

**Authors:** Stephanie L. Canington, Cláudia Misue Kanno, Caitlin B. Yoakum, Mariana Dutra Fogaça, Megan A. Holmes, Claire E. Terhune, José Américo de Oliveira, Janine Chalk‐Wilayto, Myra F. Laird

**Affiliations:** ^1^ Department of Basic and Translational Sciences University of Pennsylvania Philadelphia Pennsylvania USA; ^2^ São Paulo State University (UNESP), School of Dentistry, Research Center “Núcleo de Procriação de Macacos‐Prego” Araçatuba Brazil; ^3^ Department of Anatomy Arkansas Colleges of Health Education Fort Smith Arkansas USA; ^4^ Neotropical Primates Research Group—NeoPReGo São Paulo Brazil; ^5^ Department of Family Medicine and Community Health Duke University School of Medicine Durham North Carolina USA; ^6^ Department of Anthropology University of Arkansas Fayetteville Arkansas USA; ^7^ Department of Biomedical Sciences Mercer University School of Medicine Savannah Georgia USA

**Keywords:** development, feeding behaviors, food properties, *Sapajus*

## Abstract

**Objectives:**

Wild juvenile capuchins exhibit lower feeding success than adults, particularly for mechanically challenging foods, but ontogenetic changes in oral food processing behaviors related to this reduced success are unknown. We test how oral food processing efficiency varies across development in an experimental setting in tufted capuchins (*Sapajus* spp.). Further, we simulate discontinuous feeding observations to test the comparability of behaviors measured in wild and captive settings.

**Materials and Methods:**

Twenty‐nine captive and semi‐wild infants (*n* = 2), juveniles (*n* = 12), older juveniles (*n* = 4), and subadults‐adults (*n* = 11) were video recorded while feeding at the Núcleo de Procriação de Macacos‐Prego Research Center (Araçatuba, Brazil). Each animal was offered a series of five foods ranging in volume, toughness, and elastic modulus.

**Results:**

Measures of oral food processing inconsistently varied with sex; however, younger animals were less efficient in food processing than older individuals. Larger and more mechanically challenging foods were associated with longer feeding sequence durations and an increased frequency of anterior ingestion, posterior ingestion, and chewing during a feeding sequence. Simulated discontinuous data from the first and last halves of the feeding sequences closely replicated continuous results.

**Conclusions:**

Our results indicate younger capuchins have reduced oral food processing efficiency compared to adults through increased duration, behavioral frequencies, number of chews, and behavioral patterns. Further, our continuous and discontinuous comparisons support the use of discontinuous feeding behaviors from the first and last halves of the feeding sequence. We caution that researchers should be careful to capture infrequent behaviors when using discontinuous data.

## Abstract (pt)

**Objectivos:**

Macacos‐prego juvenis de vida livre apresentam menores eficiências alimentares quando comparados a adultos, principalmente quando acessam alimentos mecanicamente desafiadores. No entanto, pouco se sabe sobre os aspectos ontogenéticos do comportamento do processamento oral de alimentos correlacionados a esta eficiência reduzida. Desta forma, realizamos um experimento para testarmos como a eficiência do processamento oral de alimentos varia ao longo da ontogenia de macacos‐prego (*Sapajus* spp.). Além disso, simulamos observações de alimentação descontínuas para testar a comparabilidade dos comportamentos avaliados em animais de cativeiro e de vida livre.

**Materiais e Métodos:**

Vinte e nove animais de cativeiro e de vida livre, dentre eles infantis (*n* = 2), juvenis (*n* = 12), juvenil mais velho (*n* = 4) e subadultos ou adultos (*n* = 11) foram filmados no Núcleo de Procriação de Macacos‐Prego Research Center (Araçatuba, Brazil) durante a alimentação. Cinco tipos de alimentos que variavam quanto ao volume, dureza e módulo de elasticidade foram oferecidos a cada animal.

**Resultados:**

As medidas de processamento oral de alimentos variaram de forma inconsistente quanto aos sexos; contudo, animais mais jovens foram menos eficientes quanto ao processamento de alimentos quando comparados aos mais velhos. Alimentos de maior volume ou dureza foram associados a durações mais longas da sequência de alimentação e a uma frequência aumentada da mastigação, dos processamentos orais anterior e posterior durante a sequência de alimentação. Foram simulados dados descontínuos da primeira e segunda metade das sequencias de alimentação e resultaram em números próximos ao conjunto de dados contínuos.

**Conclusões:**

Nossos resultados indicam que macacos‐prego mais jovens têm uma eficiência menor no processamento oral de alimentos quando comparados a adultos ao apresentarem um aumento na duração, frequência, número de mastigação e padrões comportamentais. Além disso, nossas comparações de dados contínuos e descontínuos dão suporte ao uso de comportamentos alimentares descontínuos da primeira e segunda metades da sequência da alimentação. Nós pontuamos que pesquisadores deveriam ser cuidadosos ao capturar comportamentos infrequentes quando da utilização de dados descontínuos.

## 1 Introduction

Feeding ecology has been the focus of intense study in both captive and wild primates (e.g., Ravosa 1991; Vinyard et al. 2007; Wright et al. 2009; Vogel et al. 2014; Thompson 2017), with a particular emphasis on the mechanics of food items and the feeding system (e.g., Lucas 2004; Ross et al. 2012; Ross and Iriarte‐Diaz 2014; van Casteren et al. 2016). Studies of primate feeding behaviors in the wild capture how an animal interacts with natural foods, surrounding social influences, and the environment. Yet, there are challenges to recording wild feeding behaviors, including observation schedules and visibility of the animal itself (Stevens and Carlson 2008; Zuberbühler and Wittig 2011). Recently, Johnston and Cords (2024) found that variations in observational sampling schedule affected the accuracy of monthly dietary measures in *Cercopithecus mitis stuhlmanni* . While feeding, a wild primate may scan its surroundings for predators or competitors, relocate, or turn away from the observer—resulting in discontinuous and sometimes unusable data, particularly for behaviors that occur infrequently (Stevens and Carlson 2008). In contrast, feeding behaviors recorded in experimental or captive settings are inherently limited in their dietary breadth and lack wild surroundings, but they facilitate continuous visibility and testing specific variables (Stevens and Carlson 2008). Ultimately, studies of primate feeding functional morphology benefit from the integration of laboratory and wild primate data (Dunham et al. 2018, 2019; Stevens and Carlson 2008; Thompson and Vinyard 2019; Wright et al. 2019). Here, we quantify how feeding behaviors vary across ontogeny using experimental foods in a captive sample of tufted capuchins (*Sapajus* spp.) to generate results based on data with continuous (or, *complete*) visibility. We compare these results under “continuous” viewing conditions to non‐continuous viewing conditions. The same hypotheses are tested on a series of simulated discontinuous data (non‐continuous viewing) to assess the influence of data continuity on studies of primate feeding behavior.

A common interface between wild and laboratory feeding experiments is the quantification of food material and geometric properties (FMPs and FGPs). FMPs describe how a material behaves in response to loading and are typically quantified in the primate literature as toughness (*R*; energy required to fracture a material, per unit area) and elastic modulus (*E*; a material's resistance to deformation along the elastic region of a stress–strain curve; Ashby 2002; Lucas 2004). FMPs have been recorded for a range of wild primates, but these data suggest the relationships between FMPs, feeding behavior, and jaw morphology are not straightforward, meaning that primates with robust craniodental morphology do not always consume foods with high toughness and elastic modulus values (Coiner‐Collier et al. 2016). Such is noted among wild mountain gorillas ( *Gorilla beringei* ) with robust morphology who tend to maintain low‐toughness dietary profiles (Glowacka et al. 2017). Food geometric properties (FGPs) capture the size and shape of the food item, and food size is proposed to be a strong influence on feeding behaviors (e.g., Laird et al. 2022; Yamashita 2003). Fewer studies have addressed the role of FGPs in primate feeding behaviors compared to FMPs, though Yamashita et al. (2012) found that food geometry can influence food placement on the tooth row in wild *Lemur catta* , and foods of larger size were chewed more slowly than smaller food items in wild *L. catta* and *Propithecus verreauxi* (Flowers et al. 2023; Yamashita et al. 2024).

Tufted capuchins have feeding system adaptations that facilitate extracting and processing mechanically challenging foods (Daegling 1992; Masterson 1997; Taylor and Vinyard 2009; Wright 2005; Wright et al. 2009). These primates also have well‐documented tool use to extraorally process mechanically challenging and/or embedded foods, where the target tissue is not visible (Chalk‐Wilayto et al. 2022; Gibson 1986). Data from studies of primate behavior in the wild indicate that tufted capuchins begin interacting with mechanically challenging foods early in development (Falótico et al. 2021; Gunst et al. 2010a; Visalberghi et al. 2016), but they have lower success rates and/or increased feeding time compared to adults for the same foods, particularly for embedded foods (Agostini and Visalberghi 2005; Chalk et al. 2016; Gunst et al. 2010b; Truppa et al. 2019). This difference may reflect a juvenile's lack of size and strength needed to perform certain behaviors (Altmann 1980; Boinski and Fragaszy 1989; Ross and Jones 1999). But many primates engage in adult behaviors before obtaining adult size and strength, suggesting that practice and learning play an important role in behavioral development (Musgrave et al. 2021; Nowell and Fletcher 2008; Stone 2006; Watts 1985). Importantly, juvenile tufted capuchin food processing behaviors do not differ from adults for mechanically challenging non‐embedded foods, indicating that practice and learning may be better predictors of food processing compared to size and strength (Chalk et al. 2016; Chalk‐Wilayto et al. 2022).

While feeding behaviors have been well studied in adult and juvenile capuchins in wild environments (e.g., Falótico et al. 2017; Fragaszy and Boinski 1995; Laird et al. 2020b; Panger et al. 2002; Visalberghi et al. 2016), differences in oral food processing efficiency for non‐embedded foods that vary in FMPs and FGPs are not well understood. Chalk‐Wilayto et al. (2022) found mixed results for ontogenetic differences in oral food processing efficiency for non‐embedded foods in wild capuchins. Similarly, sex differences in feeding have primarily been studied in wild environments, with tufted capuchin males having increased efficiency for certain food types and engaging in more strenuous feeding behaviors as a result of larger muscles and body sizes (Agostini and Visalberghi 2005; Fragaszy 1990; Fragaszy and Boinski 1995; Fragaszy et al. 2016). Controlled tests of oral food processing efficiency in a captive environment can elucidate ontogenetic changes in body size and food processing abilities.

**FIGURE 1:**
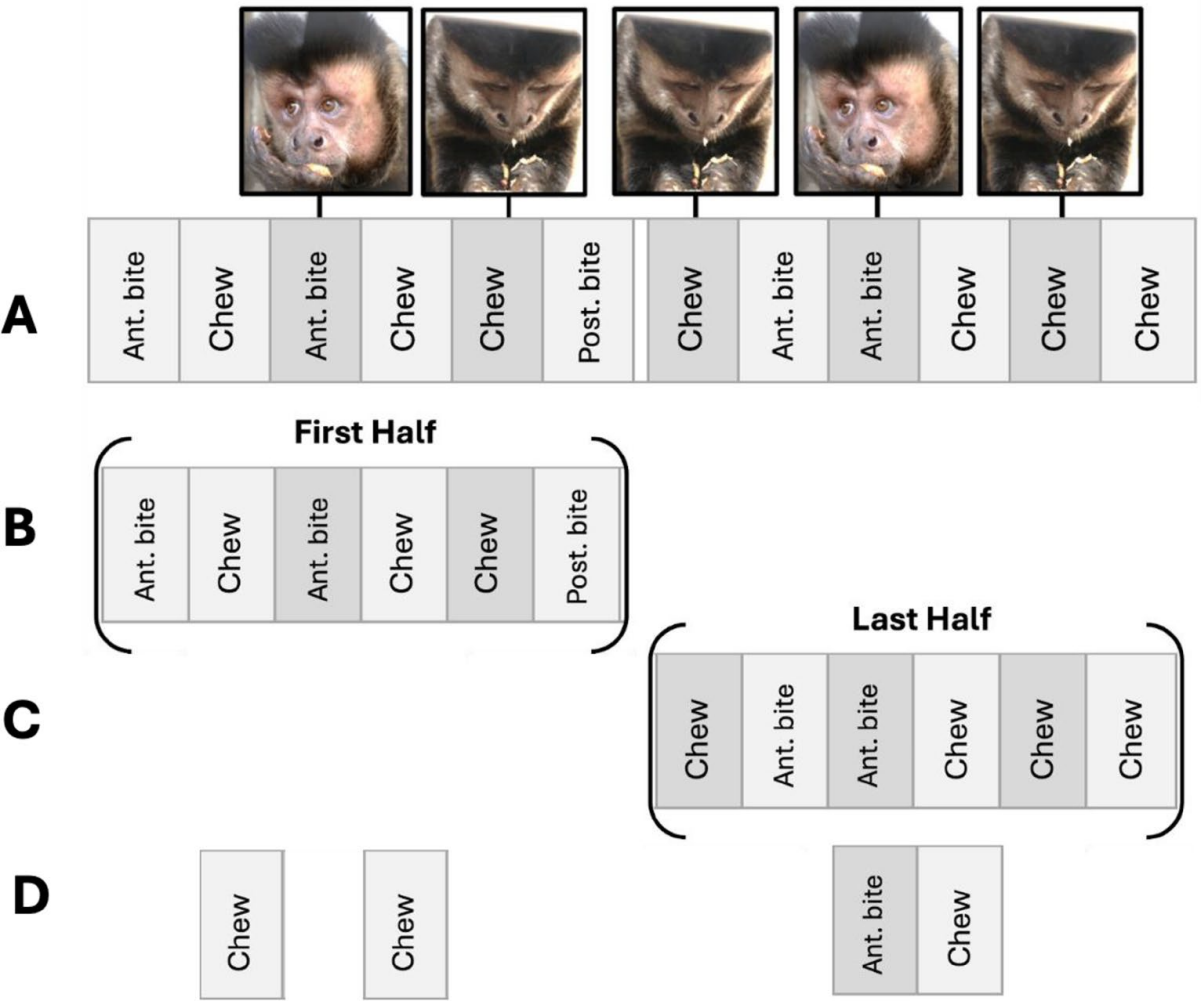
Visualization of a continuous, discontinuous, and random dataset included in this study. (A) The sample continuous dataset encompasses the entirety of the feeding sequence—from food entering the mouth to the final swallow. Discontinuous observations tested in this manuscript are based on the continuous dataset and were defined three ways: (B) as the first half, (C) the last half of the feeding sequence, and (D) as a random sampling of at least four behaviors from the feeding sequence. ant. bite, anterior ingestion; Chew, chewing cycles; post. bite, posterior ingestion.

This manuscript quantifies four behavioral responses at the level of the feeding sequence, defined as ingestion to the final swallow or food discard (Table 1), which operationalizes assessment of oral food processing efficiency: (1) duration of the feeding sequence, (2) behavioral frequency (specifically anterior ingestion frequency, posterior ingestion frequency, and chew frequency), (3) number of chews, and (4) unique behavioral pattern. Because our behavioral data were collected in an experimental setting capturing continuous feeding sequences, we have the opportunity to compare the results of our analyses that use continuous data to the results of the same analyses run using simulated discontinuous feeding behavior data that mimic common interruptions in animal visibility. We conduct all analyses using four versions of our data: continuous, discontinuous behaviors from only the first half of the feeding sequence, discontinuous behaviors from the last half of the feeding sequence, and discontinuous randomly sampled portions of the feeding sequence (SOM Table S1; Figure 1). Our null hypothesis is that sex, age, food volume, and food mechanical properties are not associated with variation in oral food processing efficiency in any of the four datasets. We test a series of alternative hypotheses:

*Male tufted capuchins have increased oral food processing efficiency compared to females of the same age categories*.

**TABLE 1:** Summary of terms used in this paper (modified from Chalk‐Wilayto et al. 2022).

Term	Definition
Feeding sequence	The start of food processing behaviors to the final swallow or discard of the food item.
Feeding sequence duration	Total duration of time (s) of a feeding sequence.
Behavioral frequency	The number of times a food processing behavior (either anterior ingestion, posterior ingestion, and chew) occurs within a feeding sequence.
Number of chews	Total number of chews occurring in a feeding sequence. This differs from chewing behavioral frequency as chewing may occur twice in a feeding sequence but consist of 10 chews.
Unique behavior patterns	Sequential individual behaviors across the feeding sequence were grouped into sets of three, and the number of unique behavioral patterns (sets of three) was summed for each feeding sequence.

Compared to females, larger male tufted capuchins are thought to have increased strength and increased feeding efficiency for certain food types, particularly those that require strenuous extractive behaviors (Jungers and Fleagle 1980; Spagnoletti et al. 2011). Males are expected to process all foods with reduced feeding sequence durations, behavioral frequencies, number of chews, and number of unique behavioral patterns within a feeding sequence compared to females of the same age category. Younger tufted capuchins lack substantial sexual dimorphism, which reduces the expectation of oral food processing efficiency differences in younger age categories. However, foraging differences have been previously noted in juveniles suggesting the possibility of sexually dimorphic oral food processing at young ages (Agostini and Visalberghi 2005).

*Older tufted capuchins have increased oral food processing efficiency compared to younger individuals*.

Compared to adults, immature capuchins in the wild employ oral and extra‐oral behavioral sequences that are less efficient at exploiting challenging food items (Eadie 2015; Gunst et al. 2008). Older individuals are expected to process foods with increased oral efficiency, which we define as reduced feeding sequence duration, reduced behavioral frequencies, fewer chews, and a reduced number of unique behavioral patterns in a feeding sequence compared to younger animals.

*Across individuals, measures of oral food processing efficiency decrease with larger foods (regardless of mechanical properties)*.

As food volume increases, oral food processing efficiency is expected to decrease. This is specifically defined as increased feeding sequence duration, increased behavioral frequency, a greater number of chews, and an increase in the number of unique behavioral patterns within a feeding sequence to account for increased food breakdown and bolus formation. These relationships between food volume and measures of oral food processing efficiency are expected across all individuals, within each individual, between sexes, and within age groups.

*Across individuals, measures of oral food processing efficiency decrease in mechanically challenging foods (regardless of food volume)*.

Mechanically challenging foods are defined as those having higher toughness and elastic modulus values. They are expected to require increased feeding sequence durations, increased behavioral frequencies, a greater number of chews, and an increase in the unique behavioral patterns within a feeding sequence resulting in decreased oral food processing efficiency. Importantly, toughness and elastic modulus represent independent measures of food mechanical properties and will differ in their explanatory power. These relationships between food mechanical properties and measures of oral food processing efficiency are expected across all individuals, within each individual, between sexes, and within age groups.

*For the variables tested in* [Link], [Link], [Link], [Link], *discontinuous data capturing the first half of the feeding sequence are expected to be more similar to the continuous dataset than discontinuous data from the last half of the feeding sequence or data randomly sampled across feeding sequences*.

Electromyography data during initial food breakdown show the greatest amount of variation and are thought to better capture variation in food size and FMPs compared to chews later in the feeding sequence (Vinyard et al. 2008). As a result, we expect that discontinuous data sampling the first half of the feeding sequence will show similar patterns to the variables tested in [Link], [Link], [Link], [Link]. In contrast, data sampled from the last half of the feeding sequence and data sampled randomly across the feeding sequence are not expected to show the same relationships as the continuous data.

## 2 Materials and Methods

### 2.1 Sample

Data were collected from 29 tufted capuchins (*Sapajus* spp.—hybrids of *S. libidinosus* and *S. nigritus* ) during a three‐week experimental period in May–June 2022 at the Núcleo de Procriação de Macacos‐Prego Research Center at the Faculdade de Odontologia‐Campus de Araçatuba‐UNESP, Araçatuba, Brazil. All animals were consistent in their pelage coloration, suggesting similar levels of hybridization. The sample consisted of 26 provisioned semi‐wild individuals that were trapped and released at the end of the project, and three captive adults that are permanently held at the Núcleo for other projects. The provisioned animals receive fruit twice a day, but freely forage around the Núcleo and in nearby vegetation. Measures of oral food processing efficiency were compared between the semi‐wild and captive adults, and there were no significant differences between these groups in any of the measures using the continuous feeding sequences (*p* > 0.05; SOM Table S2). All animals were singly housed during the experiments but were in visual and auditory range of other singly housed individuals. While in captivity, all animals were fed three times a day, and experiments took place between feeding times. All experiments were reviewed and approved by the UNESP‐Araçatuba Ethics Committee on the Use of Animals (00151–2019), the Instituto Chico Mendes de Conservação da Biodiversidade (ICMBio‐77908‐1), the University of Southern California Institutional Animal Care and Use Committee (21294), and the University of Pennsylvania Institutional Animal Care and Use Committee (807394).

Animals were grouped into one of four age classes: (1) infants were defined as young individuals prior to the first permanent molars in occlusion (two total: one female, one male); (2) juveniles—the first permanent molars in occlusion (12 total: seven females, five males); (3) older juveniles—the second permanent molars in occlusion (four total: two females, two males); or (4) an older group containing subadults and adults—with the third permanent molars in occlusion (11 total: three females, eight males; Table 2). For *S. apella* , M3 eruption occurs at approximately 3.25 years of age, but full somatic growth is achieved at least a year later (Galliari 1985; Leigh 1992; Smith et al. 1994); we use the broad category of “subadult/adult” as indicative of M3 occlusion rather than full somatic growth. Sex and dental eruption were observed during the experiment and supplemented with photos and videos of each animal.

**TABLE 2:** Age, sex, body mass, sample origin, and number of foods for the animals in the study.

Animal identification number	Age category^a^	Sex	Body mass (kg)^b^	Captive or semi‐wild	Almonds^c^	Gummy bear^c^	Peanut^c^	Popcorn^c^	Sunflower seed^c^
1	OJ	M	1.75	Semi‐wild	5	2	5	6	7
2	OJ	M	1.6	Semi‐wild	5	2	7	5	3
3	J	M	1.94	Semi‐wild	3	3	5	5	5
4	S‐A	F	2.11	Semi‐wild	5	2	5	5	5
5	J	F	1.25	Semi‐wild	0	2	2	0	2
12	J	M	1.6	Semi‐wild	5	2	4	5	5
14	J	M	1.42	Semi‐wild	5	2	5	5	5
15	J	F	1.2	Semi‐wild	4	2	2	1	0
16	OJ	F	1.9	Semi‐wild	4	2	4	3	4
17	I	M	1.1	Semi‐wild	4	2	5	0	5
18	I	F	1.14	Semi‐wild	3	2	2	1	4
19	J	M	1.57	Semi‐wild	4	2	4	4	5
20	S‐A	F	2.12^b^	Semi‐wild	4	2	4	4	5
21	S‐A	F	2.13	Semi‐wild	4	2	4	2	4
22	S‐A	M	3.5^b^	Semi‐wild	4	2	4	4	5
23	S‐A	M	3.5^b^	Semi‐wild	2	2	4	3	5
24	S‐A	M	3.5^b^	Semi‐wild	5	2	5	5	5
25	S‐A	M	3.5^b^	Semi‐wild	3	2	4	4	2
26	S‐A	M	3.5^b^	Semi‐wild	5	2	5	5	5
27	J	F	1.35	Semi‐wild	3	1	1	1	1
28	J	M	1.93	Semi‐wild	4	0	5	5	5
30	J	F	1.45	Semi‐wild	3	2	4	3	5
31	J	F	1.19	Semi‐wild	5	1	4	5	1
32	J	F	1.54	Semi‐wild	3	2	5	5	4
33	OJ	F	1.9^b^	Semi‐wild	5	3	8	4	3
34	J	F	1.75	Semi‐wild	5	0	0	0	0
95	S‐A	M	3.35	Captive	5	3	5	5	4
98	S‐A	M (Alpha)	3.95	Captive	0	0	0	1	5
99	S‐A	M (Alpha)	3.2	Captive	5	3	5	2	4

^a^ Age: J, Juveniles; I, Infants; S‐A, subadults and adults; OJ, older juveniles.

^b^ Body masses estimated as the average from the respective age‐sex group, e.g., adult females.

^c^ A value of 0 means that the animal chose not to consume any of these items.

### 2.2 Foods

Five foods were individually presented to each animal. No other foods were present in the enclosure at the time of feeding, and most individuals were recorded consuming each food type multiple times (Table 2). Food items that were tested but rejected were excluded from our data. Foods offered to the monkeys included half peanut without a shell, almond without a shell, gummy bear (gelatin‐based candy), popcorn seed (kernel; not included for infants as none were fully consumed), and raw sunflower seed without a shell (Table 3). The number of gummy bears given to each animal was limited because of their high sugar content. These foods were selected because they are consistent in size, have well‐documented food material properties, and have been commonly used in primate feeding experiments (Hylander et al. 2005; Laird et al. 2023; Reed and Ross 2010; Teaford et al. 2017; Williams et al. 2005). Importantly, the foods are resistant to manual deformation as capuchins commonly use their hands to break or pull apart foods (Chalk‐Wilayto et al. 2022). All foods were sourced from a commercial company in the United States (nuts.com). Food volume was measured by water displacement for at least five samples of each food, and food material properties were sourced from the literature, though original data were collected for sunflower seeds following established protocols (Lucas 2004; Table 3). Toughness values were lowest in sunflower seeds 116.12 Jm^−2^ and highest in popcorn kernels 2978.82 Jm^−2^, whereas elastic modulus values were lowest in gummy bears (0.07 MPa) and highest in popcorn kernels 325.4 MPa (Table 3). Food volumes ranged between 0.10 cm^3^ (sunflower seeds) and 1.79 cm^3^ (gummy bears; Table 3).

**TABLE 3:** Measures of size, toughness, and elastic modulus for the experimental foods.

Food item	Volume (cm^3^)	Toughness (Jm^−2^)	Elastic modulus (MPa)	FMP references
Peanut (raw)	0.43 (*n* = 8; SD = 0.12)	255.5 (108.5)	23.9 (6.37)	Agrawal et al. 1997
Sunflower seed (raw)	0.10 (*n* = 18; SD = 0.27)	116.12 (37.32)	4.26 (2.81)	This manuscript^a^
Gummy bear	1.79 (*n* = 8; SD = 0.36)	887.96 (114.15)	0.07 (0.03)	Williams et al. 2005, ^b^
Almond (raw)	1.29 (*n* = 7; SD = 0.43)	308.62 (34.85)	19.42 (7.69)	Williams et al. 2005
Popcorn seed	0.17 (*n* = 8; SD = 0.06)	2978.82 (678.34)	325.4 (218.83)	Williams et al. 2005

^a^ Unlike previously published sunflower seed FMP values (Laird et al. 2016), the seeds used in these experiments were raw, not roasted, which merited new FMP testing. All tests were conducted using an FLS‐II tester (Lucas Scientific). Toughness was tested in six seeds using scissor tests, and elastic modulus was measured using blunt indent tests on five seeds.

^b^ We conducted blunt indent tests on the gummy bear brand used in this study and recorded the same properties as reported in Williams et al. (2005).

### 2.3 Oral Food Processing Efficiency

Individuals were given each food item one at a time, and video was recorded on an iPhone 13 Max and iPhone 11 at 30 frames per second. Videos were viewed frame by frame using the SLEAP graphical user interface (Pereira et al. 2022) by two of the authors, SLC and MFL. Feeding behaviors were coded across the entire feeding sequence, defined from the moment the food enters the oral cavity to the last swallow. Individual behaviors were coded using the ethogram from Chalk‐Wilayto et al. (2022); however, all foods in this study were easily held in the hand and did not involve ‘limb pulls’ that employ the head, neck, or trunk to generate tearing of the food item that have been described elsewhere (e.g., Chalk‐Wilayto et al. 2022; Laird et al. 2020b; Laird et al. 2022). Foods in this study also preclude other types of oral processing such as tongue‐palatal processing (processing food against the hard palate by the tongue), which may be used for soft, easily deformable foods (e.g., Hiiemae et al. 1996). Thus, all behaviors were classified as anterior bites, posterior bites, or chew. Anterior bites were located on the incisors and canines, and posterior bites occurred on the premolars and molars. Frame numbers for the start and end of each behavior were recorded and converted to seconds. Inter‐ and intraobserver errors were tested by scoring two gummy bear trials on three separate instances each spaced approximately 1 month apart. One‐way ANOVAs were used to test for differences in bite and chew numbers between scoring instances, and differences between scorers were not statistically significant (Intraobserver: MFL: *p* = 0.469; SLC: *p* = 0.131; Interobserver: *p* = 0.178). Oral food processing efficiency was defined in four ways (Table 1) and means for each measure of oral food processing efficiency are provided for the continuous dataset (SOM Table S3).

### 2.4 Data Continuity

Analyses for each hypothesis were conducted using four different sets of data to compare results for continuous and discontinuous data. All analyses were first conducted on the 488 total continuous feeding sequences. These continuous feeding sequences were used to create three types of *discontinuous sequences*: first half of the complete feeding sequence (488 total), the last half of the complete feeding sequence (488 total), and random behavior samples (488 × 10,000 total; Figure 1; see Johnston and Cords 2024 for a similar approach). The randomly sampled discontinuous data were generated using custom R code to subset at least four behaviors from each of the continuous 488 feeding sequences. Each iteration resulted in 488 new feeding sequences for which each of the hypotheses were retested. The random sampling and hypothesis testing were repeated a total of 10,000 times. An example continuous (complete) feeding sequence may read: *anterior bite*, *chew*, *posterior bite*, *chew*, *anterior bite*, *anterior bite*, *chew*. The discontinuous first half sequence would be *anterior bite*, *chew*, *posterior bite*, *chew*, and the discontinuous last half sequence would be *anterior bite*, *anterior bite*, *chew*. One of the 10,000 randomly sampled sequences might be: *chew*, *posterior bite*, *chew*, *anterior bite*, *anterior bite*.

### 2.5 Analyses

All analyses were conducted in R (R core team 2023). Hypotheses were tested using linear mixed models (LME) in the R packages ‘nlme’ (Pinheiro et al. 2023) and ‘emmeans’ (Lenth 2016) for pairwise comparisons. Post hoc comparisons included Tukey's adjustment to minimize type one error. Models were run individually with each measure of oral processing efficiency as each model's response variable (SOM Table S1). Explanatory variables varied with the hypothesis being tested: H1 = age*sex, H2 = age, H3 = age*food volume, and H4 = age*toughness and age*elastic modulus, and food type and animal identification were included as random factors. Food type and animal identification had a significant effect on the explanatory models in each hypothesis. For age group contrasts, we focus on juvenile‐older juvenile, juvenile‐subadult/adult, and older juvenile‐subadult/adult comparisons as the infant sample consists of two individuals, but all contrasts are reported in the supplemental online material. In LME models for [Link], [Link], [Link], the addition of sex as a random variable did not improve the models and was not included. All hypotheses were tested across all food types and within each individual food. For analyses within an individual food type, only animal identification was included as a random variable. These models were repeated for the continuous data as well as for each of the discontinuous datasets. Significance was set at 0.05.

## 3 Results

### 3.1 Hypothesis 1‐Sex Differences

Continuous feeding sequences (Table 4): Across all individuals (combined food types and ages), feeding sequence duration, chew number, and unique behavioral patterns did not differ between males and females (SOM Tables S4–S6). However, there were significant interactions between age and sex for duration, with subadult/adult males having shorter feeding durations compared to older juvenile males (*p* = 0.04) and juvenile females (*p* = 0.004). No other measure of oral feeding efficiency exhibited significant interactions between age and sex.

**TABLE 4:** Significant results for Hypothesis 1–variation in oral food processing behavior with sex.

Dataset	Measure of feeding behavior	Age and sex contrasts	*p* ^a^
Continuous	Duration	S‐A male/J female	0.0041
		S‐A male/OJ male	0.0359
	Chew number	S‐A male/J female	0.0209
		S‐A male/OJ male	0.0379
	Chew frequency	S‐A male/J female	0.0041
	Anterior ingestion frequency	S‐A male/J female	0.0120
	Unique behavioral patterns	S‐A male/J female	< 0.0001
		S‐A male/J male	0.0006
First half	Duration	S‐A male/J female	0.001
		S‐A male/OJ male	0.001
		S‐A male/J male	0.029
	Chew number	S‐A male/J female	0.0140
		S‐A male/OJ male	0.0108
	Anterior ingestion frequency	S‐A female/J female	0.0491
		S‐A male/J female	0.0065
	Unique behavioral patterns	S‐A male/J female	0.0001
		S‐A male/J male	0.0293
Last half	Duration	S‐A male/J female	0.0155
	Chew frequency	S‐A female/J female	0.0002
		S‐A male/J female	< 0.0001
	Anterior ingestion frequency	S‐A male/J female	0.0120
	Unique behavioral patterns	S‐A male/J female	0.0016
		S‐A male/J male	0.0036

*Note:* Non‐significant results are available in SOM Table S4.

^a^ Significant results only (*α* = 0.05).

Discontinuous feeding sequences (Table 4): Analyses examining variation in oral processing efficiency with sex using data from the first half of the feeding sequences were the same as the continuous dataset; though the interaction between age and sex was non‐significant (but see pairwise contrasts). Pairwise contrasts for duration in the last half of the feeding sequence indicated that subadult/adult males had significantly shorter feeding durations compared to juvenile females.

Of the 10,000 randomly sampled datasets across all individuals, significant interactions between age and sex were found for 3.16% of the feeding duration tests, 0% of the anterior ingestion frequency, 0.08% of posterior ingestion frequency, 0% of chew frequency, 0.57% of the chew number tests, and 1.17% of the unique behavioral pattern tests, which are similar to results from the continuous data (Table 4; SOM Table S7).

**FIGURE 2:**
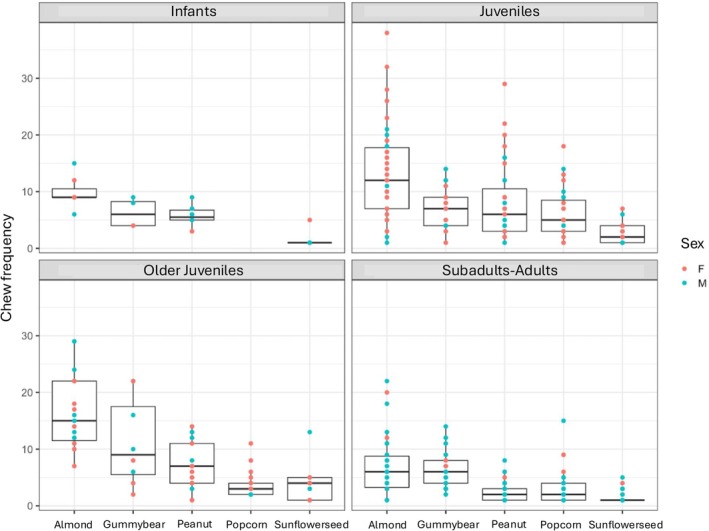
Chew frequency (a type of behavioral frequency) broken across age classes, sex, and food types.

**FIGURE 3:**
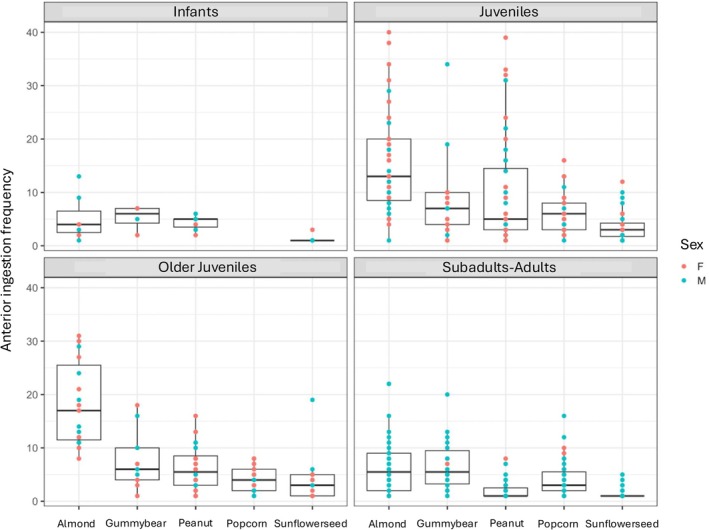
Anterior ingestion frequency (a type of behavioral frequency) broken across age classes, sex, and food types.

**FIGURE 4:**
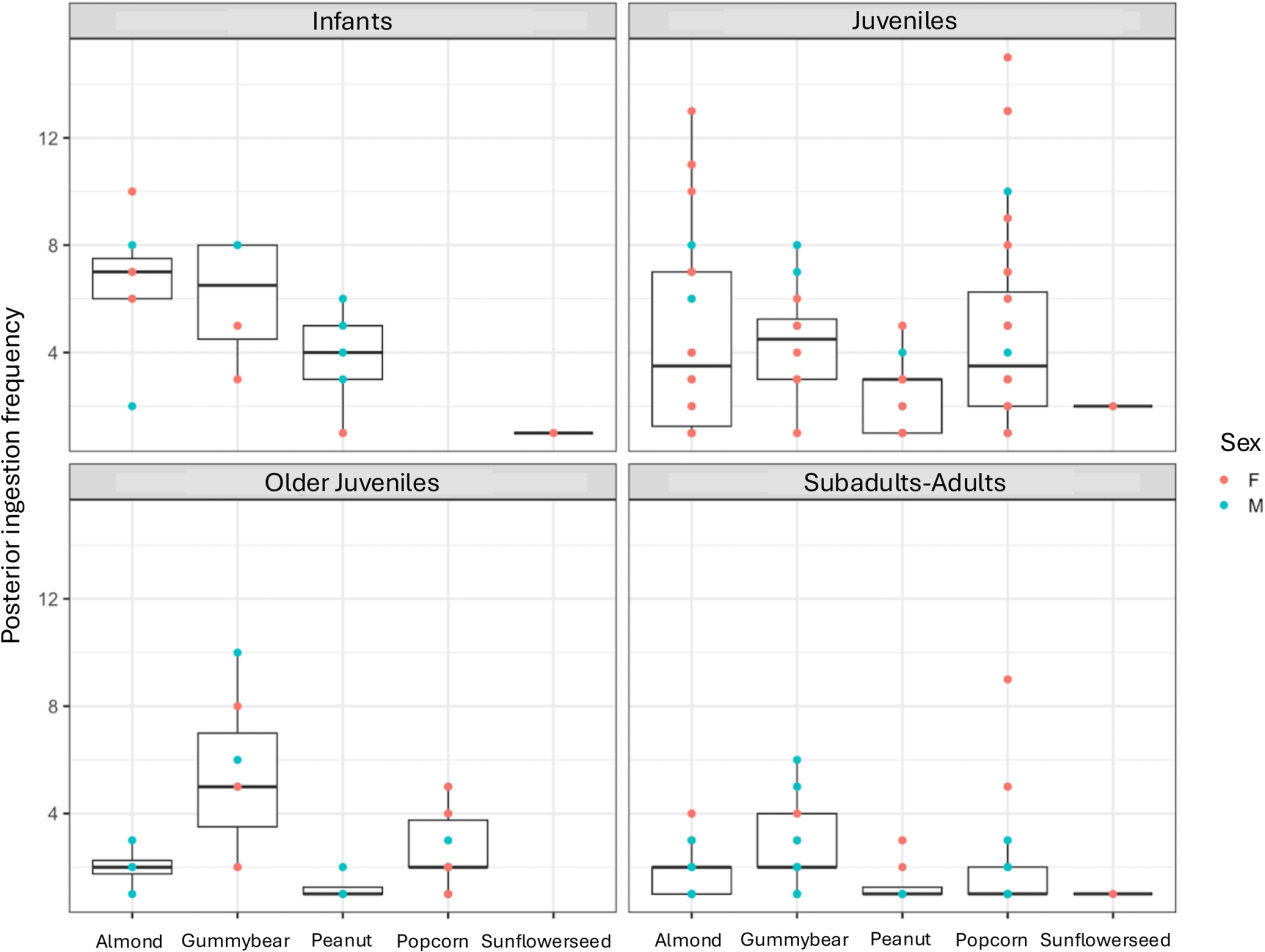
Posterior ingestion frequency (a type of behavioral frequency) broken across age classes, sex, and food types.

Within‐food types: Within each of the five food types, chew number, behavioral frequency, and unique behavioral patterns did not significantly vary by sex for the continuous data (*p* > 0.05; SOM Table S8; Figures 2, 3, 4). Sex was a significant variable for feeding sequence duration for popcorn kernels (older juveniles; *p* = 0.024) and sunflower seeds (juveniles; *p* = 0.048)–the smallest foods in this study. For both, males had lower feeding sequence durations than females.

### 3.2 Hypothesis 2‐Variation by Age

**FIGURE 5:**
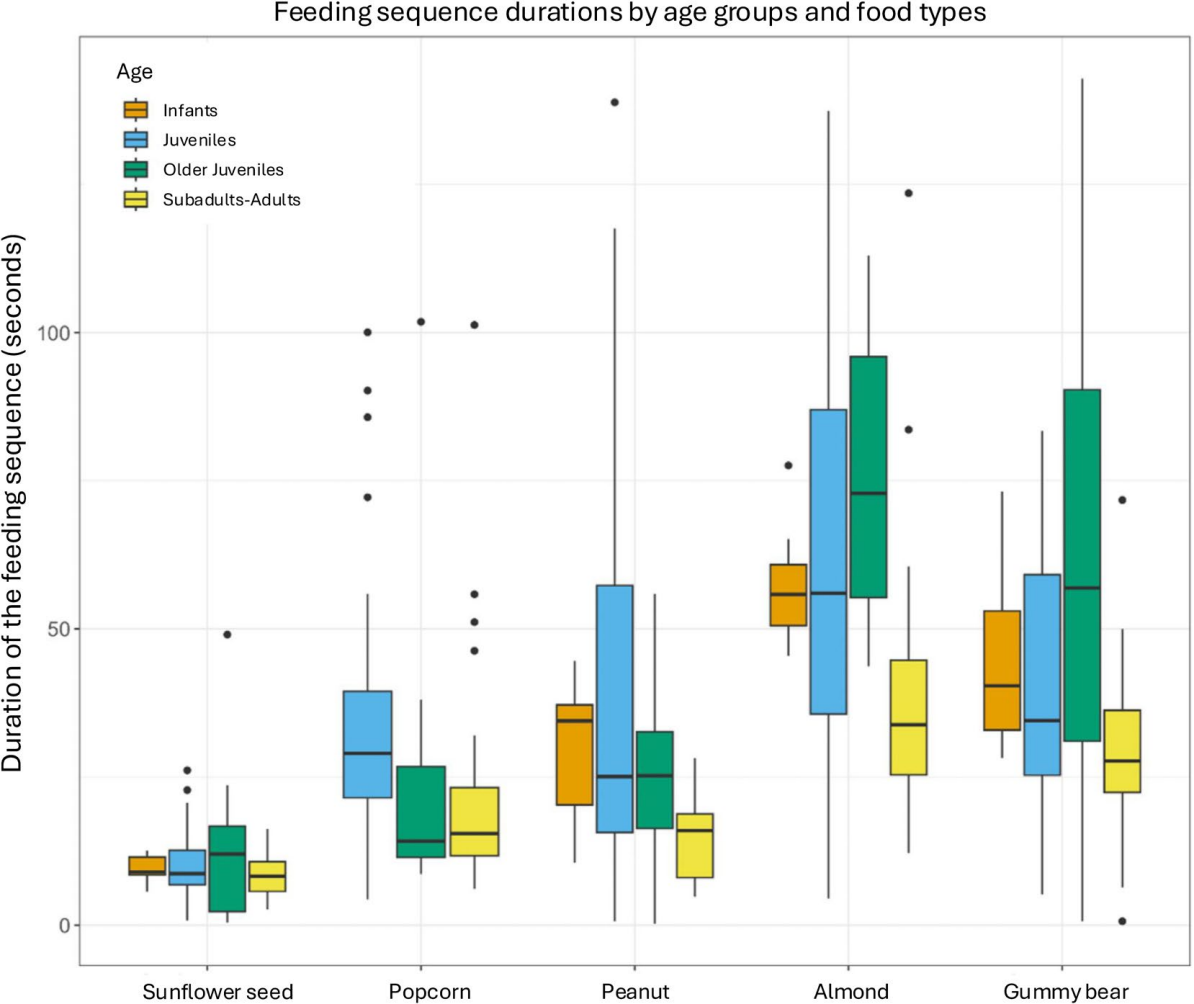
Feeding sequence durations grouped by age and food type. Across all food types, subadults/adults had significantly shorter feeding sequence durations compared to younger age groups (*p* = 0.014); however, within‐food‐type analyses were non‐significant (*p* > 0.05). Infants did not consume popcorn seeds. Food types are organized in ascending order of volume, and larger foods were associated with significantly longer feeding sequences (*p* < 0.01).

**FIGURE 6:**
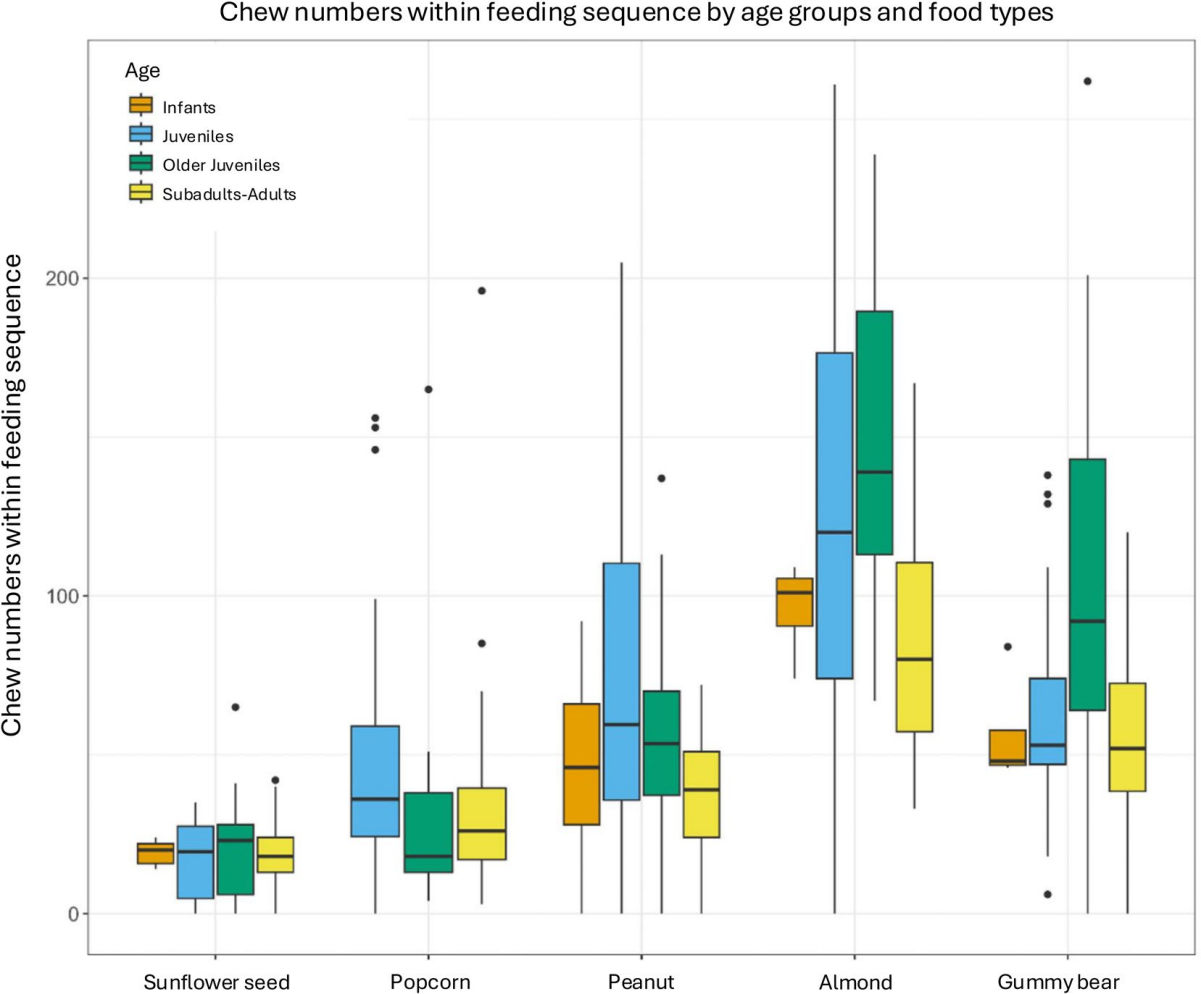
Chew numbers within feeding sequence durations by age groups and food types. Across age groups, mean chew numbers ranged from 16.9–21.8 (sunflower seed), 29.8–45.5 (popcorn), 38.2–78.7 (peanut), 83.5–149 (almond), and 55.3–114 (gummy bear). Across food types, subadults/adults and juveniles (*p* = 0.020) and subadults/adults and older juveniles (*p* = 0.012) differed significantly in chew numbers. Infants did not consume popcorn seeds. Food types are organized in ascending order of volume.

**FIGURE 7:**
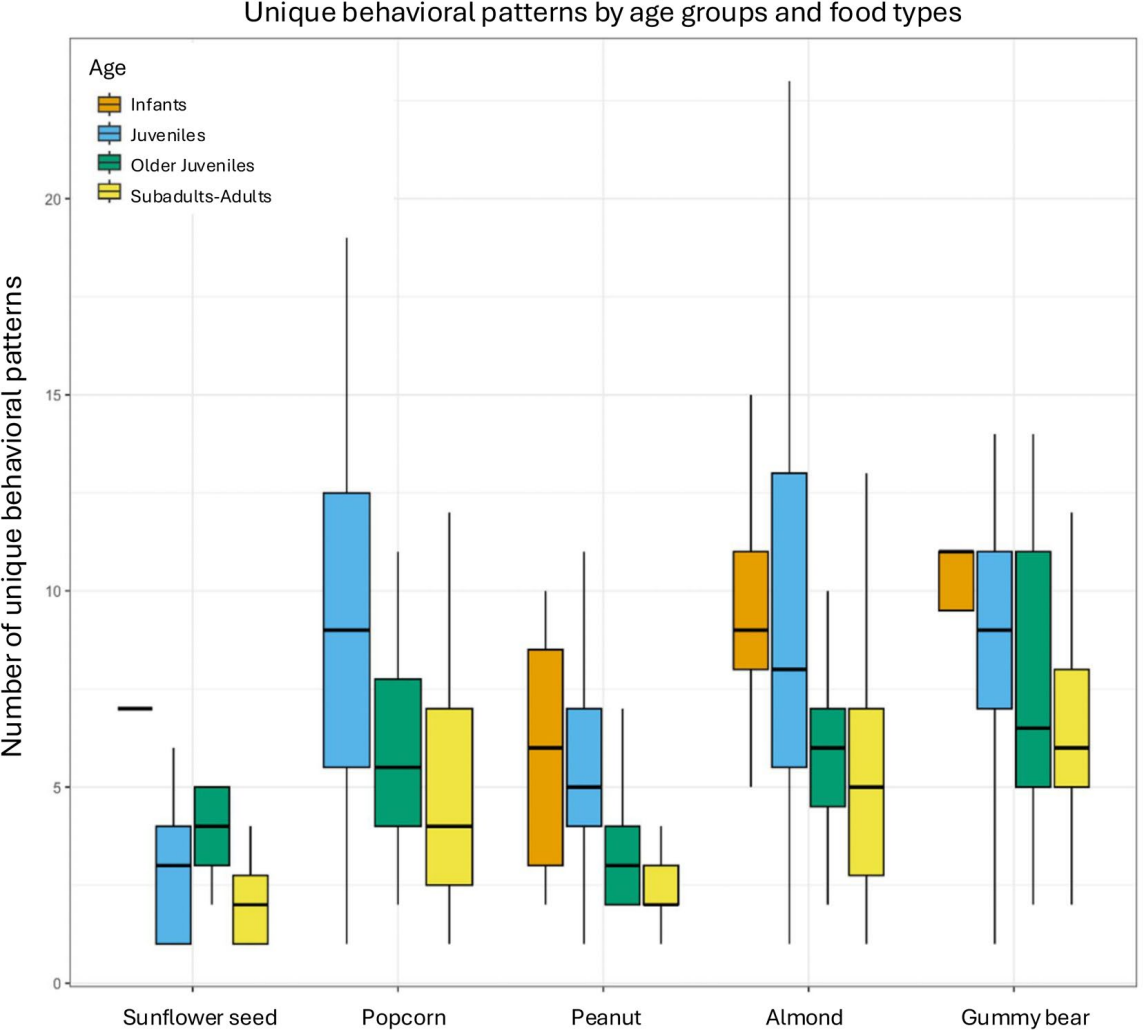
Number of unique behavioral patterns within a feeding sequence by age groups and food types. Across age groups, the number of unique behavioral patterns was significant for almonds (*p* = 0.027), peanuts (*p* = 0.036), and sunflower seeds (*p* = 0.017). Infants did not consume popcorn seeds. Food types are organized in ascending order of volume.

Continuous feeding sequences: Across all individuals (combined food types), feeding sequence duration (Figure 5), chew number (Figure 6), number of unique behavioral sequences (Figure 7), and the three measures of behavioral frequencies (Figures 2, 3, 4) all significantly varied (all *p* < or = 0.01; SOM Table S9). Compared to younger age classes, subadult/adult individuals had shorter feeding sequence durations, lower chew numbers, reduced number of unique behavioral patterns (SOM Table S3), and lower frequencies of anterior ingestion, posterior ingestion, and chewing (Figures 2, 3, 4; SOM Table S5). Pairwise contrasts for each age group suggest feeding sequence durations, anterior ingestion frequency, and posterior ingestion frequency were significantly shorter in the subadults/adults compared to juveniles. Chew number and chew frequency were significantly lower in subadult/adult individuals compared to juveniles and older juveniles (SOM Table S3; Figures 2, 3, 4). The number of unique behavioral patterns was significantly smaller in subadult/adult individuals compared to infants and juveniles and between older juveniles and juveniles. Older juveniles also had fewer unique behavioral patterns compared to juveniles.

Discontinuous feeding sequences: Analyses examining variation in age using data from the first half of the feeding sequences replicated the continuous data with one exception; chewing frequencies from the first half of the feeding sequence did not significantly vary with age (*p* = 0.30; SOM Table S9). Contrasts were similar to the continuous data, with subadults/adults having significantly shorter durations, lower anterior and posterior frequencies, and fewer behavioral patterns compared to juveniles (SOM Table S9). Analyses from the last half of the feeding sequences showed two primary differences from the continuous feeding sequence results; anterior and posterior ingestion frequencies did not significantly vary with age (*p* = 0.55 and 0.07; SOM Table S9). Contrasts for the other measures of oral feeding efficiency again found significant differences between the subadult/adult and juvenile groups in duration, chewing frequency, chew number, and behavioral pattern from the last half of the feeding sequence.

Analyses of age‐group differences in oral processing efficiency using 10,000 randomly sampled feeding sequence datasets indicated 38.8% of tests for duration, 77.91% of tests for anterior ingestion frequency, 74.58% of tests for posterior ingestion frequency, 53.42% of tests for chew frequency, 43.16% of tests for chew number, and 99.94% of tests for unique behavioral pattern showed significant differences between age groups (SOM Table S10).

Within‐food types: Chew number, behavioral frequency, and unique behavioral patterns varied by age group for some food types (SOM Table S8; Figures 2, 3, 4). Almonds, peanuts, and sunflower seeds had the greatest number of significant differences between age groups. Duration did not vary by age group within any of the food types.

### 3.3 Hypothesis 3‐Variation by Food Volume

Continuous feeding sequences (Table 5): Across all individuals and age groups, there was a significant interaction between age and food volume for all measures of oral food processing efficiency except behavioral pattern (*p* = 0.55) meaning that foods with larger volumes were associated with longer feeding sequence durations, higher frequency of chews, more chew numbers, increased anterior ingestion, and increased posterior ingestion (Figures 2, 3, 4; all *p* < 0.05; SOM Table S11).

**TABLE 5:** Results for Hypotheses 3 and 4–variation in oral food processing behavior with FGPs and FMPs.

Continuous dataset: measure of feeding behavior	Age group contrasts	Volume (*p*)^a^	Toughness (*p*)^a^	Elastic modulus (*p*)^a^
Duration	S‐A/J	0.0411	0.0259	0.0209
Anterior ingestion frequency	S‐A/J	0.0007	—	0.0014
	S‐A/OJ	0.0240	—	NS
Posterior ingestion frequency	S‐A/J	NS	—	0.0014
Chew frequency	S‐A/J	0.0013	—	0.0011
	S‐A/OJ	0.0042	—	NS
Chew number	S‐A/J	0.0315	0.0332	0.0316
Unique behavioral patterns	S‐A/J	0.0030	0.0017	0.0047

*Note:* Non‐significant results are available in SOM Tables S11 and S13.

^a^ Significant results only (*α* = 0.05); “‐” denotes where the model failed to converge; “NS”, not significant for that variable.

Discontinuous feeding sequences: With a few exceptions, most measures of oral food processing from the first and last halves of the feeding sequence were not correlated with food volume. Chew number and anterior ingestion frequency from the first half of the feeding sequence had significant interactions between age and food volume (*p* values < 0.01; SOM Table S11). From the last half of the feeding sequence, duration, chewing frequency, and number of chews had significant interactions between age and food volume (*p* values < 0.02; SOM Table S11). Contrasts between subadults/adults and juveniles yielded the largest number of significant differences (SOM Table S11).

Analyses of relationships between measures of oral processing efficiency and food volume using 10,000 randomly sampled feeding sequence datasets show that 16.13% of analyses of feeding sequence durations, 30.08% of anterior ingestion frequency, 23.08% of posterior ingestion frequency, 49.14% of chewing frequency, 48.32% of chew numbers, and 0% of behavioral patterns had significant interactions between age and food volume (SOM Table S12).

### 3.4 Hypothesis 4‐Variation by FMPs

Continuous feeding sequences (Table 5): Across all individuals and all age groups, none of the measures of food processing efficiency had a significant interaction between age and food toughness; although models for chewing, anterior ingestion, and posterior ingestion frequencies failed to converge (SOM Table S13). There were significant interactions between age and elastic modulus for chewing frequency (*p* = 0.04) and chew number (*p* = 0.03) with the subadult/adult group having reduced chewing frequency and fewer chew numbers compared to the juvenile group for stiffer food items.

Discontinuous feeding sequences: Data from the first and last halves of the feeding sequence mostly corresponded with the continuous data results. There was a significant interaction between food toughness and age for duration and between age and elastic modulus for chew numbers in the first half of the feeding sequence, and between elastic modulus and age for chewing frequency in the last half of the feeding sequence (SOM Table S13). Subadults/adults had significantly shorter durations than infants and older juveniles and less frequent chews than juveniles (SOM Table S13).

Analyses of the interaction between age and food toughness using 10,000 randomly sampled feeding sequence datasets indicated that 8.3% of tests for duration, 0.01% of tests for chew number, and 0.13% of tests for behavioral pattern were significant for toughness; all toughness models for behavioral frequencies failed to converge (SOM Table S14). A total of 31.42% of tests for duration, 0% for anterior ingestive frequency, 12.87% for posterior ingestive frequency, 18.43% of chewing frequency, 55.62% of chew number, and 1.57% of unique behavioral pattern had significant interactions for age and elastic modulus.

### 3.5 Hypothesis 5‐Differences Between Continuous and Discontinuous Datasets

Overall, the discontinuous data replicated 90.48% of the continuous results. The first half of each feeding sequence replicated 90.51% of the results; the last half of the feeding sequence replicated 96.45% of the results, and the randomly sampled data replicated 84.75% of the results (SOM Table S15). The first and last halves and random sampling of the feeding sequence perfectly replicated all oral food processing efficiency continuous data for H1. Discontinuous data replicated 84.13% of the H2 results, 91.2% of the H3 results, and 89.57% of the H4 results.

## 4 Discussion

Experimental approaches to the primate feeding system have informed how foods are processed through jaw muscle activation patterns, kinematics, and bone strain (e.g., Hylander 1984; Hylander et al. 1998; Iriarte‐Díaz et al. 2011; Laird et al. 2020a; Laird et al. 2023; Vinyard et al. 2008). Studies of primate feeding ecology have similarly amassed detailed data on food processing and use of the feeding system in a variety of wild environments (e.g., Norconk et al. 2009; Ungar 1994; Yamashita et al. 2012), but few studies have bridged the divide between experimental and ecological approaches (but see Ross et al. 2016; Stevens and Carlson 2008; Thompson and Vinyard 2019; Williams et al. 2005; Wright et al. 2019). We examined ontogenetic changes in oral food processing efficiency in an experimental setting using continuous data and repeated the analyses using simulated discontinuous variation akin to data collection from primates in the wild. By comparing the results of these analyses, we discuss the role of data continuity in understanding the development of oral food processing efficiency and for studies of feeding behaviors in wild primates.

### 4.1 Few Differences in Oral Food Processing Efficiency Between Males and Females

In our continuous dataset, we found few differences in oral food processing efficiency between males and females (H1) with most of the sex differences in duration between subadults/adults and older juveniles and juveniles. In wild capuchins, sex differences have been related to body size and strength for skilled behaviors such as nut cracking (e.g., Spagnoletti et al. 2011) and food competition (e.g., Fragaszy et al. 2016). However, the foods in this study did not require strength for processing, and the animals were individually housed, although still visible and audible with others, lessening the impact of food competition. Thiery and Sha (2020) noted higher ingestion rates and more frequent use of the molars during biting in a captive group of female capuchins, and our results are consistent with these findings. We also find that, with the exception of duration for popcorn (Juveniles; SOM Table S8), sex differences are not present in younger animals, indicating that feeding behaviors are being modified up to adulthood in conjunction with developmental variables such as size and/or strength.

Developmental studies of feeding time in wild capuchins indicate that juveniles have reduced efficiency in food processing (Chalk‐Wilayto et al. 2022; Janson and van Schaik 1993; Truppa et al. 2019), despite juveniles and adults consuming wild foods with similar FMPs (Chalk et al. 2016). While the infant sample in this study was limited to two animals, we find preliminary support for reduced food processing efficiency in younger captive capuchins feeding on less mechanically challenging foods (H2). Our preliminary results indicate that younger capuchins, particularly those in the juvenile age group, had longer feeding sequence durations, increased behavioral frequencies, a larger number of chews, and an increased number of unique behavioral patterns indicating reduced oral food efficiency compared to subadult/adult individuals. The two infants spent almost twice the amount of time (duration) consuming a gummy bear compared to the subadult/adult. This result indicates that capuchin oral food processing efficiency essentially doubles between infants and subadults/adults, allowing the latter to consume greater volumes of food in order to meet the metabolic needs of a larger body size (Ross et al. 2009). When coupled with the sex results, this indicates that the size of the oral cavity and its strength, as indicated by body mass, are strong indicators of food processing efficiency.

### 4.2 Tougher and Larger Foods Have Reduced Oral Food Processing Efficiency

The experimental foods in this study were chosen to produce relatively straightforward feeding behaviors, as they are all small, easily held in the hand, and require little to no extraoral processing. As such, these foods differ from many items consumed by wild capuchins (Chalk‐Wilayto et al. 2016; Laird et al. 2020b; Wright 2005), but they allow us to conduct controlled tests of FMPs and food size. Our experimental foods were novel to these study animals–indicating that reduced oral feeding efficiency in younger capuchins is independent of food familiarity. Our experimental foods also did not include concealed food items, defined as foods where the target tissue is not visible, for example, a nut in a shell (Gibson 1986). This allowed us to test food processing efficiency specific to the oral cavity, rather than food processing inclusive of extractive behaviors or a combination of oral and extraoral extractive behaviors such as stripping of an inedible exocarp. Chalk‐Wilayto et al. (2022) found that embedded foods were processed with reduced efficiency in younger wild tufted capuchins and emphasized the role of sensorimotor development in order to access these foods. Our results suggest differences in oral processing efficiency with age result from both the oral cavity itself and during extraoral food preparation behaviors, as shown by Chalk‐Wilayto et al. (2022). While we extrapolate from experimental foods to those found in the wild using FMPs and FGPs, we emphasize that embedded status and the combination of oral and extraoral food processing behaviors are important factors in wild capuchin feeding.

Foods with larger volumes (H3) were associated with longer feeding sequences, higher behavioral frequencies, and more chews, but not unique behavioral patterns. Foods with higher elastic modulus values were associated with higher chewing frequencies and numbers of chews, but toughness was not related to any measure of oral food processing efficiency, although frequency was not tested. We note that the toughest experimental food in our study was popcorn seeds at 2978.82 Jm^−2^, which is substantially below previously reported wild tufted capuchin FMPs (Wright 2005; Chalk et al. 2016; Laird et al. 2020b). Despite the smaller range of FMPs, mechanically challenging and larger foods increased the length of engagement with the food item in most age groups, but not how they were being processed (behavioral pattern). This indicates that foods were processed using a relatively common behavioral pattern that was lengthened or shortened in response to FMPs and FGPs.

Among the tested food properties, food volume had a greater number of significant relationships with measures of oral food processing efficiency compared to FMPs. These results add to the results of previous studies in wild capuchins, ring‐tailed lemurs, and sifakas, suggesting that aspects of food geometry, such as size or shape, may be relatively more informative for variation in feeding behaviors than FMPs (Chalk‐Wilayto et al. 2022; Flowers et al. 2023; Laird et al. 2022; Yamashita et al. 2024). Larger foods have greater surface area when broken down in the mouth, presumably resulting in greater stimulation of mucosal, tongue, and palatal sensory receptors. However, the food with the largest volume in our sample, gummy bears, yielded neither the most chews for processing nor the longest feeding sequence duration. In this case, we note that there are other relevant food and feeding variables not addressed within this study, including the many aspects of food texture (e.g., adhesiveness, springiness, or gumminess) and taste (e.g., sugar content) that likely influence feeding behavior (de Lavergne et al. 2017; Foegeding et al. 2011, 2015; Koç et al. 2013; Szczesniak 2002). Gummy bears, with their high sugar content, were consumed more rapidly than the other experimental foods despite their large volume. Future work incorporating more complex aspects of sensory inputs would add a further and dynamic component to the study of intra‐oral food breakdown and may provide insight into differences between measures of oral food processing behaviors.

### 4.3 Discontinuous Feeding Data Can Yield Robust Relationships

Experimental settings allow complete feeding sequences to be recorded from start to finish, which differs from the often‐interrupted observations of primates in the wild (Stevens and Carlson 2008; Johnston and Cords 2024). Although discontinuous feeding sequences were created methodologically in this study, we directly tested how our results would change if using discontinuous data. We found that measures of oral food processing efficiency gleaned from only the first half and last half of the feeding sequence successfully approximate results from our continuous dataset. This similarity was somewhat unexpected for the last half of the feeding sequence as the beginning of the feeding sequence has been linked to initial food breakdown, food size, and FMPs compared to later in the feeding sequence that focuses on bolus formation (Lucas 2004; Vinyard et al. 2008). However, the experimental foods in this study were small and did not require extraoral processing. This resulted in a narrower range of feeding behaviors than those noted in wild capuchin feeding studies (e.g., Falótico et al. 2021; Falótico 2023; Chalk‐Wilayto et al. 2022; Laird et al. 2020b), and oral food processing behaviors in the last half of the feeding sequence did not substantially differ from the first half. Our results for each hypothesis using randomly selected portions of a feeding sequence did not replicate the results as well as the first and last halves of the continuous data, and we do not advocate using this sampling approach. Based on this comparison, we suggest that discontinuous data recorded in the wild can accurately capture feeding behaviors, but we caution against using data that randomly capture portions of the feeding sequence and discontinuous data that do not capture the breadth of an animal's behavior.

## 5 Conclusions

Primate feeding behaviors have a long history of being studied in the laboratory and in the field, with both settings providing important information for the relationships between behavior, morphology, and ecology. This study found that measures of oral food processing efficiency varied across ontogeny for experimental foods and that food properties influence feeding duration and behavioral frequency, particularly food volume. Additionally, we demonstrate that discontinuous datasets from the first and last halves of the feeding sequence, which are common in behavioral data collection in the wild, successfully approximate continuous datasets. We caution that results from discontinuous datasets are highly dependent on the food's geometric and material properties and that discontinuous data may be misleading if it does not capture the full range of behaviors.

## Author Contributions

**Stephanie L. Canington:** conceptualization (equal), data curation (equal), formal analysis (equal), methodology (equal), resources (equal), software (equal), validation (equal), visualization (equal), writing – original draft (equal), writing – review and editing (equal). **Cláudia Misue Kanno:** investigation (equal), writing – review and editing (equal). **Caitlin B. Yoakum:** investigation (equal), writing – review and editing (equal). **Mariana Dutra Fogaça:** writing – review and editing (equal). **Megan A. Holmes:** funding acquisition (equal), writing – review and editing (equal). **Claire E. Terhune:** funding acquisition (equal), writing – review and editing (equal). **José Américo de Oliveira:** project administration (equal), supervision (equal), writing – review and editing (equal). **Janine Chalk‐Wilayto:** funding acquisition (equal), writing – review and editing (equal). **Myra F. Laird:** conceptualization (equal), data curation (equal), formal analysis (equal), funding acquisition (equal), investigation (equal), methodology (equal), project administration (equal), resources (equal), software (equal), supervision (equal), validation (equal), visualization (equal), writing – original draft (equal), writing – review and editing (equal).

## Ethics Statement

All experiments were reviewed and approved by the UNESP‐Araçatuba Ethics Committee on the Use of Animals (00151‐2019), the Instituto Chico Mendes de Conservação da Biodiversidade (ICMBio‐77908‐1), the University of Southern California Institutional Animal Care and Use Committee (21294), and the University of Pennsylvania Institutional Animal Care and Use Committee (807394).

## Conflicts of Interest

The authors declare no conflicts of interest.

## Supporting information


**Data S1:** Supporting Information

## Data Availability

The data that support the findings of this study are available upon reasonable request.
