# Supplementary material for: Ontogenetic Changes in Feeding Behaviors in Tufted Capuchins
Source: Am J Biol Anthropol. 2025 Aug 20;187(4):e70108. doi: 10.1002/ajpa.70108 (PMC12368475; doi:10.1002/ajpa.70108)
Supplement: Supplementary file 1 — Data S1: Supporting Information [file AJPA-187-e70108-s001.pdf]

SOM Tables:

Table	Legend	Page(s)
Table 1.	Organization of the data within each hypothesis.	2 - 6
Table 2.	Results of LME models testing differences in each measure of feeding behavior between the captive individuals and wild adults using complete feeding sequences.	7
Table 3.	Feeding sequence duration and chew number summaries (continuous dataset).	8 - 9
Table 4.	Results of LME models testing differences in oral feeding efficiency with age:sex (H1).	10 - 35
Table 5.	Behavior types by food type and capuchin age and sex (continuous dataset).	36 - 43
Table 6.	Behavioral frequencies by food type and capuchin age category and sex (continuous dataset).	44 - 48
Table 7.	Results of LME models testing differences between age and sex groups and measures of feeding behavior using 10,000 randomly sampled feeding sequences (H5).	49 - 57
Table 8.	Measures of feeding behaviors within each food type (by sex and age).	58
Table 9.	Results of LME models testing differences in age (H2).	59 - 66
Table 10.	Results LME models testing differences in age and measures of feeding behavior in of 10,000 randomly sampled feeding sequences. (H5)	67 - 68
Table 11.	Results of LME models testing differences in each measure of feeding behavior varies with food volume (H3).	69 - 77
Table 12.	Results LME models testing differences in food volume and measures of feeding behavior in of 10,000 randomly sampled feeding sequences. (H5 - volume).	78 - 80
Table 13.	Results of LME models testing differences in each measure of feeding behavior varies with food toughness and elastic modulus (FMPs; H4).	81 - 94
Table 14.	Results LME models testing differences in food toughness and elastic modulus and measures of feeding behavior in of 10,000 randomly sampled feeding sequences (H4 - FMP).	95 - 98
Table 15.	Comparison of results for each hypothesis using the continuous and discontinuous datasets (NS = not significant, S = significant). The tally column compares how many of the discontinuous results match the results from the continuous dataset. The random tests were considered "significant" if over 50% of the 10,000 samples were significant.	99 - 105

SOM Table 1. Organization of the data within each hypothesis.

Hypothesis number	Explanatory variable	Response variable-Definition of oral processing efficiency	Dataset
H1	Sex	Duration	Complete feeding sequences
			First half of the feeding sequences
			Last half of the feeding sequences
			Random
		Behavioral frequencies (Anterior ingestion, Posterior ingestion, & Chew)	Complete feeding sequences
			First half of the feeding sequences
			Last half of the feeding sequences
			Random
		Number of chews	Complete feeding sequences
			First half of the feeding sequences
			Last half of the feeding sequences
			Random
		Behavioral pattern	Complete feeding sequences

			First half of the feeding sequences
			Last half of the feeding sequences
			Random
H2	Age	Duration	Complete feeding sequences
			First half of the feeding sequences
			Last half of the feeding sequences
			Random
		Behavioral frequency (Anterior ingestion, Posterior ingestion, & Chew)	Complete feeding sequences
			First half of the feeding sequences
			Last half of the feeding sequences
			Random
		Number of chews	Complete feeding sequences
			First half of the feeding sequences
			Last half of the feeding sequences
			Random

		Behavioral pattern	Complete feeding sequences
			First half of the feeding sequences
			Last half of the feeding sequences
			Random
H3	Food volume	Duration	Complete feeding sequences
			First half of the feeding sequences
			Last half of the feeding sequences
			Random
		Behavioral frequency (Anterior ingestion, Posterior ingestion, & Chew)	Complete feeding sequences
			First half of the feeding sequences
			Last half of the feeding sequences
			Random
		Number of chews	Complete feeding sequences
			First half of the feeding sequences

			Last half of the feeding sequences
			Random
		Behavioral pattern	Complete feeding sequences
			First half of the feeding sequences
			Last half of the feeding sequences
			Random
H4	FMPs- toughness or elastic modulus	Duration	Complete feeding sequences
			First half of the feeding sequences
			Last half of the feeding sequences
			Random
		Behavioral frequency (Anterior ingestion, Posterior ingestion, & Chew)	Complete feeding sequences
			First half of the feeding sequences
			Last half of the feeding sequences
			Random

	Number of chews	Complete feeding sequences
		First half of the feeding sequences
		Last half of the feeding sequences
		Random
	Behavioral pattern	Complete feeding sequences
		First half of the feeding sequences
		Last half of the feeding sequences
		Random

SOM Table 2. Results of LME models testing differences in each measure of feeding behavior between the captive individuals and wild adults using complete feeding sequences.

**Feeding sequence duration**	numDF	denDF	F-value	p-value
(Intercept)	1	146	131.7203	<.0001
Captive vs Wild	1	46	2.66718	0.1093
**Feeding sequence behavioral frequency**	numDF	denDF	F-value	p-value
(Intercept)	1	408	16.04875	0.0001
Captive vs Wild	1	46	0.725874	0.3986
**Feeding sequence number of chews**	numDF	denDF	F-value	p-value
(Intercept)	1	146	17.44889	0.0001
Captive vs Wild	1	46	3.908989	0.054
**Number of unique behavioral patterns within a feeding sequence**	numDF	denDF	F-value	p-value
(Intercept)	1	89	29.66885	<.0001
Captive vs Wild	1	38	0.95385	0.3349

SOM Table 3. Feeding sequence duration and chew number summaries (continuous dataset).

Food Type[1]	Age Class[2]	Mean ln Duration (SD)	Mean Chew Numbers (SD)	Mean Chew Frequency (SD)	Mean Anterior Ingestion Frequency (SD)	Mean Posterior Ingestion Frequency (SD)	Mean Unique Behavioral Pattern (SE)
Sunflower seed	S-A	2.0319 (0.474)	18.8 (9.73)	1.38 (0.90)	1.47 (1.00)	1 (NA)	1.92 (0.649)
	OJ	1.9961 (1.331)	21.8 (17.4)	3.92 (3.17)	3.82 (4.33)	NA	3.72 (0.773)
	J	2.0614 (0.784)	16.9 (11.9)	2.45 (1.61)	3.58 (2.71)	2 (NA)	3.04 (0.600)
	I	2.2137 (0.293)	19.2 (4.12)	1.67 (1.63)	1.33 (0.82)	1 (NA)	7.00 (1.527)
Popcorn	S-A	2.8373 (0.592)	33.8 (31.8)	3.08 (2.80)	4.26 (3.31)	1.96 (1.80)	5.40 (1.31)
	OJ	2.9008 (0.637)	29.8 (36.7)	3.78 (2.46)	4.18 (2.30)	2.56 (1.42)	6.15 (1.75)
	J	3.2794 (0.788)	45.5 (39.8)	5.94 (4.27)	6.43 (4.02)	4.81 (3.72)	9.21 (1.32)
	I	NA	NA	NA	NA	NA	NA
Peanut	S-A	2.6102 (0.490)	38.2 (17.1)	2.24 (1.65)	2.07 (1.68)	1.38 (0.74)	3.11 (1.05)
	OJ	2.7397 (1.376)	53.8 (34.8)	7 (3.95)	6.33 (4.26)	1.25 (0.5)	3.73 (1.21)
	J	3.2568 (1.121)	78.7 (58)	8.03 (6.60)	9.85 (10.15)	2.45 (1.26)	6.00 (0.88)
	I	3.2404 (0.615)	46.6 (31.1)	5.83 (2.04)	4.29 (1.38)	3.8 (1.92)	5.14 (1.67)
Almond	S-A	3.4776 (0.507)	83.5 (34.1)	6.86 (4.97)	6.67 (5.74)	1.86 (0.91)	5.29 (1.22)

	OJ	4.265 (0.322)	149 (51.1)	16.53 (6.49)	18.32 (7.92)	2 (0.82)	6.03 (1.64)
	J	3.9133 (0.800)	123 (71.8)	13.4 (8.37)	15.19 (9.51)	4.53 (3.48)	9.23 (1.18)
	I	4.0337 (0.189)	96.6 (12.9)	9.86 (2.85)	5.14 (4.30)	6.57 (2.44)	9.58 (2.27)
Gummy bear	S-A	3.1040 (0.973)	55.3 (29.4)	6.29 (3.24)	6.73 (4.62)	2.82 (1.63)	6.55 (1.38)
	OJ	3.6085 (1.604)	114 (80.8)	11.25 (7.85)	7.78 (5.83)	5.43 (2.94)	7.88 (1.88)
	J	3.4672 (0.702)	65.1 (39.9)	7.12 (3.60)	8.47 (7.91)	4.38 (2.16)	8.62 (1.40)
	I	3.7521 (0.414)	56.5 (18.4)	6.25 (2.63)	5.25 (2.36)	6 (2.45)	9.50 (2.49)

[1]Food Types
[2]Age: S-A = Subadults and adults; OJ = Older Juveniles; J = Juveniles; I = Infants

SOM Table 4. Results of LME models testing differences in oral feeding efficiency with age:sex (H1).

**Dataset**	**Measure of feeding behavior**	**All individuals**	**numDF**	**denDF**	**F-value**	**p-value**	
Complete feeding sequences	Duration	(Intercept)	1	357	108.5231 1	<.0001	
Complete	Duration	Age	3	119	3.95737	0.0099	
Complete	Duration	Sex	1	119	0.96376	0.3282	
Complete	Duration	Age:Sex	3	119	3.63527	0.0149	
**Complete feeding sequences**	**Duration**	**contrast**	**estimate**	**SE**	**df**	**t.ratio**	**p-value**
Complete	Duration	S-A f — I f	0.023524	0.360	119	0.065	1.0000
Complete	Duration	S-A f — OJ f	0.253854	0.230	119	1.105	0.9545
Complete	Duration	S-A f — J f	-0.249045	0.188	119	-1.323	0.8882
Complete	Duration	S-A f — S-A m	0.337808	0.173	119	1.951	0.5193
Complete	Duration	S-A f — I m	-0.386696	0.317	119	-1.221	0.9243
Complete	Duration	S-A f — OJ m	-0.292231	0.227	119	-1.290	0.9009
Complete	Duration	S-A f — J m	-0.000765	0.184	119	-0.004	1.0000
Complete	Duration	I f — OJ f	0.230329	0.373	119	0.617	0.9986
Complete	Duration	I f — J f	-0.272570	0.349	119	-0.781	0.9938
Complete	Duration	I f — S-A m	0.314284	0.342	119	0.919	0.9837
Complete	Duration	I f — I m	-0.410220	0.431	119	-0.951	0.9801
Complete	Duration	I f — OJ m	-0.315755	0.372	119	-0.849	0.9897
Complete	Duration	I f — J m	-0.024289	0.348	119	-0.070	1.0000
Complete	Duration	OJ f — J f	-0.502899	0.213	119	-2.358	0.2720
Complete	Duration	OJ f — S-A m	0.083955	0.200	119	0.419	0.9999
Complete	Duration	OJ f — I m	-0.640550	0.332	119	-1.927	0.5354

Complete	Duration	OJ f — OJ m	-0.546084	0.248	119	-2.203	0.3575
Complete	Duration	OJ f — J m	-0.254618	0.210	119	-1.212	0.9270
Complete	Duration	J f — S-A m	0.586854	0.151	119	3.883	0.0041
Complete	Duration	J f — I m	-0.137651	0.305	119	-0.451	0.9998
Complete	Duration	J f — OJ m	-0.043186	0.210	119	-0.206	1.0000
Complete	Duration	J f — J m	0.248281	0.164	119	1.515	0.7978
Complete	Duration	S-A m — I m	-0.724504	0.296	119	-2.445	0.2300
Complete	Duration	S-A m — OJ m	-0.630039	0.197	119	-3.203	0.0359
Complete	Duration	S-A m — J m	-0.338573	0.146	119	-2.316	0.2937
Complete	Duration	I m — OJ m	0.094465	0.330	119	0.286	1.0000
Complete	Duration	I m — J m	0.385931	0.303	119	1.273	0.9070
Complete	Duration	OJ m — J m	0.291466	0.207	119	1.411	0.8506
**Dataset**	**Measure of feeding behavior**	**All individuals**	**numDF**	**denDF**	**F-value**	**p-value**	
Complete feeding sequences	Chew frequency	(Intercept)	1	439	60.36827	5.62E-14	
Complete	Chew frequency	Age	3	21	6.47632	0.002826	
Complete	Chew frequency	Sex	1	21	3.008164	0.097503	
Complete	Chew frequency	Age:Sex	3	21	0.840651	0.486836	
**Complete feeding sequences**	**Chew frequency**	**contrast**	**estimate**	**SE**	**df**	**t.ratio**	**p-value**
Complete	Chew frequency	S-A f — I f	-2.67095	3.554883	21	-0.75135	0.993971
Complete	Chew frequency	S-A f — OJ f	-4.79193	2.541954	21	-1.88514	0.574519
Complete	Chew frequency	S-A f — J f	-6.46674	1.960286	21	-3.29888	0.056032
Complete	Chew frequency	S-A f — S-A m	0.32313	1.880547	21	0.171828	1
Complete	Chew frequency	S-A f — I m	-1.91202	3.229454	21	-0.59206	0.99862

Complete	Chew frequency	S-A f — OJ m	-4.40378	2.495885	21	-1.76442	0.648506
Complete	Chew frequency	S-A f — J m	-2.54824	2.016023	21	-1.26399	0.902073
Complete	Chew frequency	I f — OJ f	-2.12098	3.742951	21	-0.56666	0.998957
Complete	Chew frequency	I f — J f	-3.7958	3.375037	21	-1.12467	0.943845
Complete	Chew frequency	I f — S-A m	2.994077	3.329356	21	0.899296	0.98286
Complete	Chew frequency	I f — I m	0.758929	4.239991	21	0.178993	1
Complete	Chew frequency	I f — OJ m	-1.73283	3.711818	21	-0.46684	0.999704
Complete	Chew frequency	I f — J m	0.122709	3.407712	21	0.036009	1
Complete	Chew frequency	OJ f — J f	-1.67482	2.283683	21	-0.73338	0.994788
Complete	Chew frequency	OJ f — S-A m	5.115057	2.215614	21	2.308641	0.334197
Complete	Chew frequency	OJ f — I m	2.879909	3.435384	21	0.838308	0.98851
Complete	Chew frequency	OJ f — OJ m	0.388149	2.75716	21	0.140779	1
Complete	Chew frequency	OJ f — J m	2.24369	2.331703	21	0.962254	0.975087
Complete	Chew frequency	J f — S-A m	6.789873	1.51332	21	4.486741	0.004157
Complete	Chew frequency	J f — I m	4.554725	3.030355	21	1.503034	0.797563
Complete	Chew frequency	J f — OJ m	2.062965	2.23229	21	0.924147	0.980045
Complete	Chew frequency	J f — J m	3.918506	1.678698	21	2.334253	0.321723
Complete	Chew frequency	S-A m — I m	-2.23515	2.979394	21	-0.7502	0.994026
Complete	Chew frequency	S-A m — OJ m	-4.72691	2.162604	21	-2.18575	0.397976
Complete	Chew frequency	S-A m — J m	-2.87137	1.584855	21	-1.81175	0.619588
Complete	Chew frequency	I m — OJ m	-2.49176	3.401437	21	-0.73256	0.994823
Complete	Chew frequency	I m — J m	-0.63622	3.066705	21	-0.20746	0.999999
Complete	Chew frequency	OJ m — J m	1.855541	2.281392	21	0.813337	0.990361
**Dataset**	**Measure of feeding behavior**	**All individuals**	**numDF**	**denDF**	**F-value**	**p-value**	
Complete feeding	Chew number	(Intercept)	1	357	14.815870	0.0001	

sequences							
Complete	Chew number	Age	3	119	4.727119	0.0038	
Complete	Chew number	Sex	1	119	0.514756	0.4745	
Complete	Chew number	Age:Sex	3	119	1.800148	0.1509	
**Complete feeding sequences**	**Chew number**	**contrast**	**estimate**	**SE**	**df**	**t.ratio**	**p-value**
Complete	Chew number	S-A f — I f	11.807	19.89	119	0.594	0.9989
Complete	Chew number	S-A f — OJ f	-15.240	13.19	119	-1.155	0.9426
Complete	Chew number	S-A f — J f	-25.561	10.70	119	-2.388	0.2567
Complete	Chew number	S-A f — S-A m	3.411	9.93	119	0.343	1.0000
Complete	Chew number	S-A f — I m	-7.339	18.22	119	-0.403	0.9999
Complete	Chew number	S-A f — OJ m	-32.850	13.08	119	-2.511	0.2010
Complete	Chew number	S-A f — J m	-7.748	10.61	119	-0.731	0.9959
Complete	Chew number	I f — OJ f	-27.047	20.72	119	-1.306	0.8951
Complete	Chew number	I f — J f	-37.368	19.21	119	-1.946	0.5228
Complete	Chew number	I f — S-A m	-8.396	18.82	119	-0.446	0.9998
Complete	Chew number	I f — I m	-19.146	24.15	119	-0.793	0.9932
Complete	Chew number	I f — OJ m	-44.657	20.66	119	-2.162	0.3823
Complete	Chew number	I f — J m	-19.554	19.19	119	-1.019	0.9707
Complete	Chew number	OJ f — J f	-10.321	12.17	119	-0.848	0.9898
Complete	Chew number	OJ f — S-A m	18.651	11.51	119	1.620	0.7371
Complete	Chew number	OJ f — I m	7.900	19.12	119	0.413	0.9999
Complete	Chew number	OJ f — OJ m	-17.610	14.32	119	-1.230	0.9213
Complete	Chew number	OJ f — J m	7.492	12.10	119	0.619	0.9986
Complete	Chew number	J f — S-A m	28.972	8.55	119	3.387	0.0209
Complete	Chew number	J f — I m	18.222	17.49	119	1.042	0.9669

Complete	Chew number	J f — OJ m	-7.289	12.06	119	-0.604	0.9988
Complete	Chew number	J f — J m	17.814	9.33	119	1.910	0.5469
Complete	Chew number	S-A m — I m	-10.750	17.05	119	-0.631	0.9984
Complete	Chew number	S-A m — OJ m	-36.261	11.38	119	-3.185	0.0379
Complete	Chew number	S-A m — J m	-11.159	8.42	119	-1.325	0.8877
Complete	Chew number	I m — OJ m	-25.510	19.06	119	-1.339	0.8821
Complete	Chew number	I m — J m	-0.408	17.45	119	-0.023	1.0000
Complete	Chew number	OJ m — J m	25.102	11.98	119	2.096	0.4236
**Dataset**	**Measure of feeding behavior**	**All individuals**	**numDF**	**denDF**	**F-value**	**p-value**	
Complete feeding sequences	Ant. frequency	(Intercept)	1	454	48.11229	1.39E-11	
Complete	Ant. frequency	Age	3	21	6.112832	0.003718	
Complete	Ant. frequency	Sex	1	21	1.15307	0.295092	
Complete	Ant. frequency	Age:Sex	3	21	0.763217	0.527332	
**Complete feeding sequences**	**Ant. ingestion frequency**	**contrast**	**estimate**	**SE**	**df**	**t.ratio**	**p-value**
Complete	Ant. frequency	S-A f — I f	-0.07075	4.294906	21	-0.01647	1
Complete	Ant. frequency	S-A f — OJ f	-5.1166	3.127665	21	-1.63592	0.724852
Complete	Ant. frequency	S-A f — J f	-8.08315	2.427331	21	-3.33006	0.052552
Complete	Ant. frequency	S-A f — S-A m	-0.58295	2.336528	21	-0.24949	0.999996
Complete	Ant. frequency	S-A f — I m	-0.75825	4.008959	21	-0.18914	0.999999
Complete	Ant. frequency	S-A f — OJ m	-4.67021	3.105728	21	-1.50374	0.7972
Complete	Ant. frequency	S-A f — J m	-4.29584	2.501602	21	-1.71724	0.677003
Complete	Ant. frequency	I f — OJ f	-5.04585	4.512873	21	-1.1181	0.945458
Complete	Ant. frequency	I f — J f	-8.01241	4.059023	21	-1.97397	0.520331

Complete	Ant. frequency	I f — S-A m	-0.5122	4.005383	21	-0.12788	1
Complete	Ant. frequency	I f — I m	-0.6875	5.162895	21	-0.13316	1
Complete	Ant. frequency	I f — OJ m	-4.59946	4.497697	21	-1.02263	0.965493
Complete	Ant. frequency	I f — J m	-4.22509	4.10387	21	-1.02954	0.96425
Complete	Ant. frequency	OJ f — J f	-2.96656	2.794948	21	-1.0614	0.95811
Complete	Ant. frequency	OJ f — S-A m	4.533649	2.716461	21	1.668954	0.705648
Complete	Ant. frequency	OJ f — I m	4.358349	4.241645	21	1.027514	0.964617
Complete	Ant. frequency	OJ f — OJ m	0.446389	3.400786	21	0.131261	1
Complete	Ant. frequency	OJ f — J m	0.820758	2.859687	21	0.28701	0.999989
Complete	Ant. frequency	J f — S-A m	7.500205	1.867836	21	4.015451	0.012002
Complete	Ant. frequency	J f — I m	7.324905	3.755157	21	1.950626	0.534482
Complete	Ant. frequency	J f — OJ m	3.412945	2.770378	21	1.231942	0.912975
Complete	Ant. frequency	J f — J m	3.787314	2.070618	21	1.829074	0.608961
Complete	Ant. frequency	S-A m — I m	-0.1753	3.697111	21	-0.04742	1
Complete	Ant. frequency	S-A m — OJ m	-4.08726	2.691174	21	-1.51876	0.789405
Complete	Ant. frequency	S-A m — J m	-3.71289	1.963386	21	-1.89106	0.57088
Complete	Ant. frequency	I m — OJ m	-3.91196	4.225495	21	-0.9258	0.979847
Complete	Ant. frequency	I m — J m	-3.53759	3.803588	21	-0.93007	0.979327
Complete	Ant. frequency	OJ m — J m	0.374369	2.835678	21	0.132021	1
**Dataset**	**Measure of feeding behavior**	**All individuals**	**numDF**	**denDF**	**F-value**	**p-value**	
Complete feeding sequences	Post. ingestion frequency	(Intercept)	1	202	41.75116	7.60E-10	
Complete	Post. frequency	Age	3	21	3.764495	0.026258	
Complete	Post. frequency	Sex	1	21	0.402037	0.532886	
Complete	Post. frequency	Age:Sex	3	21	0.309317	0.818387	

Complete feeding sequences	Post. ingestion frequency	contrast	estimate	SE	df	t.ratio	p-value
Complete	Post. frequency	S-A f — I f	-1.88434	1.832079	21	-1.02852	0.964434
Complete	Post. frequency	S-A f — OJ f	-0.29831	1.501775	21	-0.19864	0.999999
Complete	Post. frequency	S-A f — J f	-1.18033	1.089338	21	-1.08353	0.953441
Complete	Post. frequency	S-A f — S-A m	1.127271	1.097741	21	1.0269	0.964728
Complete	Post. frequency	S-A f — I m	-2.87005	1.768647	21	-1.62274	0.732406
Complete	Post. frequency	S-A f — OJ m	0.079157	1.394137	21	0.056779	1
Complete	Post. frequency	S-A f — J m	-1.05567	1.130806	21	-0.93355	0.978895
Complete	Post. frequency	I f — OJ f	1.586025	1.991792	21	0.79628	0.991487
Complete	Post. frequency	I f — J f	0.704011	1.702517	21	0.413512	0.999868
Complete	Post. frequency	I f — S-A m	3.011607	1.707906	21	1.763333	0.649164
Complete	Post. frequency	I f — I m	-0.98571	2.200004	21	-0.44805	0.999774
Complete	Post. frequency	I f — OJ m	1.963494	1.911943	21	1.026963	0.964717
Complete	Post. frequency	I f — J m	0.828669	1.729344	21	0.479181	0.999649
Complete	Post. frequency	OJ f — J f	-0.88201	1.340664	21	-0.65789	0.997322
Complete	Post. frequency	OJ f — S-A m	1.425582	1.347501	21	1.057945	0.958808
Complete	Post. frequency	OJ f — I m	-2.57174	1.933607	21	-1.33002	0.877188
Complete	Post. frequency	OJ f — OJ m	0.377469	1.598231	21	0.236179	0.999997
Complete	Post. frequency	OJ f — J m	-0.75736	1.374571	21	-0.55098	0.999129
Complete	Post. frequency	J f — S-A m	2.307597	0.864341	21	2.669774	0.186547
Complete	Post. frequency	J f — I m	-1.68972	1.634063	21	-1.03406	0.963419
Complete	Post. frequency	J f — OJ m	1.259483	1.21888	21	1.033312	0.963558
Complete	Post. frequency	J f — J m	0.124659	0.905966	21	0.137598	1
Complete	Post. frequency	S-A m — I m	-3.99732	1.639677	21	-2.43787	0.274333
Complete	Post. frequency	S-A m — OJ m	-1.04811	1.226397	21	-0.85463	0.987162

Complete	Post. frequency	S-A m — J m	-2.18294	0.916053	21	-2.38298	0.298814
Complete	Post. frequency	I m — OJ m	2.949208	1.85125	21	1.593091	0.749154
Complete	Post. frequency	I m — J m	1.814384	1.661996	21	1.09169	0.951632
Complete	Post. frequency	OJ m — J m	-1.13482	1.25608	21	-0.90347	0.98241
**Dataset**	**Measure of feeding behavior**	**All individuals**	**numDF**	**denDF**	**F-value**	**p-value**	
Complete feeding sequences	Behavioral pattern	(Intercept)	1	274	39.48635	<.0001	
Complete	Behav. pattern	Age	3	107	10.38274	<.0001	
Complete	Behav. pattern	Sex	1	107	1.55758	0.2147	
Complete	Behav. pattern	Age:Sex	3	107	1.09243	0.3556	
**Complete feeding sequences**	**Behavioral pattern**	**contrast**	**estimate**	**SE**	**df**	**t.ratio**	**p-value**
Complete	Behav. pattern	S-A f — I f	-1.336	1.656	107	-0.807	0.9924
Complete	Behav. pattern	S-A f — OJ f	0.351	1.171	107	0.300	1.0000
Complete	Behav. pattern	S-A f — J f	-1.999	0.935	107	-2.139	0.3974
Complete	Behav. pattern	S-A f — S-A m	1.741	0.902	107	1.929	0.5342
Complete	Behav. pattern	S-A f — I m	-3.065	1.712	107	-1.791	0.6276
Complete	Behav. pattern	S-A f — OJ m	0.133	1.115	107	0.119	1.0000
Complete	Behav. pattern	S-A f — J m	-1.500	0.930	107	-1.613	0.7417
Complete	Behav. pattern	I f — OJ f	1.687	1.722	107	0.980	0.9764
Complete	Behav. pattern	I f — J f	-0.662	1.569	107	-0.422	0.9999
Complete	Behav. pattern	I f — S-A m	3.077	1.554	107	1.980	0.5001
Complete	Behav. pattern	I f — I m	-1.729	2.118	107	-0.816	0.9919
Complete	Behav. pattern	I f — OJ m	1.469	1.684	107	0.872	0.9879
Complete	Behav. pattern	I f — J m	-0.164	1.568	107	-0.104	1.0000

Complete	Behav. pattern	OJ f — J f	-2.350	1.048	107	-2.242	0.3359
Complete	Behav. pattern	OJ f — S-A m	1.390	1.022	107	1.360	0.8729
Complete	Behav. pattern	OJ f — I m	-3.416	1.774	107	-1.926	0.5365
Complete	Behav. pattern	OJ f — OJ m	-0.218	1.213	107	-0.180	1.0000
Complete	Behav. pattern	OJ f — J m	-1.851	1.045	107	-1.771	0.6408
Complete	Behav. pattern	J f — S-A m	3.739	0.739	107	5.059	<.0001
Complete	Behav. pattern	J f — I m	-1.067	1.626	107	-0.656	0.9979
Complete	Behav. pattern	J f — OJ m	2.132	0.986	107	2.161	0.3834
Complete	Behav. pattern	J f — J m	0.499	0.771	107	0.647	0.9981
Complete	Behav. pattern	S-A m — I m	-4.806	1.614	107	-2.977	0.0679
Complete	Behav. pattern	S-A m — OJ m	-1.608	0.957	107	-1.680	0.7002
Complete	Behav. pattern	S-A m — J m	-3.241	0.733	107	-4.421	0.0006
Complete	Behav. pattern	I m — OJ m	3.198	1.741	107	1.837	0.5967
Complete	Behav. pattern	I m — J m	1.565	1.629	107	0.961	0.9788
Complete	Behav. pattern	OJ m — J m	-1.633	0.980	107	-1.667	0.7085
**Dataset**	**Measure of feeding behavior**	**All individuals**	**numDF**	**denDF**	**F-value**	**p-value**	
First half of feeding sequence	Duration	(Intercept)	1	350	9.99283	0.0017	
First half	Duration	Age	3	119	9.38959	<0.0001	
First half	Duration	Sex	1	119	0.22853	0.6335	
First half	Duration	Age:Sex	3	119	1.48074	0.2233	
**First half of feeding sequence**	**Duration**	**contrast**	**estimate**	**SE**	**df**	**t.ratio**	**p-value**
First half	Duration	S-A f — I f	-0.404	0.621	119	-0.6512	0.998
First half	Duration	S-A f — OJ f	-0.526	0.414	119	-1.2715	0.908

First half	Duration	S-A f — J f	-0.837	0.331	119	-2.5258	0.195
First half	Duration	S-A f — S-A m	0.308	0.307	119	1.0032	0.973
First half	Duration	S-A f — I m	-0.932	0.562	119	-1.6582	0.714
First half	Duration	S-A f — OJ m	-1.182	0.405	119	-2.9224	0.077
First half	Duration	S-A f — J m	-0.544	0.327	119	-1.6634	0.711
First half	Duration	I f — OJ f	-0.122	0.650	119	-0.1874	1.000
First half	Duration	I f — J f	-0.432	0.601	119	-0.7200	0.996
First half	Duration	I f — S-A m	0.712	0.589	119	1.2098	0.928
First half	Duration	I f — I m	-0.527	0.751	119	-0.7024	0.997
First half	Duration	I f — OJ m	-0.778	0.645	119	-1.2064	0.929
First half	Duration	I f — J m	-0.140	0.600	119	-0.2331	1.000
First half	Duration	OJ f — J f	-0.310	0.384	119	-0.8095	0.992
First half	Duration	OJ f — S-A m	0.834	0.363	119	2.2962	0.304
First half	Duration	OJ f — I m	-0.406	0.595	119	-0.6819	0.997
First half	Duration	OJ f — OJ m	-0.656	0.449	119	-1.4624	0.826
First half	Duration	OJ f — J m	-0.018	0.380	119	-0.0470	1.000
First half	Duration	J f — S-A m	1.144	0.265	119	4.3105	0.001
First half	Duration	J f — I m	-0.095	0.540	119	-0.1760	1.000
First half	Duration	J f — OJ m	-0.346	0.374	119	-0.9245	0.983
First half	Duration	J f — J m	0.293	0.289	119	1.0139	0.972
First half	Duration	S-A m — I m	-1.239	0.526	119	-2.3569	0.272
First half	Duration	S-A m — OJ m	-1.490	0.352	119	-4.2269	0.001
First half	Duration	S-A m — J m	-0.852	0.260	119	-3.2776	0.029
First half	Duration	I m — OJ m	-0.251	0.588	119	-0.4258	1.000

First half	Duration	I m — J m	0.388	0.538	119	0.7204	0.996
First half	Duration	OJ m — J m	0.638	0.370	119	1.7238	0.672
**Dataset**	**Measure of feeding behavior**	**All individuals**	**numDF**	**denDF**	**F-value**	**p-value**	
First half of feeding sequence	Chew frequency	(Intercept)	1	52	16.32277	0.000177	
First half	Chew frequency	Age	3	18	1.167608	0.349566	
First half	Chew frequency	Sex	1	18	0.086314	0.772278	
First half	Chew frequency	Age:Sex	3	18	0.386278	0.764203	
**First half of feeding sequence**	**Chew Frequency**	**contrast**	**estimate**	**SE**	**df**	**t.ratio**	**p-value**
First half	Chew frequency	S-A f — I f	-2.51885	6.329276	18	-0.39797	0.999892
First half	Chew frequency	S-A f — OJ f	-6.5237	5.268731	18	-1.23819	0.909472
First half	Chew frequency	S-A f — J f	-4.48981	3.572242	18	-1.25686	0.903102
First half	Chew frequency	S-A f — S-A m	-1.83863	3.516502	18	-0.52286	0.999349
First half	Chew frequency	S-A f — I m	-1.68551	5.769797	18	-0.29213	0.999987
First half	Chew frequency	S-A f — OJ m	-7.35623	4.358585	18	-1.68776	0.694571
First half	Chew frequency	S-A f — J m	-1.37205	3.615408	18	-0.3795	0.999922
First half	Chew frequency	I f — OJ f	-4.00485	7.235626	18	-0.55349	0.99906
First half	Chew frequency	I f — J f	-1.97096	6.111928	18	-0.32248	0.999974
First half	Chew frequency	I f — S-A m	0.680221	6.079518	18	0.111887	1
First half	Chew frequency	I f — I m	0.833333	7.60824	18	0.10953	1
First half	Chew frequency	I f — OJ m	-4.83739	6.602425	18	-0.73267	0.994613
First half	Chew frequency	I f — J m	1.1468	6.137258	18	0.186859	0.999999
First half	Chew frequency	OJ f — J f	2.03389	5.005542	18	0.406328	0.999876

First half	Chew frequency	OJ f — S-A m	4.68507	4.965917	18	0.943445	0.976968
First half	Chew frequency	OJ f — I m	4.838183	6.751674	18	0.71659	0.995287
First half	Chew frequency	OJ f — OJ m	-0.83254	5.593908	18	-0.14883	1
First half	Chew frequency	OJ f — J m	5.15165	5.036439	18	1.022875	0.964551
First half	Chew frequency	J f — S-A m	2.65118	3.10833	18	0.852928	0.986875
First half	Chew frequency	J f — I m	2.804293	5.530505	18	0.507059	0.999466
First half	Chew frequency	J f — OJ m	-2.86643	4.036483	18	-0.71013	0.995539
First half	Chew frequency	J f — J m	3.117759	3.219799	18	0.968309	0.973487
First half	Chew frequency	S-A m — I m	0.153113	5.494667	18	0.027866	1
First half	Chew frequency	S-A m — OJ m	-5.51761	3.987238	18	-1.38382	0.853095
First half	Chew frequency	S-A m — J m	0.466579	3.157844	18	0.147752	1
First half	Chew frequency	I m — OJ m	-5.67072	6.068183	18	-0.9345	0.978136
First half	Chew frequency	I m — J m	0.313467	5.558485	18	0.056394	1
First half	Chew frequency	OJ m — J m	5.984186	4.074734	18	1.468608	0.813645
**Dataset**	**Measure of feeding behavior**	**All individuals**	**numDF**	**denDF**	**F-value**	**p-value**	
First half of feeding sequence	Chew number	(Intercept)	1	350	8.61787	0.0035	
First half	Chew number	Age	3	119	5.03184	0.0026	
First half	Chew number	Sex	1	119	1.44408	0.2319	
First half	Chew number	Age:Sex	3	119	2.46387	0.0658	
**First half of feeding sequence**	**Chew number**	**contrast**	**estimate**	**SE**	**df**	**t.ratio**	**p-value**
First half	Chew number	S-A f — I f	10.071	9.815	119	1.0261	0.9696
First half	Chew number	S-A f — OJ f	-6.399	6.492	119	-0.9857	0.9757

First half	Chew number	S-A f — J f	-11.030	5.203	119	-2.1201	0.4083
First half	Chew number	S-A f — S-A m	3.642	4.809	119	0.7572	0.9949
First half	Chew number	S-A f — I m	0.490	8.802	119	0.0556	1.0000
First half	Chew number	S-A f — OJ m	-16.200	6.333	119	-2.5580	0.1821
First half	Chew number	S-A f — J m	-0.258	5.123	119	-0.0504	1.0000
First half	Chew number	I f — OJ f	-16.470	10.278	119	-1.6025	0.7479
First half	Chew number	I f — J f	-21.102	9.505	119	-2.2200	0.3475
First half	Chew number	I f — S-A m	-6.429	9.315	119	-0.6902	0.9971
First half	Chew number	I f — I m	-9.582	11.836	119	-0.8095	0.9923
First half	Chew number	I f — OJ m	-26.271	10.184	119	-2.5797	0.1738
First half	Chew number	I f — J m	-10.329	9.483	119	-1.0892	0.9579
First half	Chew number	OJ f — J f	-4.631	6.026	119	-0.7686	0.9944
First half	Chew number	OJ f — S-A m	10.041	5.699	119	1.7618	0.6468
First half	Chew number	OJ f — I m	6.888	9.320	119	0.7391	0.9956
First half	Chew number	OJ f — OJ m	-9.801	7.029	119	-1.3943	0.8582
First half	Chew number	OJ f — J m	6.141	5.965	119	1.0294	0.9690
First half	Chew number	J f — S-A m	14.672	4.175	119	3.5142	0.0140
First half	Chew number	J f — I m	11.520	8.467	119	1.3606	0.8730
First half	Chew number	J f — OJ m	-5.169	5.860	119	-0.8822	0.9871
First half	Chew number	J f — J m	10.772	4.532	119	2.3770	0.2624
First half	Chew number	S-A m — I m	-3.152	8.238	119	-0.3827	0.9999
First half	Chew number	S-A m — OJ m	-19.842	5.516	119	-3.5968	0.0108
First half	Chew number	S-A m — J m	-3.900	4.070	119	-0.9582	0.9793
First half	Chew number	I m — OJ m	-16.689	9.213	119	-1.8116	0.6135

First half	Chew number	I m — J m	-0.748	8.427	119	-0.0887	1.0000
First half	Chew number	OJ m — J m	15.942	5.791	119	2.7528	0.1174
**Dataset**	**Measure of feeding behavior**	**All individuals**	**numDF**	**denDF**	**F-value**	**p-value**	
First half of feeding sequence	Ant. ingestion frequency	(Intercept)	1	425	48.48134	1.27E-11	
First half	Ant. frequency	Age	3	21	6.696263	0.002402	
First half	Ant. frequency	Sex	1	21	1.546411	0.22737	
First half	Ant. frequency	Age:Sex	3	21	0.730701	0.545196	
**First half of feeding sequence**	**Ant. ingestion frequency**	**contrast**	**estimate**	**SE**	**df**	**t.ratio**	**p-value**
First half	Ant. frequency	S-A f — I f	-0.03227	4.237038	21	-0.00762	1
First half	Ant. frequency	S-A f — OJ f	-4.86134	3.022822	21	-1.60821	0.740656
First half	Ant. frequency	S-A f — J f	-7.95166	2.364521	21	-3.36291	0.049101
First half	Ant. frequency	S-A f — S-A m	-0.1575	2.267022	21	-0.06947	1
First half	Ant. frequency	S-A f — I m	-0.38941	3.913951	21	-0.09949	1
First half	Ant. frequency	S-A f — OJ m	-4.56004	3.017201	21	-1.51135	0.793268
First half	Ant. frequency	S-A f — J m	-4.06415	2.427538	21	-1.67419	0.702574
First half	Ant. frequency	I f — OJ f	-4.82907	4.434885	21	-1.08888	0.95226
First half	Ant. frequency	I f — J f	-7.91939	4.015185	21	-1.97236	0.521306
First half	Ant. frequency	I f — S-A m	-0.12523	3.958552	21	-0.03164	1
First half	Ant. frequency	I f — I m	-0.35714	5.084266	21	-0.07024	1
First half	Ant. frequency	I f — OJ m	-4.52777	4.431055	21	-1.02183	0.965635
First half	Ant. frequency	I f — J m	-4.03189	4.052615	21	-0.99489	0.970179
First half	Ant. frequency	OJ f — J f	-3.09032	2.703086	21	-1.14326	0.939109
First half	Ant. frequency	OJ f — S-A m	4.703838	2.618225	21	1.796575	0.628886
First half	Ant. frequency	OJ f — I m	4.471926	4.127315	21	1.083495	0.953448

First half	Ant. frequency	OJ f — OJ m	0.301297	3.289257	21	0.0916	1
First half	Ant. frequency	OJ f — J m	0.797183	2.758379	21	0.289004	0.999988
First half	Ant. frequency	J f — S-A m	7.794163	1.818959	21	4.284959	0.006561
First half	Ant. frequency	J f — I m	7.56225	3.672633	21	2.059081	0.469647
First half	Ant. frequency	J f — OJ m	3.391622	2.696799	21	1.257647	0.904293
First half	Ant. frequency	J f — J m	3.887507	2.015481	21	1.928824	0.547764
First half	Ant. frequency	S-A m — I m	-0.23191	3.610632	21	-0.06423	1
First half	Ant. frequency	S-A m — OJ m	-4.40254	2.611733	21	-1.68568	0.695797
First half	Ant. frequency	S-A m — J m	-3.90666	1.900155	21	-2.05597	0.471472
First half	Ant. frequency	I m — OJ m	-4.17063	4.1232	21	-1.0115	0.96743
First half	Ant. frequency	I m — J m	-3.67474	3.713518	21	-0.98956	0.971024
First half	Ant. frequency	OJ m — J m	0.495886	2.752217	21	0.180177	1
**Dataset**	**Measure of feeding behavior**	**All individuals**	**numDF**	**denDF**	**F-value**	**p-value**	
First half of feeding sequence	Post. ingestion frequency	(Intercept)	1	159	36.79783	9.22E-09	
First half	Post. frequency	Age	3	21	4.644101	0.012136	
First half	Post. frequency	Sex	1	21	0.067303	0.797832	
First half	Post. frequency	Age:Sex	3	21	0.198005	0.896567	
**First half of feeding sequence**	**Post. ingestion frequency**	**contrast**	**estimate**	**SE**	**df**	**t.ratio**	**p-value**
First half	Post. frequency	S-A f — I f	-2.87038	2.137917	21	-1.3426	0.872082
First half	Post. frequency	S-A f — OJ f	-0.53704	1.793059	21	-0.29951	0.999985
First half	Post. frequency	S-A f — J f	-1.76252	1.316114	21	-1.33918	0.873481
First half	Post. frequency	S-A f — S-A m	0.948222	1.330364	21	0.712754	0.995616
First half	Post. frequency	S-A f — I m	-3.53704	2.07308	21	-1.70618	0.683616
First half	Post. frequency	S-A f — OJ m	-0.14142	1.655842	21	-0.08541	1

First half	Post. frequency	S-A f — J m	-1.96665	1.370158	21	-1.43534	0.831033
First half	Post. frequency	I f — OJ f	2.333333	2.296558	21	1.016013	0.966654
First half	Post. frequency	I f — J f	1.107857	1.947119	21	0.568972	0.998929
First half	Post. frequency	I f — S-A m	3.818599	1.956779	21	1.951471	0.533968
First half	Post. frequency	I f — I m	-0.66667	2.521265	21	-0.26442	0.999994
First half	Post. frequency	I f — OJ m	2.728958	2.191102	21	1.245473	0.908467
First half	Post. frequency	I f — J m	0.903729	1.984048	21	0.455498	0.999748
First half	Post. frequency	OJ f — J f	-1.22548	1.560655	21	-0.78523	0.99216
First half	Post. frequency	OJ f — S-A m	1.485265	1.572691	21	0.94441	0.977509
First half	Post. frequency	OJ f — I m	-3	2.236324	21	-1.34149	0.87254
First half	Post. frequency	OJ f — OJ m	0.395625	1.856152	21	0.213143	0.999999
First half	Post. frequency	OJ f — J m	-1.4296	1.606493	21	-0.88989	0.983844
First half	Post. frequency	J f — S-A m	2.710742	0.995214	21	2.723778	0.169638
First half	Post. frequency	J f — I m	-1.77452	1.875698	21	-0.94606	0.977292
First half	Post. frequency	J f — OJ m	1.621102	1.400854	21	1.157224	0.935383
First half	Post. frequency	J f — J m	-0.20413	1.047814	21	-0.19481	0.999999
First half	Post. frequency	S-A m — I m	-4.48527	1.885724	21	-2.37854	0.300858
First half	Post. frequency	S-A m — OJ m	-1.08964	1.414251	21	-0.77047	0.992995
First half	Post. frequency	S-A m — J m	-2.91487	1.065658	21	-2.73528	0.166202
First half	Post. frequency	I m — OJ m	3.395625	2.127886	21	1.595774	0.747652
First half	Post. frequency	I m — J m	1.570396	1.914006	21	0.820476	0.989857
First half	Post. frequency	OJ m — J m	-1.82523	1.451747	21	-1.25726	0.904426
**Dataset**	**Measure of feeding behavior**	**All individuals**	**numDF**	**denDF**	**F-value**	**p-value**	
First half	Behavioral Pattern	(Intercept)	1	219	46.37880	9.26E-11	
First half	Behav. Pattern	Age	3	99	6.66367	0.0004	

First half	Behav. Pattern	Sex	1	99	3.24445	0.0747	
First half	Behav. Pattern	Age:Sex	3	99	0.77050	0.5132	
**First half of feeding sequence**	**Post. ingestion frequency**	**contrast**	**estimate**	**SE**	**df**	**t.ratio**	**p-value**
First half	Behav. Pattern	S-A f — I f	0.458039	1.329575	99	0.3445	1.0000
First half	Behav. Pattern	S-A f — OJ f	-0.369827	0.933606	99	-0.3961	0.9999
First half	Behav. Pattern	S-A f — J f	-1.609762	0.772660	99	-2.0834	0.4328
First half	Behav. Pattern	S-A f — S-A m	1.286099	0.766078	99	1.6788	0.7007
First half	Behav. Pattern	S-A f — I m	-0.488170	1.324772	99	-0.3685	1.0000
First half	Behav. Pattern	S-A f — OJ m	-0.359959	0.899298	99	-0.4003	0.9999
First half	Behav. Pattern	S-A f — J m	-0.679258	0.775926	99	-0.8754	0.9876
First half	Behav. Pattern	I f — OJ f	-0.827865	1.342061	99	-0.6169	0.9986
First half	Behav. Pattern	I f — J f	-2.067801	1.233255	99	-1.6767	0.7021
First half	Behav. Pattern	I f — S-A m	0.828060	1.232189	99	0.6720	0.9975
First half	Behav. Pattern	I f — I m	-0.946208	1.633062	99	-0.5794	0.9990
First half	Behav. Pattern	I f — OJ m	-0.817998	1.315139	99	-0.6220	0.9985
First half	Behav. Pattern	I f — J m	-1.137297	1.237386	99	-0.9191	0.9835
First half	Behav. Pattern	OJ f —J f	-1.239935	0.794759	99	-1.5601	0.7725
First half	Behav. Pattern	OJ f — S-A m	1.655926	0.791934	99	2.0910	0.4279
First half	Behav. Pattern	OJ f — I m	-0.118343	1.337310	99	-0.0885	1.0000
First half	Behav. Pattern	OJ f — OJ m	0.009868	0.917759	99	0.0108	1.0000
First half	Behav. Pattern	OJ f — J m	-0.309431	0.798163	99	-0.3877	0.9999
First half	Behav. Pattern	J f — S-A m	2.895861	0.592750	99	4.8855	0.0001
First half	Behav. Pattern	J f — I m	1.121592	1.228921	99	0.9127	0.9842

First half	Behav. Pattern	J f — OJ m	1.249803	0.751489	99	1.6631	0.7106
First half	Behav. Pattern	J f — J m	0.930504	0.600546	99	1.5494	0.7786
First half	Behav. Pattern	S-A m — I m	-1.774269	1.229799	99	-1.4427	0.8350
First half	Behav. Pattern	S-A m — OJ m	-1.646058	0.750179	99	-2.1942	0.3642
First half	Behav. Pattern	S-A m — J m	-1.965357	0.597921	99	-3.2870	0.0293
First half	Behav. Pattern	I m — OJ m	0.128210	1.314598	99	0.0975	1.0000
First half	Behav. Pattern	I m — J m	-0.191088	1.232991	99	-0.1550	1.0000
First half	Behav. Pattern	OJ m — J m	-0.319299	0.755056	99	-0.4229	0.9999
**Dataset**	**Measure of feeding behavior**	**All individuals**	**numDF**	**denDF**	**F-value**	**p-value**	
Last half of feeding sequence	Duration	(Intercept)	1	357	107.46266	0.000	
Last half	Duration	Age	3	119	3.27558	0.0235	
Last half	Duration	Sex	1	119	0.88831	0.3478	
Last half	Duration	Age:Sex	3	119	2.70151	0.0487	
**Last half of feeding sequence**	**Duration**	**contrast**	**estimate**	**SE**	**df**	**t.ratio**	**p-value**
Last half	Duration	S-A f — I f	-0.1288	0.3609	119	-0.3568	1.0000
Last half	Duration	S-A f — OJ f	0.2024	0.2274	119	0.8903	0.9864
Last half	Duration	S-A f — J f	-0.2829	0.1869	119	-1.5136	0.7987
Last half	Duration	S-A f — S-A m	0.2405	0.1715	119	1.4026	0.8544
Last half	Duration	S-A f — I m	-0.4392	0.3135	119	-1.4012	0.8551
Last half	Duration	S-A f — OJ m	-0.2642	0.2237	119	-1.1810	0.9359
Last half	Duration	S-A f — J m	-0.0076	0.1824	119	-0.0417	1.0000
Last half	Duration	I f — OJ f	0.3312	0.3742	119	0.8850	0.9869

Last half	Duration	I f — J f	-0.1541	0.3507	119	-0.4394	0.9998
Last half	Duration	I f — S-A m	0.3693	0.3435	119	1.0752	0.9608
Last half	Duration	I f — I m	-0.3105	0.4308	119	-0.7207	0.9962
Last half	Duration	I f — OJ m	-0.1355	0.3723	119	-0.3639	1.0000
Last half	Duration	I f — J m	0.1212	0.3492	119	0.3470	1.0000
Last half	Duration	OJ f — J f	-0.4853	0.2116	119	-2.2938	0.3058
Last half	Duration	OJ f — S-A m	0.0381	0.1983	119	0.1922	1.0000
Last half	Duration	OJ f — I m	-0.6416	0.3289	119	-1.9511	0.5191
Last half	Duration	OJ f — OJ m	-0.4667	0.2449	119	-1.9058	0.5498
Last half	Duration	OJ f — J m	-0.2100	0.2078	119	-1.0104	0.9721
Last half	Duration	J f — S-A m	0.5234	0.1503	119	3.4822	0.0155
Last half	Duration	J f — I m	-0.1564	0.3022	119	-0.5173	0.9996
Last half	Duration	J f — OJ m	0.0186	0.2078	119	0.0896	1.0000
Last half	Duration	J f — J m	0.2753	0.1627	119	1.6924	0.6922
Last half	Duration	S-A m — I m	-0.6798	0.2932	119	-2.3186	0.2924
Last half	Duration	S-A m — OJ m	-0.5048	0.1941	119	-2.6003	0.1662
Last half	Duration	S-A m — J m	-0.2481	0.1445	119	-1.7167	0.6765
Last half	Duration	I m — OJ m	0.1750	0.3266	119	0.5357	0.9994
Last half	Duration	I m — J m	0.4316	0.2998	119	1.4396	0.8369
Last half	Duration	OJ m — J m	0.2566	0.2038	119	1.2593	0.9117
**Dataset**	**Measure of feeding behavior**	**All individuals**	**numDF**	**denDF**	**F-value**	**p-value**	
Last half of feeding sequence	Chew frequency	(Intercept)	1	1082	20.75188	<0.0001	
Last half	Chew frequency	Age	3	77	13.98314	<0.0001	

Last half	Chew frequency	Sex	1	77	5.11769	0.0265	
Last half	Chew frequency	Age:Sex	3	77	1.46138	0.2317	
**Last half of feeding sequence**	**Chew frequency**	**contrast**	**estimate**	**SE**	**df**	**t.ratio**	**p-value**
Last half	Chew frequency	S-A f — I f	-1.6358	1.9950	77	-0.8199	0.9915
Last half	Chew frequency	S-A f — OJ f	-3.7315	1.4874	77	-2.5088	0.2075
Last half	Chew frequency	S-A f — J f	-5.4839	1.1283	77	-4.8602	0.0002
Last half	Chew frequency	S-A f — S-A m	0.2700	1.0983	77	0.2458	1.0000
Last half	Chew frequency	S-A f — I m	-1.8728	1.8466	77	-1.0142	0.9709
Last half	Chew frequency	S-A f — OJ m	-3.2151	1.4332	77	-2.2432	0.3384
Last half	Chew frequency	S-A f — J m	-2.5487	1.1644	77	-2.1890	0.3699
Last half	Chew frequency	I f — OJ f	-2.0957	2.1187	77	-0.9891	0.9747
Last half	Chew frequency	I f — J f	-3.8480	1.8834	77	-2.0431	0.4605
Last half	Chew frequency	I f — S-A m	1.9058	1.8663	77	1.0211	0.9698
Last half	Chew frequency	I f — I m	-0.2370	2.3842	77	-0.0994	1.0000
Last half	Chew frequency	I f — OJ m	-1.5793	2.0806	77	-0.7591	0.9947
Last half	Chew frequency	I f — J m	-0.9129	1.9054	77	-0.4791	0.9997
Last half	Chew frequency	OJ f — J f	-1.7523	1.3347	77	-1.3129	0.8912
Last half	Chew frequency	OJ f — S-A m	4.0015	1.3085	77	3.0581	0.0582
Last half	Chew frequency	OJ f — I m	1.8587	1.9794	77	0.9390	0.9811
Last half	Chew frequency	OJ f — OJ m	0.5164	1.6006	77	0.3226	1.0000
Last half	Chew frequency	OJ f — J m	1.1828	1.3651	77	0.8665	0.9882
Last half	Chew frequency	J f — S-A m	5.7538	0.8806	77	6.5339	<0.0001
Last half	Chew frequency	J f — I m	3.6110	1.7256	77	2.0927	0.4289

Last half	Chew frequency	J f — OJ m	2.2688	1.2736	77	1.7815	0.6343
Last half	Chew frequency	J f — J m	2.9351	0.9611	77	3.0541	0.0588
Last half	Chew frequency	S-A m — I m	-2.1428	1.7065	77	-1.2556	0.9120
Last half	Chew frequency	S-A m — OJ m	-3.4850	1.2475	77	-2.7937	0.1117
Last half	Chew frequency	S-A m — J m	-2.8187	0.9260	77	-3.0440	0.0603
Last half	Chew frequency	I m — OJ m	-1.3423	1.9388	77	-0.6923	0.9970
Last half	Chew frequency	I m — J m	-0.6759	1.7495	77	-0.3863	0.9999
Last half	Chew frequency	OJ m — J m	0.6664	1.3057	77	0.5104	0.9996
**Dataset**	**Measure of feeding behavior**	**All individuals**	**numDF**	**denDF**	**F-value**	**p-value**	
Last half of feeding sequence	Chew number	(Intercept)	1	357	20.89064	6.71e-06	
Last half	Chew number	Age	3	119	3.85222	0.0113	
Last half	Chew number	Sex	1	119	0.21382	0.6446	
Last half	Chew number	Age:Sex	3	119	0.90246	0.4422	
**Last half of feeding sequence**	**Chew number**	**contrast**	**estimate**	**SE**	**df**	**t.ratio**	**p-value**
Last half	Chew number	S-A f — I f	1.8795	12.2197	119	0.1538	1.0000
Last half	Chew number	S-A f — OJ f	-10.9657	7.8598	119	-1.3952	0.8578
Last half	Chew number	S-A f — J f	-15.9206	6.4286	119	-2.4765	0.2158
Last half	Chew number	S-A f — S-A m	-0.4586	5.9248	119	-0.0774	1.0000
Last half	Chew number	S-A f — I m	-7.8495	10.8449	119	-0.7238	0.9961
Last half	Chew number	S-A f — OJ m	-16.0952	7.7615	119	-2.0737	0.4379
Last half	Chew number	S-A f — J m	-7.7980	6.3121	119	-1.2354	0.9196
Last half	Chew number	I f — OJ f	-12.8452	12.6937	119	-1.0119	0.9718

Last half	Chew number	I f — J f	-17.8002	11.8474	119	-1.5025	0.8047
Last half	Chew number	I f — S-A m	-2.3381	11.6055	119	-0.2015	1.0000
Last half	Chew number	I f — I m	-9.7291	14.6861	119	-0.6625	0.9978
Last half	Chew number	I f — OJ m	-17.9747	12.6419	119	-1.4218	0.8454
Last half	Chew number	I f — J m	-9.6776	11.8124	119	-0.8193	0.9917
Last half	Chew number	OJ f — J f	-4.9550	7.2913	119	-0.6796	0.9974
Last half	Chew number	OJ f — S-A m	10.5071	6.8568	119	1.5324	0.7884
Last half	Chew number	OJ f — I m	3.1161	11.3803	119	0.2738	1.0000
Last half	Chew number	OJ f — OJ m	-5.1295	8.4933	119	-0.6039	0.9988
Last half	Chew number	OJ f — J m	3.1676	7.1945	119	0.4403	0.9998
Last half	Chew number	J f — S-A m	15.4621	5.1586	119	2.9973	0.0635
Last half	Chew number	J f — I m	8.0711	10.4402	119	0.7731	0.9942
Last half	Chew number	J f — OJ m	-0.1746	7.1887	119	-0.0243	1.0000
Last half	Chew number	J f — J m	8.1226	5.5983	119	1.4509	0.8313
Last half	Chew number	S-A m — I m	-7.3910	10.1453	119	-0.7285	0.9960
Last half	Chew number	S-A m — OJ m	-15.6366	6.7430	119	-2.3189	0.2922
Last half	Chew number	S-A m — J m	-7.3395	5.0063	119	-1.4661	0.8237
Last half	Chew number	I m — OJ m	-8.2457	11.3192	119	-0.7285	0.9960
Last half	Chew number	I m — J m	0.0515	10.3800	119	0.0050	1.0000
Last half	Chew number	OJ m — J m	8.2972	7.0845	119	1.1712	0.9385
**Dataset**	**Measure of feeding behavior**	**All individuals**	**numDF**	**denDF**	**F-value**	**p-value**	
Last half of feeding sequence	Ant. ingestion frequency	(Intercept)	1	454	48.11229	<.0001	
Last half	Ant. frequency	Age	3	21	6.11283	0.0037	

Last half	Ant. frequency	Sex	1	21	1.15307	0.2951	
Last half	Ant. frequency	Age:Sex	3	21	0.76322	0.5273	
**Last half of feeding sequence**	**Ant. ingestion frequency**	**contrast**	**estimate**	**SE**	**df**	**t.ratio**	**p-value**
Last half	Ant. frequency	S-A f — I f	-0.0707	4.29	21	-0.016	1.0000
Last half	Ant. frequency	S-A f — OJ f	-5.1166	3.13	21	-1.636	0.7249
Last half	Ant. frequency	S-A f — J f	-8.0832	2.43	21	-3.330	0.0526
Last half	Ant. frequency	S-A f — S-A m	-0.5829	2.34	21	-0.249	1.0000
Last half	Ant. frequency	S-A f — I m	-0.7582	4.01	21	-0.189	1.0000
Last half	Ant. frequency	S-A f — OJ m	-4.6702	3.11	21	-1.504	0.7972
Last half	Ant. frequency	S-A f — J m	-4.2958	2.50	21	-1.717	0.6770
Last half	Ant. frequency	I f — OJ f	-5.0458	4.51	21	-1.118	0.9455
Last half	Ant. frequency	I f — J f	-8.0124	4.06	21	-1.974	0.5203
Last half	Ant. frequency	I f — S-A m	-0.5122	4.01	21	-0.128	1.0000
Last half	Ant. frequency	I f — I m	-0.6875	5.16	21	-0.133	1.0000
Last half	Ant. frequency	I f — OJ m	-4.5995	4.50	21	-1.023	0.9655
Last half	Ant. frequency	I f — J m	-4.2251	4.10	21	-1.030	0.9642
Last half	Ant. frequency	OJ f — J f	-2.9666	2.79	21	-1.061	0.9581
Last half	Ant. frequency	OJ f — S-A m	4.5336	2.72	21	1.669	0.7056
Last half	Ant. frequency	OJ f — I m	4.3583	4.24	21	1.028	0.9646
Last half	Ant. frequency	OJ f — OJ m	0.4464	3.40	21	0.131	1.0000
Last half	Ant. frequency	OJ f — J m	0.8208	2.86	21	0.287	1.0000
Last half	Ant. frequency	J f — S-A m	7.5002	1.87	21	4.015	0.0120
Last half	Ant. frequency	J f — I m	7.3249	3.76	21	1.951	0.5345
Last half	Ant. frequency	J f — OJ m	3.4129	2.77	21	1.232	0.9130
Last half	Ant. frequency	J f — J m	3.7873	2.07	21	1.829	0.6090

Last half	Ant. frequency	S-A m — I m	-0.1753	3.70	21	-0.047	1.0000
Last half	Ant. frequency	S-A m — OJ m	-4.0873	2.69	21	-1.519	0.7894
Last half	Ant. frequency	S-A m — J m	-3.7129	1.96	21	-1.891	0.5709
Last half	Ant. frequency	I m — OJ m	-3.9120	4.23	21	-0.926	0.9798
Last half	Ant. frequency	I m — J m	-3.5376	3.80	21	-0.930	0.9793
Last half	Ant. frequency	OJ m — J m	0.3744	2.84	21	0.132	1.0000
**Dataset**	**Measure of feeding behavior**	**All individuals**	**numDF**	**denDF**	**F-value**	**p-value**	
Last half of feeding sequence	Post. ingestion frequency	(Intercept)	1	202	41.75116	<.0001	
Last half	Post. frequency	Age	3	21	3.76449	0.0263	
Last half	Post. frequency	Sex	1	21	0.40204	0.5329	
Last half	Post. frequency	Age:Sex	3	21	0.30932	0.8184	
**Last half of feeding sequence**	**Post. ingestion frequency**	**contrast**	**estimate**	**SE**	**df**	**t.ratio**	**p-value**
Last half	Post. frequency	S-A f — I f	-1.8843	1.832	21	-1.029	0.9644
Last half	Post. frequency	S-A f — OJ f	-0.2983	1.502	21	-0.199	1.0000
Last half	Post. frequency	S-A f — J f	-1.1803	1.089	21	-1.084	0.9534
Last half	Post. frequency	S-A f — S-A m	1.1273	1.098	21	1.027	0.9647
Last half	Post. frequency	S-A f — I m	-2.8701	1.769	21	-1.623	0.7324
Last half	Post. frequency	S-A f — OJ m	0.0792	1.394	21	0.057	1.0000
Last half	Post. frequency	S-A f — J m	-1.0557	1.131	21	-0.934	0.9789
Last half	Post. frequency	I f — OJ f	1.5860	1.992	21	0.796	0.9915
Last half	Post. frequency	I f — J f	0.7040	1.703	21	0.414	0.9999
Last half	Post. frequency	I f — S-A m	3.0116	1.708	21	1.763	0.6492
Last half	Post. frequency	I f — I m	-0.9857	2.200	21	-0.448	0.9998
Last half	Post. frequency	I f — OJ m	1.9635	1.912	21	1.027	0.9647

Last half	Post. frequency	I f — J m	0.8287	1.729	21	0.479	0.9996
Last half	Post. frequency	OJ f — J f	-0.8820	1.341	21	-0.658	0.9973
Last half	Post. frequency	OJ f — S-A m	1.4256	1.348	21	1.058	0.9588
Last half	Post. frequency	OJ f — I m	2.5717	-1.934	21	-1.330	0.8772
Last half	Post. frequency	OJ f — OJ m	0.3775	1.598	21	0.236	1.0000
Last half	Post. frequency	OJ f — J m	-0.7574	1.375	21	-0.551	0.9991
Last half	Post. frequency	J f — S-A m	2.3076	0.864	21	2.670	0.1865
Last half	Post. frequency	J f — I m	-1.6897	1.634	21	-1.034	0.9634
Last half	Post. frequency	J f — OJ m	1.2595	1.219	21	1.033	0.9636
Last half	Post. frequency	J f — J m	0.1247	0.906	21	0.138	1.0000
Last half	Post. frequency	S-A m — I m	-3.9973	1.640	21	-2.438	0.2743
Last half	Post. frequency	S-A m — OJ m	-1.0481	1.226	21	-0.855	0.9872
Last half	Post. frequency	S-A m — J m	-2.1829	0.916	21	-2.383	0.2988
Last half	Post. frequency	I m — OJ m	2.9492	1.851	21	1.593	0.7492
Last half	Post. frequency	I m — J m	1.8144	1.662	21	1.092	0.9516
Last half	Post. frequency	OJ m — J m	-1.1348	1.256	21	-0.903	0.9824
**Dataset**	**Measure of feeding behavior**	**All individuals**	**numDF**	**denDF**	**F-value**	**p-value**	
Last half of feeding sequence	Behavioral pattern	(Intercept)	1	233	42.0096715	5.36E-10	
Last half	Behav. Pattern	Age	3	101	8.0772705	7.09E-05	
Last half	Behav. Pattern	Sex	1	101	0.6609444	0.4181	
Last half	Behav. Pattern	Age:Sex	3	101	0.7308839	0.5359	
**Behavioral Pattern**	**Behavioral Pattern**	**contrast**	**estimate**	**SE**	**df**	**t.ratio**	**p-value**

Last half	Behav. Pattern	S-A f — I f	-0.5740664	1.1191623	101	-0.5129	0.9996
Last half	Behav. Pattern	S-A f — OJ f	0.1842863	0.7670680	101	0.2402	1.0000
Last half	Behav. Pattern	S-A f — J f	-1.1495703	0.6317236	101	-1.8197	0.6083
Last half	Behav. Pattern	S-A f — S-A m	0.9011078	0.6238048	101	1.4445	0.8341
Last half	Behav. Pattern	S-A f — I m	-1.7108352	1.0922082	101	-1.5664	0.7689
Last half	Behav. Pattern	S-A f — OJ m	0.2984443	0.7331156	101	0.4071	0.9999
Last half	Behav. Pattern	S-A f — J m	-1.0203174	0.6291252	101	-1.6218	0.7361
Last half	Behav. Pattern	I f — OJ f	0.7583527	1.1359216	101	0.6676	0.9976
Last half	Behav. Pattern	I f — J f	-0.5755039	1.0471600	101	-0.5496	0.9993
Last half	Behav. Pattern	I f — S-A m	1.4751742	1.0441333	101	1.4128	0.8493
Last half	Behav. Pattern	I f — I m	-1.1367688	1.3719064	101	-0.8286	0.9911
Last half	Behav. Pattern	I f — OJ m	0.8725107	1.1110515	101	0.7853	0.9935
Last half	Behav. Pattern	I f — J m	-0.4462509	1.0466466	101	-0.4264	0.9999
Last half	Behav. Pattern	OJ f — J f	-1.3338566	0.6621981	101	-2.0143	0.4776
Last half	Behav. Pattern	OJ f — S-A m	0.7168215	0.6566423	101	1.0916	0.9572
Last half	Behav. Pattern	OJ f — I m	-1.8951215	1.1098962	101	-1.7075	0.6824
Last half	Behav. Pattern	OJ f — OJ m	0.1141580	0.7591573	101	0.1504	1.0000
Last half	Behav. Pattern	OJ f — J m	-1.2046037	0.6596714	101	-1.8261	0.6041
Last half	Behav. Pattern	J f — S-A m	2.0506781	0.4905545	101	4.1803	0.0016
Last half	Behav. Pattern	J f — I m	-0.5612649	1.0198907	101	-0.5503	0.9993
Last half	Behav. Pattern	J f — OJ m	1.4480146	0.6201574	101	2.3349	0.2854
Last half	Behav. Pattern	J f — J m	0.1292529	0.4938586	101	0.2617	1.0000
Last half	Behav. Pattern	S-A m — I m	-2.6119431	1.0184221	101	-2.5647	0.1814
Last half	Behav. Pattern	S-A m — OJ m	-0.6026635	0.6155309	101	-0.9791	0.9764

Last half	Behav. Pattern	S-A m — J m	-1.9214252	0.4877217	101	-3.9396	0.0036
Last half	Behav. Pattern	I m — OJ m	2.0092795	1.0877424	101	1.8472	0.5898
Last half	Behav. Pattern	I m — J m	0.6905179	1.0203544	101	0.6767	0.9974
Last half	Behav. Pattern	OJ m — J m	-1.3187617	0.6162800	101	-2.1399	0.3971

Contrast key: S-A = subadults-adults; OJ = older juveniles; J = juveniles; I = infants; m = males; f = females

SOM Table 5. Behavior types by food type and capuchin age and sex (continuous dataset).

**Age Category**	**Food (N items)**	**Sex (n items)**	**Behavioral Combinations (n items)**[1]
Subadults/adults	Almond (N = 42)	M (n = 29)	Ant. bite (-); Post. bite (-); Chew (-)<br>Ant. bite & post. bite (-)<br>**Ant. bite & chew (16)**<br>Post. bite & chew (-)<br>**Ant. bite, post. bite, & chew (13)**
		F (n = 13)	Ant. bite (-); Post. bite (-); Chew (-)<br>Ant. bite & post. bite (-)<br>**Ant. bite & chew (5)**<br>Post. bite & chew (-)<br>**Ant. bite, post. bite, & chew (8)**
	Gummy bear (N = 22)	M (n = 16)	**Ant. bite (1)**; Post. bite (-); Chew (-)<br>Ant. bite & post. bite (-)<br>**Ant. bite & chew (4)**<br>Post. bite & chew (-)<br>**Ant. bite, post. bite, & chew (11)**
		F (n = 6)	Ant. bite (-); Post. bite (-); Chew (-)<br>Ant. bite & post. bite (-)<br>Ant. bite & chew (-)<br>Post. bite & chew (-)<br>**Ant. bite, post. bite, & chew (6)**
	Peanut (N = 45)	M (n = 32)	Ant. bite (-); Post. bite (-); **Chew (2)**<br>Ant. bite & post. bite (-)<br>**Ant. bite & chew (27)**<br>Post. bite & chew (-)<br>**Ant. bite, post. bite, & chew (3)**

		F<br>(n = 13)	Ant. bite (-); Post. bite (-); Chew (-)<br>Ant. bite & post. bite (-)<br>**Ant. bite & chew (8)**<br>Post. bite & chew (-)<br>**Ant. bite, post. bite, & chew (5)**
	Popcorn<br>(N = 40)	M<br>(n = 29)	Ant. bite (-); Post. bite (-); **Chew (1)**<br>Ant. bite & post. bite (-)<br>**Ant. bite & chew (12)**<br>Post. bite & chew (-)<br>**Ant. bite, post. bite, & chew (16)**
		F<br>(n = 11)	Ant. bite (-); Post. bite (-); Chew (-)<br>Ant. bite & post. bite (-)<br>**Ant. bite & chew (4)**<br>Post. bite & chew (-)<br>**Ant. bite, post. bite, & chew (7)**
	Sunflower seeds<br>(N = 49)	M<br>(n = 35)	**Ant. bite (2)**; Post. bite (-); Chew (-)<br>Ant. bite & post. bite (-)<br>**Ant. bite & chew (28)**<br>Post. bite & chew (-)<br>**Ant. bite, post. bite, & chew (5)**
		F<br>(n = 14)	Ant. bite (-); Post. bite (-); Chew (-)<br>Ant. bite & post. bite (-)<br>**Ant. bite & chew (13)**<br>Post. bite & chew (-)<br>**Ant. bite, post. bite, & chew (1)**
Older Juveniles	Almond<br>(N = 19)	M<br>(n = 10)	Ant. bite (-); Post. bite (-); Chew (-)<br>Ant. bite & post. bite (-)

			**Ant. bite & chew (6)**<br>Post. bite & chew (-)<br>**Ant. bite, post. bite, & chew (4)**
		F<br>(n = 9)	Ant. bite (-); Post. bite (-); Chew (-)<br>Ant. bite & post. bite (-)<br>**Ant. bite & chew (9)**<br>Post. bite & chew (-)<br>Ant. bite, post. bite, & chew (-)
	Gummy bear<br>(N = 9)	M<br>(n = 4)	Ant. bite (-); Post. bite (-); Chew (-)<br>Ant. bite & post. bite (-)<br>Ant. bite & chew (-)<br>Post. bite & chew (-)<br>**Ant. bite, post. bite, & chew (4)**
		F<br>(n = 5)	**Ant. bite (1)**; Post. bite (-); Chew (-)<br>Ant. bite & post. bite (-)<br>**Ant. bite & chew (1)**<br>Post. bite & chew (-)<br>**Ant. bite, post. bite, & chew (3)**
	Peanut<br>(N = 24)	M<br>(n = 12)	Ant. bite (-); Post. bite (-); Chew (-)<br>Ant. bite & post. bite (-)<br>**Ant. bite & chew (8)**<br>Post. bite & chew (-)<br>**Ant. bite, post. bite, & chew (4)**
		F<br>(n = 12)	**Ant. bite (3)**; Post. bite (-); Chew (-)<br>Ant. bite & post. bite (-)<br>**Ant. bite & chew (9)**<br>Post. bite & chew (-)

			Ant. bite, post. bite, & chew (-)
	Popcorn (N = 18)	M (n = 11)	Ant. bite (-); Post. bite (-); Chew (-)
			Ant. bite & post. bite (-)
			Ant. bite & chew (-)
			**Post. bite & chew (1)**
			**Ant. bite, post. bite, & chew (10)**
		F (n = 7)	Ant. bite (-); Post. bite (-); Chew (-)
			Ant. bite & post. bite (-)
			Ant. bite & chew (-)
			Post. bite & chew (-)
			**Ant. bite, post. bite, & chew (7)**
	Sunflower seeds (N = 17)	M (n = 10)	**Ant. bite (1)**; Post. bite (-); Chew (-)
			Ant. bite & post. bite (-)
			**Ant. bite & chew (9)**
			Post. bite & chew (-)
			Ant. bite, post. bite, & chew (-)
		F (n = 7)	**Ant. bite (3)**; Post. bite (-); Chew (-)
			Ant. bite & post. bite (-)
			**Ant. bite & chew (4)**
			Post. bite & chew (-)
			Ant. bite, post. bite, & chew (-)
Juveniles	Almond (N = 43)	M (n = 20)	**Ant. bite (1)**; Post. bite (-); Chew (-)
			Ant. bite & post. bite (-)
			**Ant. bite & chew (7)**
			Post. bite & chew (-)
			**Ant. bite, post. bite, & chew (12)**
		F	Ant. bite (-); Post. bite (-); Chew (-)

		(n = 23)	Ant. bite & post. bite (-)<br>**Ant. bite & chew (5)**<br>Post. bite & chew (-)<br>**Ant. bite, post. bite, & chew (18)**
	Gummy bear (N = 17)	M (n = 7)	Ant. bite (-); Post. bite (-); Chew (-)<br>Ant. bite & post. bite (-)<br>Ant. bite & chew (-)<br>Post. bite & chew (-)<br>**Ant. bite, post. bite, & chew (7)**
		F (n = 10)	Ant. bite (-); Post. bite (-); Chew (-)<br>Ant. bite & post. bite (-)<br>**Ant. bite & chew (1)**<br>Post. bite & chew (-)<br>**Ant. bite, post. bite, & chew (9)**
	Peanut (N = 40)	M (n = 22)	Ant. bite (-); Post. bite (-); Chew (-)<br>Ant. bite & post. bite (-)<br>**Ant. bite & chew (13)**<br>Post. bite & chew (-)<br>**Ant. bite, post. bite, & chew (9)**
		F (n = 18)	**Ant. bite (1)**; Post. bite (-); Chew (-)<br>Ant. bite & post. bite (-)<br>**Ant. bite & chew (4)**<br>Post. bite & chew (-)<br>**Ant. bite, post. bite, & chew (13)**
	Popcorn (N = 36)	M (n = 21)	Ant. bite (-); Post. bite (-); Chew (-)<br>Ant. bite & post. bite (-)<br>**Ant. bite & chew (1)**

			Post. bite & chew (-)<br>**Ant. bite, post. bite, & chew (20)**
		F<br>(n = 15)	Ant. bite (-); Post. bite (-); Chew (-)<br>Ant. bite & post. bite (-)<br>Ant. bite & chew (-)<br>**Post. bite & chew (1)**<br>**Ant. bite, post. bite, & chew (14)**
	Sunflower seeds<br>(N = 36)	M<br>(n = 25)	**Ant. bite (4)**; Post. bite (-); Chew (-)<br>Ant. bite & post. bite (-)<br>**Ant. bite & chew (21)**<br>Post. bite & chew (-)<br>Ant. bite, post. bite, & chew (-)
		F<br>(n = 11)	**Ant. bite (1)**; Post. bite (-); Chew (-)<br>Ant. bite & post. bite (-)<br>**Ant. bite & chew (9)**<br>Post. bite & chew (-)<br>**Ant. bite, post. bite, & chew (1)**
Infants	Almond<br>(N = 7)	M<br>(n = 4)	Ant. bite (-); Post. bite (-); Chew (-)<br>Ant. bite & post. bite (-)<br>Ant. bite & chew (-)<br>Post. bite & chew (-)<br>**Ant. bite, post. bite, & chew (4)**
		F<br>(n = 3)	Ant. bite (-); Post. bite (-); Chew (-)<br>Ant. bite & post. bite (-)<br>Ant. bite & chew (-)<br>Post. bite & chew (-)<br>**Ant. bite, post. bite, & chew (3)**

	Gummy bear<br>(N = 4)	M<br>(n = 2)	Ant. bite (-); Post. bite (-); Chew (-)<br>Ant. bite & post. bite (-)<br>Ant. bite & chew (-)<br>Post. bite & chew (-)<br>**Ant. bite, post. bite, & chew (2)**
		F<br>(n = 2)	Ant. bite (-); Post. bite (-); Chew (-)<br>Ant. bite & post. bite (-)<br>Ant. bite & chew (-)<br>Post. bite & chew (-)<br>**Ant. bite, post. bite, & chew (2)**
	Peanut<br>(N = 7)	M<br>(n = 5)	Ant. bite (-); Post. bite (-); Chew (-)<br>Ant. bite & post. bite (-)<br>**Ant. bite & chew (1)**<br>Post. bite & chew (-)<br>**Ant. bite, post. bite, & chew (4)**
		F<br>(n = 2)	Ant. bite (-); Post. bite (-); Chew (-)<br>Ant. bite & post. bite (**1**)<br>Ant. bite & chew (**1**)<br>Post. bite & chew (-)<br>Ant. bite, post. bite, & chew (-)
	Sunflower seeds<br>(N = 6)	M<br>(n = 5)	Ant. bite (-); Post. bite (-); Chew (-)<br>Ant. bite & post. bite (-)<br>**Ant. bite & chew (5)**<br>Post. bite & chew (-)<br>Ant. bite, post. bite, & chew (-)
		F<br>(n = 1)	Ant. bite (-); Post. bite (-); Chew (-)<br>Ant. bite & post. bite (-)

			Ant. bite & chew (-)<br>Post. bite & chew (-)<br>**Ant. bite, post. bite, & chew (1)**

[1]Behavior Type combinations used during the feeding sequence: anterior bite alone; posterior bite alone; chew alone; anterior and posterior bite; anterior bite and chew; posterior bite and chew; and combined anterior bite, posterior bite, and chew. (-) = no instances; **(n)** = a non-zero number of instances.

SOM Table 6. Behavioral frequencies by food type and capuchin age category and sex (continuous dataset). The listed frequencies are the number of times each specific behavior was used for one food item. For example, for the first data row, S-A females ate 13 almonds, for which the number of anterior bites used in those 13 feeding sequences ranged from 1 to 22.

Behavior type	Frequency (per food item)	Food type	N items	Age	Sex
Anterior bite	1, 1, 1, 1, 1, 1, 2, 2, 3, 4, 5, 9, 22	Almond	13	S-A	F
Chew	1, 1, 3, 3, 3, 3, 3, 4, 6, 8, 9, 12, 20	Almond	13	S-A	F
Posterior bite	1, 2, 2, 2, 3, 3, 3, 4	Almond	13	S-A	F
Anterior bite	1, 1, 2, 2, 3, 3, 4, 4, 4, 5, 6, 6, 6, 7, 7, 7, 8, 8, 8, 9, 9, 10, 11, 11, 12, 13, 16, 22, 22	Almond	29	S-A	M
Chew	1, 1, 1, 3, 4, 4, 4, 4, 5, 5, 5, 5, 6, 6, 6, 6, 7, 7, 8, 8, 8, 9, 11, 11, 11, 13, 13, 18, 22	Almond	29	S-A	M
Posterior bite	1, 1, 1, 1, 1, 1, 1, 1, 2, 2, 2, 2, 3	Almond	29	S-A	M
Anterior bite	2, 4, 5, 7, 8, 10	Gummy bear	6	S-A	F
Chew	5, 6, 7, 7, 8, 9	Gummy bear	6	S-A	F
Posterior bite	2, 3, 3, 4, 5, 5	Gummy bear	6	S-A	F
Anterior bite	1, 2, 2, 2, 3, 4, 5, 5, 6, 8, 8, 10, 11, 12, 13, 20	Gummy bear	16	S-A	M
Chew	2, 3, 3, 3, 3, 4, 4, 5, 5, 6, 6, 9, 11, 12, 14	Gummy bear	16	S-A	M
Posterior bite	1, 1, 1, 1, 2, 2, 2, 2, 3, 5, 6	Gummy bear	16	S-A	M
Anterior bite	1, 1, 1, 1, 1, 2, 2, 2, 2, 3, 3, 5, 8	Peanut	13	S-A	F
Chew	1, 1, 1, 1, 1, 2, 2, 2, 3, 4, 4, 5, 6	Peanut	13	S-A	F
Posterior bite	1, 1, 2, 3	Peanut	13	S-A	F
Anterior bite	1, 1, 1, 1, 1, 1, 1, 1, 1, 1, 1, 1, 1, 1, 1, 1, 1, 1, 2, 2, 2, 2, 3, 3, 4, 4, 4, 5, 7	Peanut	32	S-A	M
Chew	1, 1, 1, 1, 1, 1, 1, 1, 1, 1, 1, 1, 1, 1, 1, 1, 2, 2, 2, 2, 2, 2, 2, 3, 3, 3, 3, 4, 4, 4, 6, 8	Peanut	32	S-A	M
Posterior bite	1, 1, 1	Peanut	32	S-A	M
Anterior bite	2, 2, 3, 3, 4, 4, 5, 6, 6, 9, 10	Popcorn	11	S-A	F
Chew	1, 1, 2, 2, 2, 3, 3, 4, 6, 9, 9	Popcorn	11	S-A	F

Posterior bite	1, 2, 2, 2, 2, 5, 9	Popcorn	11	S-A	F
Anterior bite	1, 1, 1, 1, 1, 1, 2, 2, 2, 2, 2, 2, 3, 3, 3, 3, 3, 3, 4, 5, 5, 5, 6, 7, 8, 8, 12, 16	Popcorn	29	S-A	M
Chew	1, 1, 1, 1, 1, 1, 1, 1, 1, 1, 1, 2, 2, 2, 2, 2, 2, 3, 3, 3, 3, 3, 4, 4, 5, 5, 5, 5, 15	Popcorn	29	S-A	M
Posterior bite	1, 1, 1, 1, 1, 1, 1, 1, 1, 1, 1, 2, 2, 2, 2, 3	Popcorn	29	S-A	M
Anterior bite	1, 1, 1, 1, 1, 1, 1, 1, 1, 1, 1, 1, 2, 2	Sunflower seed	14	S-A	F
Chew	1, 1, 1, 1, 1, 1, 1, 1, 1, 1, 1, 2, 2, 4	Sunflower seed	14	S-A	F
Posterior bite	1	Sunflower seed	14	S-A	F
Anterior bite	1, 1, 1, 1, 1, 1, 1, 1, 1, 1, 1, 1, 1, 1, 1, 1, 1, 1, 1, 1, 1, 1, 1, 1, 2, 2, 2, 2, 3, 3, 3, 4, 5, 5	Sunflower seed	35	S-A	M
Chew	1, 1, 1, 1, 1, 1, 1, 1, 1, 1, 1, 1, 1, 1, 1, 1, 1, 1, 1, 1, 1, 1, 1, 1, 1, 1, 1, 2, 3, 3, 3, 3, 5	Sunflower seed	35	S-A	M
Anterior bite	8, 10, 12, 17, 18, 21, 27, 30, 31	Almond	9	OJ	F
Chew	7, 10, 11, 14, 17, 18, 22, 22, 22	Almond	9	OJ	F
Anterior bite	11, 11, 11, 12, 13, 14, 19, 24, 29, 30	Almond	10	OJ	M
Chew	10, 11, 12, 12, 13, 15, 16, 24, 29, 29	Almond	10	OJ	M
Posterior bite	1, 2, 2, 3	Almond	10	OJ	M
Anterior bite	1, 3, 4, 7, 18	Gummybear	5	OJ	F
Chew	2, 4, 8, 22	Gummybear	5	OJ	F
Posterior bite	2, 5, 8	Gummybear	5	OJ	F
Anterior bite	5, 6, 10, 16	Gummybear	4	OJ	M
Chew	6, 10, 16, 22	Gummybear	4	OJ	M
Posterior bite	2, 5, 6, 10	Gummybear	4	OJ	M
Anterior bite	1, 1, 1, 2, 2, 4, 6, 7, 8, 8, 13, 16	Peanut	12	OJ	F
Chew	1, 1, 4, 5, 6, 7, 7, 11, 14	Peanut	12	OJ	F
Anterior bite	3, 3, 4, 4, 4, 5, 7, 8, 10, 11, 11, 13	Peanut	12	OJ	M
Chew	3, 4, 4, 4, 5, 7, 7, 8, 11, 12, 13, 13	Peanut	12	OJ	M
Posterior bite	1, 1, 1, 2	Peanut	12	OJ	M

Anterior bite	3, 3, 5, 6, 7, 8, 8	Popcorn	7	OJ	F
Chew	3, 4, 4, 5, 6, 8, 11	Popcorn	7	OJ	F
Posterior bite	1, 1, 2, 2, 2, 4, 5	Popcorn	7	OJ	F
Anterior bite	1, 1, 2, 2, 2, 3, 4, 5, 5, 6	Popcorn	11	OJ	M
Chew	2, 2, 2, 2, 2, 2, 2, 2, 3, 4, 4	Popcorn	11	OJ	M
Posterior bite	1, 1, 2, 2, 2, 2, 2, 3, 4, 5, 5	Popcorn	11	OJ	M
Anterior bite	1, 1, 1, 1, 2, 4, 5	Sunflower seed	7	OJ	F
Chew	1, 1, 4, 5	Sunflower seed	7	OJ	F
Anterior bite	1, 1, 1, 3, 4, 5, 5, 5, 6, 19	Sunflower seed	10	OJ	M
Chew	1, 1, 3, 4, 4, 4, 5, 5, 13	Sunflower seed	10	OJ	M
Anterior bite	4, 5, 5, 9, 9, 9, 11, 12, 13, 13, 16, 16, 17, 19, 19, 20, 23, 24, 27, 31, 34, 38, 40	Almond	23	J	F
Chew	3, 5, 6, 7, 9, 10, 12, 12, 13, 14, 14, 15, 16, 17, 17, 19, 19, 23, 26, 26, 28, 32, 38	Almond	23	J	F
Posterior bite	1, 1, 1, 1, 2, 3, 3, 3, 4, 4, 6, 7, 7, 7, 10, 11, 11, 13	Almond	23	J	F
Anterior bite	1, 5, 5, 6, 6, 7, 8, 8, 9, 10, 10, 11, 12, 13, 14, 18, 20, 23, 24, 29	Almond	20	J	M
Chew	1, 2, 3, 5, 5, 6, 6, 7, 9, 9, 10, 10, 10, 11, 13, 16, 18, 20, 21	Almond	20	J	M
Posterior bite	1, 1, 1, 1, 2, 3, 3, 4, 4, 6, 7, 8	Almond	20	J	M
Anterior bite	1, 1, 3, 4, 5, 8, 9, 9, 10, 10	Gummy bear	10	J	F
Chew	1, 3, 5, 5, 7, 8, 8, 9, 11, 12	Gummy bear	10	J	F
Posterior bite	1, 1, 3, 3, 4, 5, 5, 6, 8	Gummy bear	10	J	F
Anterior bite	2, 5, 7, 7, 10, 19, 34	Gummy bear	7	J	M
Chew	4, 4, 4, 7, 7, 12, 14	Gummy bear	7	J	M
Posterior bite	3, 3, 3, 5, 5, 7, 8	Gummy bear	7	J	M
Anterior bite	1, 2, 2, 3, 4, 4, 5, 5, 5, 6, 9, 11, 11, 20, 24, 32, 33, 39	Peanut	18	J	F
Chew	2, 2, 2, 3, 3, 6, 7, 7, 8, 9, 9, 9, 15, 18, 20, 22, 29	Peanut	18	J	F
Posterior bite	1, 1, 1, 1, 1, 2, 3, 3, 3, 3, 3, 5, 5	Peanut	18	J	F
Anterior bite	1, 1, 2, 2, 2, 2, 3, 3, 3, 4, 4, 5, 5, 6, 8, 10, 14, 16, 16, 18, 22, 31	Peanut	22	J	M

Chew	1, 1, 2, 3, 3, 3, 3, 4, 4, 4, 4, 4, 5, 5, 7, 8, 9, 12, 12, 16, 16, 16	Peanut	22	J	M
Posterior bite	1, 1, 2, 2, 3, 3, 3, 3, 4	Peanut	22	J	M
Anterior bite	2, 2, 3, 3, 5, 6, 6, 8, 8, 9, 9, 13, 16, 16	Popcorn	15	J	F
Chew	1, 2, 3, 3, 5, 5, 5, 5, 5, 7, 8, 12, 13, 13, 18	Popcorn	15	J	F
Posterior bite	1, 2, 2, 2, 3, 3, 3, 5, 6, 7, 7, 8, 9, 13, 15	Popcorn	15	J	F
Anterior bite	1, 2, 2, 3, 3, 3, 4, 4, 4, 5, 5, 5, 6, 6, 6, 7, 8, 8, 11, 13, 13	Popcorn	21	J	M
Chew	1, 1, 1, 1, 2, 2, 3, 3, 4, 4, 5, 5, 5, 7, 8, 9, 9, 9, 10, 14	Popcorn	21	J	M
Posterior bite	1, 1, 1, 1, 1, 2, 2, 2, 2, 3, 3, 4, 5, 5, 5, 5, 6, 6, 9, 10, 13	Popcorn	21	J	M
Anterior bite	1, 2, 3, 3, 4, 4, 4, 4, 6, 6, 12	Sunflower seed	11	J	F
Chew	1, 2, 2, 3, 3, 4, 4, 4, 4, 7	Sunflower seed	11	J	F
Posterior bite	2	Sunflower seed	11	J	F
Anterior bite	1, 1, 1, 1, 1, 1, 1, 1, 2, 2, 2, 2, 2, 3, 3, 3, 3, 4, 4, 5, 5, 5, 8, 9, 10	Sunflower seed	25	J	M
Chew	1, 1, 1, 1, 1, 1, 1, 1, 1, 1, 1, 2, 2, 2, 2, 2, 3, 4, 4, 4, 6	Sunflower seed	25	J	M
Anterior bite	2, 4, 4	Almond	3	I	F
Chew	9, 9, 12	Almond	3	I	F
Posterior bite	6, 7, 10	Almond	3	I	F
Anterior bite	1, 3, 9, 13	Almond	4	I	M
Chew	6, 9, 9, 15	Almond	4	I	M
Posterior bite	2, 6, 7, 8	Almond	4	I	M
Anterior bite	2, 7	Gummy bear	2	I	F
Chew	4, 4	Gummy bear	2	I	F
Posterior bite	3, 5	Gummy bear	2	I	F
Anterior bite	5, 7	Gummy bear	2	I	M
Chew	8, 9	Gummy bear	2	I	M
Posterior bite	8, 8	Gummy bear	2	I	M
Anterior bite	2, 4	Peanut	2	I	F
Chew	3	Peanut	2	I	F

Posterior bite	1	Peanut	2	I	F
Anterior bite	3, 5, 5, 5, 6	Peanut	5	I	M
Chew	5, 5, 6, 7, 9	Peanut	5	I	M
Posterior bite	3, 4, 5, 6	Peanut	5	I	M
Anterior bite	3	Sunflower seed	1	I	F
Chew	5	Sunflower seed	1	I	F
Posterior bite	1	Sunflower seed	1	I	F
Anterior bite	1, 1, 1, 1, 1	Sunflower seed	5	I	M
Chew	1, 1, 1, 1, 1	Sunflower seed	5	I	M

SOM Table 7. Results of LME models testing differences between age and sex groups and measures of feeding behavior using 10,000 randomly sampled feeding sequences (H5).

Dataset	Measure of feeding behavior	Contrasts	Percentage of significant tests
Random	Duration	(Intercept)	100%
Random	Duration	Age	38.99%
Random	Duration	Age:Sex	3.16%
Random	Duration	Sex	6.84%
Random	Duration	S-A f — S-A m	0.05%
Random	Duration	S-A f — I f	0%
Random	Duration	S-A f — I m	0%
Random	Duration	S-A f — OJ f	0.04%
Random	Duration	S-A f — OJ m	0%
Random	Duration	S-A f — J f	0.11%
Random	Duration	S-A f — J m	0%
Random	Duration	S-A m — I m	0.16%
Random	Duration	S-A m — OJ m	3.55%
Random	Duration	S-A m — J m	0.21%
Random	Duration	I f — S-A m	0%
Random	Duration	I f — I m	0.05%
Random	Duration	I f — OJ f	0.09%
Random	Duration	I f — OJ m	0.11%
Random	Duration	I f — J f	0.25%
Random	Duration	I f — J m	0.01%
Random	Duration	I m — OJ m	0%
Random	Duration	I m — J m	0%

Random	Duration	OJ f — S-A m	3.67%
Random	Duration	OJ f — I m	0%
Random	Duration	OJ f — OJ m	0%
Random	Duration	OJ f — J f	0%
Random	Duration	OJ f — J m	0%
Random	Duration	OJ m — J m	0%
Random	Duration	J f — S-A m	16.55%
Random	Duration	J f — I m	0%
Random	Duration	J f —OJ m	0%
Random	Duration	J f — J m	0.06%
Random	Behavioral Frequency: Anterior ingestion	(Intercept)	100%
Random	Behavioral Frequency: Anterior ingestion	Age	67.52%
Random	Behavioral Frequency: Anterior ingestion	Age:Sex	0%
Random	Behavioral Frequency: Anterior ingestion	Sex	0%
Random	Behavioral Frequency: Anterior ingestion	S-A f — S-A m	0%
Random	Behavioral Frequency: Anterior ingestion	S-A f — I f	0%
Random	Behavioral Frequency: Anterior ingestion	S-A f — I m	0%
Random	Behavioral Frequency: Anterior ingestion	S-A f — OJ f	0.05%
Random	Behavioral Frequency: Anterior ingestion	S-A f —OJ m	0%
Random	Behavioral Frequency: Anterior ingestion	S-A f — J f	1.3%
Random	Behavioral Frequency: Anterior ingestion	S-A f — J m	0%
Random	Behavioral Frequency: Anterior ingestion	S-A m — I m	0%
Random	Behavioral Frequency: Anterior ingestion	S-A m — OJ m	0%
Random	Behavioral Frequency: Anterior ingestion	S-A m — J m	0%

Random	Behavioral Frequency: Anterior ingestion	I f — S-A m	0%
Random	Behavioral Frequency: Anterior ingestion	I f — I m	0%
Random	Behavioral Frequency: Anterior ingestion	I f — OJ f	0%
Random	Behavioral Frequency: Anterior ingestion	I f — OJ m	0%
Random	Behavioral Frequency: Anterior ingestion	I f — J f	0%
Random	Behavioral Frequency: Anterior ingestion	I f — J m	0%
Random	Behavioral Frequency: Anterior ingestion	I m — OJ m	0%
Random	Behavioral Frequency: Anterior ingestion	I m — J m	0%
Random	Behavioral Frequency: Anterior ingestion	OJ f — S-A m	0%
Random	Behavioral Frequency: Anterior ingestion	OJ f — I m	0%
Random	Behavioral Frequency: Anterior ingestion	OJ f — OJ m	0%
Random	Behavioral Frequency: Anterior ingestion	OJ f — J f	0%
Random	Behavioral Frequency: Anterior ingestion	OJ f — J m	0%
Random	Behavioral Frequency: Anterior ingestion	OJ m — J m	0%
Random	Behavioral Frequency: Anterior ingestion	J f — S-A m	4.39%
Random	Behavioral Frequency: Anterior ingestion	J f — I m	0%
Random	Behavioral Frequency: Anterior ingestion	J f — OJ m	0%
Random	Behavioral Frequency: Anterior ingestion	J f — J m	0%
Random	Behavioral Frequency: Posterior ingestion	(Intercept)	100%
Random	Behavioral Frequency: Posterior ingestion	Age	65.02%
Random	Behavioral Frequency: Posterior ingestion	Age:Sex	0.08%
Random	Behavioral Frequency: Posterior ingestion	Sex	1.14%
Random	Behavioral Frequency: Posterior ingestion	S-A f — S-A m	0%
Random	Behavioral Frequency: Posterior ingestion	S-A f — I f	0.08%
Random	Behavioral Frequency: Posterior ingestion	S-A f — I m	0.8%

Random	Behavioral Frequency: Posterior ingestion	S-A f — OJ f	0%
Random	Behavioral Frequency: Posterior ingestion	S-A f — OJ m	0%
Random	Behavioral Frequency: Posterior ingestion	S-A f — J f	0.01%
Random	Behavioral Frequency: Posterior ingestion	S-A f — J m	0%
Random	Behavioral Frequency: Posterior ingestion	S-A m — I m	8.27%
Random	Behavioral Frequency: Posterior ingestion	S-A m — OJ m	0%
Random	Behavioral Frequency: Posterior ingestion	S-A m — J m	0.66%
Random	Behavioral Frequency: Posterior ingestion	I f — S-A m	0.88%
Random	Behavioral Frequency: Posterior ingestion	I f — I m	0%
Random	Behavioral Frequency: Posterior ingestion	I f — OJ f	0.01%
Random	Behavioral Frequency: Posterior ingestion	I f — OJ m	0.06%
Random	Behavioral Frequency: Posterior ingestion	I f — J f	0.01%
Random	Behavioral Frequency: Posterior ingestion	I f — J m	0%
Random	Behavioral Frequency: Posterior ingestion	I m — OJ m	0.43%
Random	Behavioral Frequency: Posterior ingestion	I m — J m	0.18%
Random	Behavioral Frequency: Posterior ingestion	OJ f — S-A m	0%
Random	Behavioral Frequency: Posterior ingestion	OJ f — I m	0.14%
Random	Behavioral Frequency: Posterior ingestion	OJ f — OJ m	0%
Random	Behavioral Frequency: Posterior ingestion	OJ f — J f	0%
Random	Behavioral Frequency: Posterior ingestion	OJ f — J m	0%
Random	Behavioral Frequency: Posterior ingestion	OJ m — J m	0%
Random	Behavioral Frequency: Posterior ingestion	J f — S-A m	10.28%
Random	Behavioral Frequency: Posterior ingestion	J f — I m	0.05%
Random	Behavioral Frequency: Posterior ingestion	J f — OJ m	0%
Random	Behavioral Frequency: Posterior ingestion	J f — J m	0%

Random	Behavioral Frequency: Chew	(Intercept)	100%
Random	Behavioral Frequency: Chew	Age	46.14%
Random	Behavioral Frequency: Chew	Age:Sex	0%
Random	Behavioral Frequency: Chew	Sex	1.17%
Random	Behavioral Frequency: Chew	S-A f — S-A m	0%
Random	Behavioral Frequency: Chew	S-A f — I f	0%
Random	Behavioral Frequency: Chew	S-A f — I m	0%
Random	Behavioral Frequency: Chew	S-A f — OJ f	0.12%
Random	Behavioral Frequency: Chew	S-A f — OJ m	0.01%
Random	Behavioral Frequency: Chew	S-A f — J f	0.72%
Random	Behavioral Frequency: Chew	S-A f — J m	0%
Random	Behavioral Frequency: Chew	S-A m — I m	0%
Random	Behavioral Frequency: Chew	S-A m — OJ m	0.07%
Random	Behavioral Frequency: Chew	S-A m — J m	0%
Random	Behavioral Frequency: Chew	I f — S-A m	0%
Random	Behavioral Frequency: Chew	I f — I m	0%
Random	Behavioral Frequency: Chew	I f — OJ f	0%
Random	Behavioral Frequency: Chew	I f — OJ m	0%
Random	Behavioral Frequency: Chew	I f — J f	0%
Random	Behavioral Frequency: Chew	I f — J m	0%
Random	Behavioral Frequency: Chew	I m — OJ m	0%
Random	Behavioral Frequency: Chew	I m — J m	0%
Random	Behavioral Frequency: Chew	OJ f — S-A m	0.46%
Random	Behavioral Frequency: Chew	OJ f — I m	0%
Random	Behavioral Frequency: Chew	OJ f — OJ m	0%

Random	Behavioral Frequency: Chew	OJ f — J f	0%
Random	Behavioral Frequency: Chew	OJ f — J m	0%
Random	Behavioral Frequency: Chew	OJ m — J m	0%
Random	Behavioral Frequency: Chew	J f — S-A m	15.11%
Random	Behavioral Frequency: Chew	J f — I m	0%
Random	Behavioral Frequency: Chew	J f — OJ m	0%
Random	Behavioral Frequency: Chew	J f — J m	0.02%
Random	Chew Number	(Intercept)	100%
Random	Chew Number	Age	42.44%
Random	Chew Number	Age:Sex	0.57%
Random	Chew Number	Sex	8.44%
Random	Chew Number	S-A f — S-A m	0%
Random	Chew Number	S-A f — I f	0%
Random	Chew Number	S-A f —I m	0%
Random	Chew Number	S-A f — OJ f	0.07%
Random	Chew Number	S-A f — OJ m	0.09%
Random	Chew Number	S-A f — J f	0.13%
Random	Chew Number	S-A f — J m	0%
Random	Chew Number	S-A m — I m	0%
Random	Chew Number	S-A m — OJ m	1.81%
Random	Chew Number	S-A m — J m	0%
Random	Chew Number	I f — S-A m	0%
Random	Chew Number	I f — I m	0%
Random	Chew Number	I f — OJ f	0.15%
Random	Chew Number	I f — OJ m	0.06%

Random	Chew Number	I f — J f	0.1%
Random	Chew Number	I f — J m	0%
Random	Chew Number	I m — OJ m	0.01%
Random	Chew Number	I m — J m	0%
Random	Chew Number	OJ f — S-A m	1.14%
Random	Chew Number	OJ f — I m	0%
Random	Chew Number	OJ f — OJ m	0%
Random	Chew Number	OJ f — J f	0%
Random	Chew Number	OJ f — J m	0.21%
Random	Chew Number	OJ m — J m	0.35%
Random	Chew Number	J f — S-A m	4.72%
Random	Chew Number	J f — I m	0%
Random	Chew Number	J f — OJ m	0%
Random	Chew Number	J f — J m	0.71%
Random	Unique Behavioral pattern	(Intercept)	100%
Random	Unique Behavioral pattern	Age	99.9%
Random	Unique Behavioral pattern	Age:Sex	1.17%
Random	Unique Behavioral pattern	Sex	4.03%
Random	Unique Behavioral pattern	S-A f —S-A m	0.06%
Random	Unique Behavioral pattern	S-A f — I f	0%
Random	Unique Behavioral pattern	S-A f — I m	0.3%
Random	Unique Behavioral pattern	S-A f — OJ f	0%
Random	Unique Behavioral pattern	S-A f — OJ m	0%
Random	Unique Behavioral pattern	S-A f —J f	3.44%
Random	Unique Behavioral pattern	S-A f — J m	0.46%

Random	Unique Behavioral pattern	S-A m — I m	10%
Random	Unique Behavioral pattern	S-A m — OJ m	0%
Random	Unique Behavioral pattern	S-A m — J m	82.02%
Random	Unique Behavioral pattern	I f — S-A m	0.11%
Random	Unique Behavioral pattern	I f —I m	0.01%
Random	Unique Behavioral pattern	I f — OJ f	0%
Random	Unique Behavioral pattern	I f — OJ m	0%
Random	Unique Behavioral pattern	I f — J f	0.01%
Random	Unique Behavioral pattern	I f — J m	0%
Random	Unique Behavioral pattern	I m — OJ m	0.45%
Random	Unique Behavioral pattern	I m — J m	0.01%
Random	Unique Behavioral pattern	OJ f — S-A m	0%
Random	Unique Behavioral pattern	OJ f — I m	0.44%
Random	Unique Behavioral pattern	OJ f — OJ m	0%
Random	Unique Behavioral pattern	OJ f — J f	3.02%
Random	Unique Behavioral pattern	OJ f — J m	0.24%
Random	Unique Behavioral pattern	OJ m —J m	0.26%
Random	Unique Behavioral pattern	J f — S-A m	96.48%
Random	Unique Behavioral pattern	J f — I m	0%
Random	Unique Behavioral pattern	J f — OJ m	3.62%
Random	Unique Behavioral pattern	J f — J m	0.02%

Contrast key: S-A = Subadults-adults; OJ = Older Juveniles; J = juveniles; I = infants; m = males; f = females

SOM Table 8. Measures of feeding behaviors within each food type (by sex and age).

	. Variation by Sex[1]					
**Food type**	**Chew number**	**Anterior Ingestion Frequency**	**Posterior Ingestion Frequency**	**Chew Frequency**	**Duration**	**Behavioral Patterns**
Almond	*	*	*	*	*	*
Gummy bear	*	*	*	*	*	*
Peanut	*	*	*	*	*	*
Popcorn kernel	*	*	*	*	J (0.024)	*
Sunflower seed	*	*	*	*	*	*
	**II. Variation by Age[1]**					
**Food type**	**Chew number**	**Anterior Ingestion Frequency**	**Posterior Ingestion Frequency**	**Chew Frequency**	**Duration**	**Behavioral Patterns**
Almond	*	All (0.006)<br>J / S-A (0.037)<br>OJ / S-A (0.018)	*	All (0.025)<br>OJ / S-A (0.029)	*	All (0.027)<br>S-A / J (0.029)
Gummy bear	*	*	*	*	*	*
Peanut	All (0.041)<br>S-A / J (0.029)	All (0.021)<br>J / S-A (0.013)	*	All (0.016)<br>J / S-A (0.011)	*	*
Popcorn kernel	*	*	All (0.049)	*	*	All (0.036)<br>S-A / J (0.033)
Sunflower seed	*	All (0.020)<br>J / S-A (0.039)	*	All (0.0143)<br>OJ / S-A (0.009)	*	All (0.017)<br>S-A / I (0.023)

SOM Table 9. Results of LME models testing differences in age (H2).

**Dataset**	**Measure of feeding behavior**	**All individuals**	**numDF**	**denDF**	**F-value**	**p-value**	
Complete feeding sequences	Duration	(Intercept)	1	357	106.80825	<.0001	
Complete	Duration	Age	3	123	3.66626	0.0142	
**Complete feeding sequences**	**Duration**	**contrast**	**estimate**	**SE**	**df**	**t.ratio**	**p-value**
Complete	Duration	S-A — I	-0.4507	0.236	123	-1.913	0.2281
Complete	Duration	S-A — OJ	-0.2656	0.152	123	-1.749	0.3032
Complete	Duration	S-A — J	-0.3559	0.117	123	-3.037	0.0153
Complete	Duration	I — OJ	0.1851	0.256	123	0.724	0.8873
Complete	Duration	I — J	0.0949	0.237	123	0.401	0.9781
Complete	Duration	OJ — J	-0.0903	0.154	123	-0.588	0.9355
**Dataset**	**Measure of feeding behavior**	**All individuals**	**numDF**	**denDF**	**F-value**	**p-value**	
Complete feeding sequences	Chew frequency	(Intercept)	1	439	59.25446	9.26E-14	
Complete	Chew frequency	Age	3	25	6.184645	0.002723	
**Complete feeding sequences**	**Chew frequency**	**contrast**	**estimate**	**SE**	**df**	**t.ratio**	**p-value**
Complete	Chew frequency	S-A — I	-2.4803	2.319542	25	-1.06931	0.710911
Complete	Chew frequency	S-A — OJ	-4.82554	1.65807	25	-2.91034	0.035262
Complete	Chew frequency	S-A — J	-4.90445	1.218384	25	-4.02537	0.002456
Complete	Chew frequency	I — OJ	-2.34524	2.574632	25	-0.9109	0.799228
Complete	Chew frequency	I — J	-2.42414	2.316029	25	-1.04668	0.724096
Complete	Chew frequency	OJ — J	-0.0789	1.653153	25	-0.04773	0.99996
**Dataset**	**Measure of feeding behavior**	**All individuals**	**numDF**	**denDF**	**F-value**	**p-value**	

Complete feeding sequences	Chew numbers	(Intercept)	1	357	14.521592	0.0002	
Complete	Chew numbers	Age	3	123	4.628892	0.004	
**Complete feeding sequences**	**Chew numbers**	**contrast**	**estimate**	**SE**	**df**	**t.ratio**	**p-value**
Complete	Chew numbers	S-A — I	-1.11	13.03	123	-0.085	0.9998
Complete	Chew numbers	S-A — OJ	-26.60	8.55	123	-3.113	0.0122
Complete	Chew numbers	S-A — J	-18.82	6.55	123	-2.875	0.0243
Complete	Chew numbers	I — OJ	-25.49	14.19	123	-1.797	0.2799
Complete	Chew numbers	I — J	-17.71	13.07	123	-1.355	0.5301
Complete	Chew numbers	OJ — J	7.78	8.62	123	0.902	0.8039
**Dataset**	**Measure of feeding behavior**	**All individuals**	**numDF**	**denDF**	**F-value**	**p-value**	
Complete feeding sequences	Ant. ingestion frequency	(Intercept)	1	454	48.81076	1.01E-11	
Complete	Ant. frequency	Age	3	25	6.325814	0.002426	
**Complete feeding sequences**	**Ant. ingestion frequency**	**contrast**	**estimate**	**SE**	**df**	**t.ratio**	**p-value**
Complete	Ant. frequency	S-A — I	-0.0252	2.728348	25	-0.00924	1
Complete	Ant. frequency	S-A — OJ	-4.46875	1.958964	25	-2.28118	0.129613
Complete	Ant. frequency	S-A — J	-5.92189	1.444047	25	-4.10089	0.002032
Complete	Ant. frequency	I — OJ	-4.44355	3.027046	25	-1.46795	0.470961
Complete	Ant. frequency	I — J	-5.89669	2.722267	25	-2.16609	0.16046
Complete	Ant. frequency	OJ — J	-1.45314	1.950486	25	-0.74501	0.877902
**Dataset**	**Measure of feeding behavior**	**All individuals**	**numDF**	**denDF**	**F-value**	**p-value**	
Complete feeding sequences	Post. ingestion frequency	(Intercept)	1	202	43.59064	3.50E-10	
Complete	Post. frequency	Age	3	25	4.209186	0.015344	
**Complete feeding**	**Post. ingestion**	**contrast**	**estimate**	**SE**	**df**	**t.ratio**	**p-value**

**sequences**	**frequency**						
Complete	Post. frequency	S-A — I	-3.17276	1.149534	25	-2.76004	0.048999
Complete	Post. frequency	S-A — OJ	-0.85706	0.896442	25	-0.95607	0.775111
Complete	Post. frequency	S-A — J	-1.90804	0.647228	25	-2.94801	0.032422
Complete	Post. frequency	I — OJ	2.315698	1.284869	25	1.802283	0.295883
Complete	Post. frequency	I — J	1.264721	1.125249	25	1.123948	0.678517
Complete	Post. frequency	OJ — J	-1.05098	0.86508	25	-1.21489	0.623444
**Dataset**	**Measure of feeding behavior**	**All individuals**	**numDF**	**denDF**	**F-value**	**p-value**	
Complete feeding sequences	Behavioral pattern	(Intercept)	1	274	38.94121	<.0001	
Complete	Behav. pattern	Age	3	111	10.29879	<.0001	
**Complete feeding sequences**	**Behavioral pattern**	**contrast**	**estimate**	**SE**	**df**	**t.ratio**	**p-value**
Complete	Behav. pattern	S-A — I	-3.377	1.150	111	-2.936	0.0208
Complete	Behav. pattern	S-A — OJ	-0.988	0.735	111	-1.344	0.5372
Complete	Behav. pattern	S-A — J	-2.965	0.568	111	-5.216	<.0001
Complete	Behav. pattern	I — OJ	2.389	1.230	111	1.942	0.2165
Complete	Behav. pattern	I — J	0.412	1.137	111	0.363	0.9836
Complete	Behav. pattern	OJ — J	-1.976	0.718	111	-2.752	0.0344
**Dataset**	**Measure of feeding behavior**	**All individuals**	**numDF**	**denDF**	**F-value**	**p-value**	
First half of the feeding sequence	Duration	(Intercept)	1	350	9.921051	0.0018	
First half	Duration	Age	3	123	9.344731	1.30e-05	
**First half of the feeding sequence**	**Duration**	**contrast**	**estimate**	**SE**	**df**	**t.ratio**	**p-value**
First half	Duration	S-A — I	-0.9166	0.4012	123	-2.2847	0.1072
First half	Duration	S-A — OJ	-1.0843	0.2646	123	-4.0973	0.0004

First half	Duration	S-A — J	-0.9035	0.2009	123	-4.4971	0.0001
First half	Duration	I — OJ	-0.1676	0.4381	123	-0.3826	0.9809
First half	Duration	I — J	0.0131	0.4026	123	0.0326	1.0000
First half	Duration	OJ — J	0.1808	0.2669	123	0.6773	0.9055
**Dataset**	**Measure of feeding behavior**	**All individuals**	**numDF**	**denDF**	**F-value**	**p-value**	
First half of the feeding sequence	Chew frequency	(Intercept)	1	52	17.01553	0.000134	
First half	Chew frequency	Age	3	22	1.292379	0.301879	
**First half of the feeding sequence**	**Chew frequency**	**contrast**	**estimate**	**SE**	**df**	**t.ratio**	**p-value**
First half	Chew frequency	S-A — I	-0.87739	3.937647	22	-0.22282	0.995979
First half	Chew frequency	S-A — OJ	-5.87999	3.01119	22	-1.95271	0.23598
First half	Chew frequency	S-A — J	-1.86247	2.212102	22	-0.84195	0.833931
First half	Chew frequency	I — OJ	-5.0026	4.40135	22	-1.13661	0.671468
First half	Chew frequency	I — J	-0.98508	3.898463	22	-0.25268	0.99417
First half	Chew frequency	OJ — J	4.017515	2.959766	22	1.357376	0.537969
**Dataset**	**Measure of feeding behavior**	**All individuals**	**numDF**	**denDF**	**F-value**	**p-value**	
First half of the feeding sequence	Chew numbers	(Intercept)	1	350	8.3583	0.0041	
First half	Chew numbers	Age	3	123	4.8216	0.0033	
**First half of the feeding sequence**	**Chew numbers**	**contrast**	**estimate**	**SE**	**df**	**t.ratio**	**p-value**
First half	Chew numbers	S-A — I	2.1720	6.4305	123	0.3378	0.9867
First half	Chew numbers	S-A — OJ	-14.1056	4.2325	123	-3.3327	0.0062
First half	Chew numbers	S-A — J	-7.9615	3.2154	123	-2.4760	0.0688
First half	Chew numbers	I — OJ	-16.2777	7.0198	123	-2.3188	0.0993

First half	Chew numbers	I — J	-10.1336	6.4529	123	-1.5704	0.3993
First half	Chew numbers	OJ — J	6.1441	4.2695	123	1.4391	0.4776
**Dataset**	**Measure of feeding behavior**	**All individuals**	**numDF**	**denDF**	**F-value**	**p-value**	
First half of the feeding sequence	Ant. ingestion frequency	(Intercept)	1	425	48.95324	1.03E-11	
First half	Ant. frequency	Age	3	25	6.867997	0.001571	
**First half of the feeding sequence**	**Ant. ingestion frequency**	**contrast**	**estimate**	**SE**	**df**	**t.ratio**	**p-value**
First half	Ant. frequency	S-A — I	-0.11511	2.690474	25	-0.04279	0.999971
First half	Ant. frequency	S-A — OJ	-4.59523	1.904723	25	-2.41254	0.100536
First half	Ant. frequency	S-A — J	-6.03099	1.409132	25	-4.27993	0.001293
First half	Ant. frequency	I —OJ	-4.48011	2.976857	25	-1.50498	0.449612
First half	Ant. frequency	I — J	-5.91587	2.686887	25	-2.20176	0.15033
First half	Ant. frequency	OJ — J	-1.43576	1.899653	25	-0.7558	0.873326
**Dataset**	**Measure of feeding behavior**	**All individuals**	**numDF**	**denDF**	**F-value**	**p-value**	
First half of the feeding sequence	Post. ingestion frequency	(Intercept)	1	159	39.03221	3.66E-09	
First half	Post. frequency	Age	3	25	5.27703	0.005865	
**First half of the feeding sequence**	**Post. ingestion frequency**	**contrast**	**estimate**	**SE**	**df**	**t.ratio**	**p-value**
First half	Post. frequency	S-A — I	-3.88871	1.310186	25	-2.96806	0.030998
First half	Post. frequency	S-A — OJ	-0.97925	1.034753	25	-0.94636	0.780376
First half	Post. frequency	S-A — J	-2.534	0.749811	25	-3.37951	0.011954
First half	Post. frequency	I — OJ	2.909461	1.461791	25	1.99034	0.218388
First half	Post. frequency	I — J	1.354717	1.276063	25	1.061638	0.715397
First half	Post. frequency	OJ — J	-1.55474	0.991193	25	-1.56856	0.413924
**Dataset**	**Measure of feeding behavior**	**All individuals**	**numDF**	**denDF**	**F-value**	**p-value**	

First half of the feeding sequence	Behavioral pattern	(Intercept)	1	219	45.48563	1.35E-10	
First half	Behav. pattern	Age	3	103	6.57243	0.0004	
**First half of the feeding sequence**	**Behavioral pattern**	**contrast**	**estimate**	**SE**	**df**	**t.ratio**	**p-value**
First half	Behav. pattern	S-A — I	-0.9228	0.8996	103	-1.0257	0.7348
First half	Behav. pattern	S-A — OJ	-1.2786	0.5814	103	-2.1992	0.1303
First half	Behav. pattern	S-A — J	-2.0623	0.4662	103	-4.4241	0.0001
First half	Behav. pattern	I — OJ	-0.3558	0.9460	103	-0.3761	0.9818
First half	Behav. pattern	I — J	-1.1395	0.8795	103	-1.2957	0.5678
First half	Behav. pattern	OJ — J	-0.7837	0.5505	103	-1.4237	0.4876
**Dataset**	**Measure of feeding behavior**	**All individuals**	**numDF**	**denDF**	**F-value**	**p-value**	
Last half of the feeding sequence	Duration	(Intercept)	1	357	105.20393	0.000	
Last half	Duration	Age	3	123	3.11993	0.0285	
**Last half of the feeding sequence**	**Duration**	**contrast**	**estimate**	**SE**	**df**	**t.ratio**	**p-value**
Last half	Duration	S-A — I	-0.4797	0.2322	123	-2.0660	0.1701
Last half	Duration	S-A — OJ	-0.2083	0.1485	123	-1.4022	0.5004
Last half	Duration	S-A — J	-0.3065	0.1150	123	-2.6664	0.0426
Last half	Duration	I — OJ	0.2714	0.2515	123	1.0791	0.7029
Last half	Duration	I — J	0.1732	0.2332	123	0.7426	0.8796
Last half	Duration	OJ — J	-0.0983	0.1502	123	-0.6541	0.9139
**Dataset**	**Measure of feeding behavior**	**All individuals**	**numDF**	**denDF**	**F-value**	**p-value**	
Last half of the feeding sequence	Chew frequency	(Intercept)	1	436	58.51418	1.30E-13	
Last half	Chew frequency	Age	3	25	6.227808	0.002628	

Last half of the feeding sequence	Chew frequency	contrast	estimate	SE	df	t.ratio	p-value
Last half	Chew frequency	S-A — I	-2.51316	2.332203	25	-1.07759	0.706048
Last half	Chew frequency	S-A — OJ	-4.85807	1.66804	25	-2.91244	0.035098
Last half	Chew frequency	S-A — J	-4.96103	1.227039	25	-4.04309	0.00235
Last half	Chew frequency	I — OJ	-2.34491	2.588808	25	-0.90579	0.801895
Last half	Chew frequency	I — J	-2.44787	2.329205	25	-1.05095	0.721621
Last half	Chew frequency	OJ — J	-0.10296	1.663846	25	-0.06188	0.999913
**Dataset**	**Measure of feeding behavior**	**All individuals**	**numDF**	**denDF**	**F-value**	**p-value**	
Last half of the feeding sequence	Chew numbers	(Intercept)	1	357	20.58037	7.82e-06	
Last half	Chew numbers	Age	3	123	3.86497	0.0111	
**Last half of the feeding sequence**	**Chew numbers**	**contrast**	**estimate**	**SE**	**df**	**t.ratio**	**p-value**
Last half	Chew numbers	S-A — I	-3.3204	7.7809	123	-0.4267	0.9738
Last half	Chew numbers	S-A — OJ	-13.2492	5.0168	123	-2.6410	0.0455
Last half	Chew numbers	S-A — J	-11.3396	3.8707	123	-2.9296	0.0208
Last half	Chew numbers	I — OJ	-9.9288	8.4431	123	-1.1760	0.6431
Last half	Chew numbers	I — J	-8.0193	7.8127	123	-1.0264	0.7343
Last half	Chew numbers	OJ — J	1.9096	5.0711	123	0.3766	0.9817
**Dataset**	**Measure of feeding behavior**	**All individuals**	**numDF**	**denDF**	**F-value**	**p-value**	
Last half of the feeding sequence	Ant. ingestion frequency	(Intercept)	1	39	5.054162	0.030294	
Last half	Ant. frequency	Age	3	17	0.725256	0.550789	
**Last half of the feeding sequence**	**Ant. ingestion frequency**	**contrast**	**estimate**	**SE**	**df**	**t.ratio**	**p-value**
Last half	Ant. frequency	S-A — I	1.082757	5.286804	17	0.204804	0.99684

Last half	Ant. frequency	S-A — OJ	1.214586	4.355508	17	0.278862	0.992146
Last half	Ant. frequency	S-A — J	-3.51294	3.396772	17	-1.0342	0.732227
Last half	Ant. frequency	I — OJ	0.13183	5.701863	17	0.02312	0.999995
Last half	Ant. frequency	I — J	-4.5957	5.007879	17	-0.91769	0.795824
Last half	Ant. frequency	OJ — J	-4.72753	4.012356	17	-1.17824	0.648076
**Dataset**	**Measure of feeding behavior**	**All individuals**	**numDF**	**denDF**	**F-value**	**p-value**	
Last half of the feeding sequence	Post. ingestion frequency	(Intercept)	1	155	34.81111	2.21E-08	
Last half	Post. frequency	Age	3	25	2.707536	0.066714	
**Last half of the feeding sequence**	**Post. ingestion frequency**	**contrast**	**estimate**	**SE**	**df**	**t.ratio**	**p-value**
Last half	Post. frequency	S-A — I	-2.03276	1.137543	25	-1.78697	0.302904
Last half	Post. frequency	S-A — OJ	-1.03474	0.859151	25	-1.20438	0.629854
Last half	Post. frequency	S-A — J	-1.66969	0.624741	25	-2.67261	0.059054
Last half	Post. frequency	I — J	0.998016	1.25731	25	0.79377	0.856583
Last half	Post. frequency	I — OJ	0.363071	1.110401	25	0.326973	0.987636
Last half	Post. frequency	OJ — J	-0.63494	0.822878	25	-0.77162	0.866471
**Dataset**	**Measure of feeding behavior**	**All individuals**	**numDF**	**denDF**	**F-value**	**p-value**	
Last half of the feeding sequence	Behavioral pattern	(Intercept)	1	233	41.41092	6.95E-10	
Last half	Behav. pattern	Age	3	105	8.27067	5.44E-05	
**Last half of the feeding sequence**	**Behavioral pattern**	**contrast**	**estimate**	**SE**	**df**	**t.ratio**	**p-value**
Last half	Behav. pattern	S-A — I	-1.79639	0.74081	105	-2.4249	0.0786
Last half	Behav. pattern	S-A — OJ	-0.38846	0.47006	105	-0.8264	0.8418
Last half	Behav. pattern	S-A — J	-1.72205	0.37592	105	-4.5808	0.0001
Last half	Behav. pattern	I — OJ	1.40793	0.77931	105	1.8066	0.2760

Last half	Behav. pattern	I — J	0.07434	0.72581	105	0.1024	0.9996
Last half	Behav. pattern	OJ — J	-1.33359	0.44605	105	-2.9897	0.0180

Contrast key: S-A = Subadults-adults; OJ = Older Juveniles; J = juveniles; I = infants

SOM Table 10. Results LME models testing differences in age and measures of feeding behavior in of 10,000 randomly sampled feeding sequences. (H5)

Dataset	Measure of feeding behavior	Contrasts	Percentage of significant tests
Random	Duration	(Intercept)	100.00%
Random	Duration	Age	38.80%
Random	Duration	S-A — I	0.12%
Random	Duration	S-A — OJ	31.14%
Random	Duration	S-A — J	14.38%
Random	Duration	I — OJ	0.03%
Random	Duration	I — J	0.00%
Random	Duration	OJ — J	0.09%
Random	Behavioral Frequency: Anterior ingestion	(Intercept)	100.00%
Random	Behavioral Frequency: Anterior ingestion	Age	77.91%
Random	Behavioral Frequency: Anterior ingestion	S-A — I	0.00%
Random	Behavioral Frequency: Anterior ingestion	S-A — OJ	2.03%
Random	Behavioral Frequency: Anterior ingestion	S-A — J	63.35%
Random	Behavioral Frequency: Anterior ingestion	I — OJ	0.09%
Random	Behavioral Frequency: Anterior ingestion	I — J	0.14%
Random	Behavioral Frequency: Anterior ingestion	OJ — J	0.00%
Random	Behavioral Frequency: Posterior ingestion	(Intercept)	100.00%
Random	Behavioral Frequency: Posterior ingestion	Age	74.58%
Random	Behavioral Frequency: Posterior ingestion	S-A — I	41.08%
Random	Behavioral Frequency: Posterior ingestion	S-A — OJ	0.00%
Random	Behavioral Frequency: Posterior ingestion	S-A — J	45.29%

Random	Behavioral Frequency: Posterior ingestion	I — OJ	5.95%
Random	Behavioral Frequency: Posterior ingestion	I — J	0.94%
Random	Behavioral Frequency: Posterior ingestion	OJ — J	0.09%
Random	Behavioral Frequency: Chew	(Intercept)	100.00%
Random	Behavioral Frequency: Chew	Age	53.42%
Random	Behavioral Frequency: Chew	S-A — I	0.00%
Random	Behavioral Frequency: Chew	S-A — OJ	16.62%
Random	Behavioral Frequency: Chew	S-A — J	47.46%
Random	Behavioral Frequency: Chew	I — OJ	0.00%
Random	Behavioral Frequency: Chew	I — J	0.00%
Random	Behavioral Frequency: Chew	OJ — J	0.01%
Random	Chew Number	(Intercept)	100.00%
Random	Chew Number	Age	43.16%
Random	Chew Number	S-A — I	0.00%
Random	Chew Number	S-A — OJ	26.34%
Random	Chew Number	S-A — J	1.77%
Random	Chew Number	I — OJ	8.60%
Random	Chew Number	I — J	0.61%
Random	Chew Number	OJ — J	0.85%
Random	Unique Behavioral pattern	(Intercept)	100.00%
Random	Unique Behavioral pattern	Age	99.94%
Random	Unique Behavioral pattern	S-A — I	21.23%
Random	Unique Behavioral pattern	S-A — OJ	0.01%
Random	Unique Behavioral pattern	S-A — J	99.85%

Random	Unique Behavioral pattern	I — OJ	3.18%
Random	Unique Behavioral pattern	I — J	0.00%
Random	Unique Behavioral pattern	OJ — J	56.09%

Contrast key: S-A = Subadults-adults; OJ = Older Juveniles; J = juveniles; I = infants

SOM Table 11. Results of LME models testing differences in each measure of feeding behavior varies with food volume (H3).

Dataset	Measure of feeding behavior	All individuals	numDF	denDF	F-value	p-value	
Complete	Duration	(Intercept)	1	455	1909.5093	<.0001	
Complete	Duration	Age	3	25	4.0490	0.0178	
Complete	Duration	logVolume	1	455	195.6800	<.0001	
Complete	Duration	Age:Volume	3	455	3.2637	0.0213	
**Complete feeding sequence**	**Duration**	**contrast**	**estimate**	**SE**	**df**	**t.ratio**	**p-value**
Complete	Duration	S-A — I	-0.2612	0.306	25	-0.854	0.8283
Complete	Duration	S-A — OJ	-0.2454	0.211	25	-1.162	0.6554
Complete	Duration	S-A — J	-0.4476	0.158	25	-2.841	0.0411
Complete	Duration	I — OJ	0.0158	0.337	25	0.047	1.0000
Complete	Duration	I — J	-0.1864	0.306	25	-0.610	0.9281
Complete	Duration	OJ — J	-0.2022	0.211	25	-0.959	0.7737
**Dataset**	**Measure of feeding behavior**	**All individuals**	**numDF**	**denDF**	**F-value**	**p-value**	
Complete	Chew frequency	(Intercept)	1	435	178.1989	<.0001	
Complete	Chew frequency	Age	3	25	7.691857	0.000834	
Complete	Chew frequency	logVolume	1	435	161.1051	<.0001	
Complete	Chew frequency	Age:Volume	3	435	8.023221	3.25E-05	
**Complete feeding sequence**	**Chew frequency**	**contrast**	**estimate**	**SE**	**df**	**t.ratio**	**p-value**
Complete	Chew frequency	S-A — I	-1.71875	2.090935	25	-0.822	0.843517
Complete	Chew frequency	S-A — OJ	-5.80376	1.524554	25	-3.80686	0.004232
Complete	Chew frequency	S-A — J	-4.78453	1.119839	25	-4.27251	0.001318
Complete	Chew frequency	I — OJ	-4.08501	2.324869	25	-1.75709	0.31691
Complete	Chew frequency	I — J	-3.06578	2.082016	25	-1.4725	0.468315

Complete	Chew frequency	OJ — J	1.019236	1.512298	25	0.673965	0.905944
**Dataset**	**Measure of feeding behavior**	**All individuals**	**numDF**	**denDF**	**F-value**	**p-value**	
Complete	Chew numbers	(Intercept)	1	455	223.99185	<.0001	
Complete	Chew numbers	Age	3	25	4.27487	0.0144	
Complete	Chew numbers	logVolume	1	455	234.13068	<.0001	
Complete	Chew numbers	Age:Volume	3	455	9.00655	<.0001	
**Complete feeding sequence**	**Chew numbers**	**contrast**	**estimate**	**SE**	**df**	**t.ratio**	**p-value**
Complete	Chew numbers	S-A — I	-0.575	17.66	25	-0.033	1.0000
Complete	Chew numbers	S-A — OJ	-22.195	12.51	25	-1.775	0.3086
Complete	Chew numbers	S-A — J	-27.322	9.23	25	-2.960	0.0315
Complete	Chew numbers	I — OJ	-21.620	19.54	25	-1.107	0.6889
Complete	Chew numbers	I — J	-26.747	17.62	25	-1.518	0.4423
Complete	Chew numbers	OJ — J	-5.127	12.46	25	-0.412	0.9760
**Dataset**	**Measure of feeding behavior**	**All individuals**	**numDF**	**denDF**	**F-value**	**p-value**	
Complete	Ant. frequency	(Intercept)	1	450	139.4528	<0.001	
Complete	Ant. frequency	Age	3	25	7.662518	0.000852	
Complete	Ant. frequency	logVolume	1	450	107.0009	<0.001	
Complete	Ant. frequency	Age:Volume	3	450	5.602082	0.000888	
**Complete feeding sequence**	**Ant. ingestion frequency**	**contrast**	**estimate**	**SE**	**df**	**t.ratio**	**p-value**
Complete	Ant. frequency	S-A — I	0.740057	2.496618	25	0.296424	0.990715
Complete	Ant. frequency	S-A — OJ	-5.61153	1.822236	25	-3.07948	0.024085
Complete	Ant. frequency	S-A — J	-6.06717	1.344286	25	-4.5133	0.000715
Complete	Ant. frequency	I — OJ	-6.35159	2.773353	25	-2.29022	0.127411
Complete	Ant. frequency	I — J	-6.80723	2.485568	25	-2.7387	0.051299

Complete	Ant. frequency	OJ — J	-0.45564	1.807067	25	-0.25214	0.994227
**Dataset**	**Measure of feeding behavior**	**All individuals**	**numDF**	**denDF**	**F-value**	**p-value**	
Complete	Post. frequency	(Intercept)	1	198	132.6972	0	
Complete	Post. frequency	Age	3	25	4.224315	0.015129	
Complete	Post. frequency	logVolume	1	198	1.414828	0.235681	
Complete	Post. frequency	Age:Volume	3	198	3.96482	0.00897	
**Complete feeding sequence**	**Post. ingestion frequency**	**contrast**	**estimate**	**SE**	**df**	**t.ratio**	**p-value**
Complete	Post. frequency	S-A — I	-3.0143	1.15055	25	-2.61988	0.065972
Complete	Post. frequency	S-A — OJ	-1.32852	0.950391	25	-1.39786	0.512301
Complete	Post. frequency	S-A — J	-1.69476	0.659521	25	-2.56969	0.073212
Complete	Post. frequency	I — OJ	1.685784	1.318184	25	1.278869	0.584356
Complete	Post. frequency	I — J	1.319539	1.126647	25	1.171209	0.650024
Complete	Post. frequency	OJ — J	-0.36625	0.92131	25	-0.39753	0.978245
**Dataset**	**Measure of feeding behavior**	**All individuals**	**numDF**	**denDF**	**F-value**	**p-value**	
Complete	Behavioral pattern	(Intercept)	1	360	336.8469	<.0001	
Complete	Behav. pattern	Age	3	25	5.9597	0.0033	
Complete	Behav. pattern	logVolume	1	360	34.4965	<.0001	
Complete	Behav. pattern	Age:Volume	3	360	0.7122	0.5452	
**Complete feeding sequence**	**Behavioral pattern**	**contrast**	**estimate**	**SE**	**df**	**t.ratio**	**p-value**
Complete	Behav. pattern	S-A — I	-2.347	1.544	25	-1.520	0.4410
Complete	Behav. pattern	S-A — OJ	-0.768	1.030	25	-0.745	0.8779
Complete	Behav. pattern	S-A — J	-3.023	0.766	25	-3.949	0.0030
Complete	Behav. pattern	I — OJ	1.580	1.680	25	0.940	0.7836
Complete	Behav. pattern	I — J	-0.676	1.532	25	-0.441	0.9707

Complete	Behav. pattern	OJ — J	-2.256	1.012	25	-2.229	0.1430
**Dataset**	**Measure of feeding behavior**	**All individuals**	**numDF**	**denDF**	**F-value**	**p-value**	
First half	Duration	(Intercept)	1	448	181.30063	0.000	
First half	Duration	Age	3	25	8.74102	0.0004	
First half	Duration	logVolume	1	448	186.26033	0.000	
First half	Duration	Age:Volume	3	448	1.40164	0.2417	
**First half of the feeding sequence**	**Duration**	**contrast**	**estimate**	**SE**	**df**	**t.ratio**	**p-value**
First half	Duration	S-A — I	-0.6003	0.5047	25	-1.1893	0.6390
First half	Duration	S-A — OJ	-1.0791	0.3500	25	-3.0828	0.0239
First half	Duration	S-A — J	-1.0739	0.2599	25	-4.1311	0.0019
First half	Duration	I — OJ	-0.4788	0.5561	25	-0.8610	0.8246
First half	Duration	I — J	-0.4736	0.5043	25	-0.9391	0.7843
First half	Duration	OJ — J	0.0052	0.3495	25	0.0150	1.0000
**Dataset**	**Measure of feeding behavior**	**All individuals**	**numDF**	**denDF**	**F-value**	**p-value**	
First half	Chew frequency	(Intercept)	1	48	104.6556	1.20E-13	
First half	Chew frequency	Age	3	22	1.624005	0.212518	
First half	Chew frequency	logVolume	1	48	17.64329	0.000115	
First half	Chew frequency	Age:Volume	3	48	0.974832	0.412422	
**First half of the feeding sequence**	**Chew frequency**	**contrast**	**estimate**	**SE**	**df**	**t.ratio**	**p-value**
First half	Chew frequency	S-A — I	-1.18214	3.686807	22	-0.32064	0.988283
First half	Chew frequency	S-A — OJ	-7.13388	2.785431	22	-2.56114	0.077726
First half	Chew frequency	S-A — J	-3.22981	2.117855	22	-1.52504	0.440251

First half	Chew frequency	I — OJ	-5.95174	4.112176	22	-1.44735	0.484712
First half	Chew frequency	I — J	-2.04767	3.692787	22	-0.55451	0.944328
First half	Chew frequency	OJ — J	3.904071	2.793342	22	1.397634	0.513948
**Dataset**	**Measure of feeding behavior**	**All individuals**	**numDF**	**denDF**	**F-value**	**p-value**	
First half	Chew numbers	(Intercept)	1	448	97.09722	0	
First half	Chew numbers	Age	3	25	3.43795	0.0321	
First half	Chew numbers	logVolume	1	448	186.12672	0	
First half	Chew numbers	Age:Volume	3	448	5.91464	0.0006	
**First half of the feeding sequence**	**Chew numbers**	**contrast**	**estimate**	**SE**	**df**	**t.ratio**	**p-value**
First half	Chew numbers	S-A — I	1.2195	8.8664	25	0.1375	0.9990
First half	Chew numbers	S-A — OJ	-11.2892	6.3176	25	-1.7869	0.3029
First half	Chew numbers	S-A — J	-11.5100	4.6422	25	-2.4795	0.0880
First half	Chew numbers	I — OJ	-12.5087	9.8297	25	-1.2725	0.5882
First half	Chew numbers	I — J	-12.7295	8.8465	25	-1.4389	0.4879
First half	Chew numbers	OJ — J	-0.2208	6.2896	25	-0.0351	1.0000
**Dataset**	**Measure of feeding behavior**	**All individuals**	**numDF**	**denDF**	**F-value**	**p-value**	
First half	Ant. frequency	(Intercept)	1	421	146.9092	<0.001	
First half	Ant. frequency	Age	3	25	8.335606	0.000519	
First half	Ant. frequency	logVolume	1	421	103.2111	<0.001	
First half	Ant. frequency	Age:Volume	3	421	5.160799	0.001636	
**First half of the feeding sequence**	**Ant. ingestion frequency**	**contrast**	**estimate**	**SE**	**df**	**t.ratio**	**p-value**
First half	Ant. frequency	S-A — I	0.510133	2.471359	25	0.206418	0.996805
First half	Ant. frequency	S-A — OJ	-5.59754	1.772932	25	-3.15722	0.020142

First half	Ant. frequency	S-A — J	-6.03832	1.313552	25	-4.59694	0.000578
First half	Ant. frequency	I — OJ	-6.10767	2.733903	25	-2.23405	0.141606
First half	Ant. frequency	I — J	-6.54845	2.460966	25	-2.66093	0.060528
First half	Ant. frequency	OJ — J	-0.44078	1.758416	25	-0.25067	0.994326
**Dataset**	**Measure of feeding behavior**	**All individuals**	**numDF**	**denDF**	**F-value**	**p-value**	
First half	Post. frequency	(Intercept)	1	155	107.4784	<0.001	
First half	Post. frequency	Age	3	25	5.316471	0.005667	
First half	Post. frequency	logVolume	1	155	0.208861	0.648302	
First half	Post. frequency	Age:Volume	3	155	1.332993	0.265713	
**First half of the feeding sequence**	**Post. ingestion frequency**	**contrast**	**estimate**	**SE**	**df**	**t.ratio**	**p-value**
First half	Post. frequency	S-A — I	-3.37411	1.338932	25	-2.52	0.081054
First half	Post. frequency	S-A — OJ	-1.24398	1.106273	25	-1.12448	0.678199
First half	Post. frequency	S-A — J	-2.43859	0.767306	25	-3.17811	0.019191
First half	Post. frequency	I — OJ	2.130133	1.526776	25	1.395184	0.513902
First half	Post. frequency	I — J	0.935527	1.302292	25	0.71837	0.88885
First half	Post. frequency	OJ — J	-1.19461	1.061632	25	-1.12525	0.677735
**Dataset**	**Measure of feeding behavior**	**All individuals**	**numDF**	**denDF**	**F-value**	**p-value**	
First half	Behavioral pattern	(Intercept)	1	297	263.6439	0	
First half	Behav. pattern	Age	3	25	4.3133	0.0139	
First half	Behav. pattern	logVolume	1	297	35.7502	0.0000	
First half	Behav. pattern	Age:Volume	3	297	0.7048	0.5498	
**First half of the feeding sequence**	**Duration**	**contrast**	**estimate**	**SE**	**df**	**t.ratio**	**p-value**
First half	Behav. pattern	S-A — I	-0.5534	1.1370	25	-0.4867	0.9613
First half	Behav. pattern	S-A — OJ	-1.0536	0.7803	25	-1.3504	0.5409

First half	Behav. pattern	S-A — J	-2.2259	0.5901	25	-3.7720	0.0046
First half	Behav. pattern	I — OJ	-0.5002	1.2302	25	-0.4066	0.9768
First half	Behav. pattern	I — J	-1.6725	1.1193	25	-1.4942	0.4558
First half	Behav. pattern	OJ — J	-1.1723	0.7541	25	-1.5545	0.4217
**Dataset**	**Measure of feeding behavior**	**All individuals**	**numDF**	**denDF**	**F-value**	**p-value**	
Last half	Duration	(Intercept)	1	455	1645.8007	0.000	
Last half	Duration	Age	3	25	3.4898	0.0305	
Last half	Duration	logVolume	1	455	151.1345	0.000	
Last half	Duration	Age:Volume	3	455	3.2055	0.023	
**Last half of the feeding sequence**	**Duration**	**contrast**	**estimate**	**SE**	**df**	**t.ratio**	**p-value**
Last half	Duration	S-A — I	-0.3338	0.2869	25	-1.1636	0.6547
Last half	Duration	S-A — OJ	-0.1914	0.1950	25	-0.9820	0.7609
Last half	Duration	S-A — J	-0.3711	0.1464	25	-2.5349	0.0786
Last half	Duration	I — OJ	0.1424	0.3145	25	0.4527	0.9685
Last half	Duration	I — J	-0.0373	0.2869	25	-0.1301	0.9992
Last half	Duration	OJ — J	-0.1797	0.1950	25	-0.9215	0.7936
**Dataset**	**Measure of feeding behavior**	**All individuals**	**numDF**	**denDF**	**F-value**	**p-value**	
Last half	Chew frequency	(Intercept)	1	432	175.5819	0	
Last half	Chew frequency	Age	3	25	7.7503	0.000798	
Last half	Chew frequency	logVolume	1	432	160.0587	0	
Last half	Chew frequency	Age:Volume	3	432	8.144118	2.76E-05	
**Last half of the feeding sequence**	**Chew frequency**	**contrast**	**estimate**	**SE**	**df**	**t.ratio**	**p-value**
Last half	Chew frequency	S-A — I	-1.74857	2.101919	25	-0.83189	0.838819

Last half	Chew frequency	S-A — OJ	-5.82001	1.53302	25	-3.79643	0.004343
Last half	Chew frequency	S-A — J	-4.86307	1.12753	25	-4.31303	0.001189
Last half	Chew frequency	I — OJ	-4.07144	2.336763	25	-1.74234	0.32397
Last half	Chew frequency	I — J	-3.1145	2.093235	25	-1.48789	0.459417
Last half	Chew frequency	OJ — J	0.956935	1.521093	25	0.629111	0.9217
**Dataset**	**Measure of feeding behavior**	**All individuals**	**numDF**	**denDF**	**F-value**	**p-value**	
Last half	Chew numbers	(Intercept)	1	455	375.93862<br>4	0.000	
Last half	Chew numbers	Age	3	25	4.569076	0.0110	
Last half	Chew numbers	logVolume	1	455	167.24148<br>9	0.000	
Last half	Chew numbers	Age:Volume	3	455	7.845748	4.09E-05	
**Last half of the feeding sequence**	**Chew numbers**	**contrast**	**estimate**	**SE**	**df**	**t.ratio**	**p-value**
Last half	Chew numbers	S-A — I	-1.9342	9.3262	25	-0.2074	0.9968
Last half	Chew numbers	S-A — OJ	-11.7222	6.3372	25	-1.8497	0.2748
Last half	Chew numbers	S-A — J	-14.9968	4.7593	25	-3.1510	0.0204
Last half	Chew numbers	I — OJ	-9.7880	10.2226	25	-0.9575	0.7743
Last half	Chew numbers	I — J	-13.0626	9.3270	25	-1.4005	0.5107
Last half	Chew numbers	OJ — J	-3.2746	6.3384	25	-0.5166	0.9543
**Dataset**	**Measure of feeding behavior**	**All individuals**	**numDF**	**denDF**	**F-value**	**p-value**	
Last half	Ant. frequency	Model failed to converge					
**Dataset**	**Measure of feeding behavior**	**All individuals**	**numDF**	**denDF**	**F-value**	**p-value**	
Last half	Post. frequency	(Intercept)	1	151	130.2603	0	

Last half	Post. frequency	Age	3	25	2.745933	0.064142	
Last half	Post. frequency	logVolume	1	151	1.649218	0.201032	
Last half	Post. frequency	Age:Volume	3	151	1.753391	0.158561	
**Last half of the feeding sequence**	**Post. ingestion frequency**	**contrast**	**estimate**	**SE**	**df**	**t.ratio**	**p-value**
Last half	Post. frequency	S-A — I	-2.10745	1.140451	25	-1.84791	0.27559
Last half	Post. frequency	S-A — OJ	-1.44056	0.917254	25	-1.57052	0.412845
Last half	Post. frequency	S-A — J	-1.51986	0.640962	25	-2.37121	0.109026
Last half	Post. frequency	I — OJ	0.666882	1.290839	25	0.516627	0.954327
Last half	Post. frequency	I — J	0.587589	1.11164	25	0.528579	0.951331
Last half	Post. frequency	OJ — J	-0.07929	0.881175	25	-0.08999	0.999731
**Dataset**	**Measure of feeding behavior**	**All individuals**	**numDF**	**denDF**	**F-value**	**p-value**	
Last half	Behavioral pattern	(Intercept)	1	313	307.9000	0	
Last half	Behav. pattern	Age	3	25	4.2339	0.0150	
Last half	Behav. pattern	logVolume	1	313	34.4132	1.13E-08	
Last half	Behav. pattern	Age:Volume	3	313	0.4035	0.7506	
**Last half of the feeding sequence**	**Behavioral pattern**	**contrast**	**estimate**	**SE**	**df**	**t.ratio**	**p-value**
Last half	Behav. pattern	S-A — I	-1.1310	1.0295	25	-1.0986	0.6937
Last half	Behav. pattern	S-A — OJ	-0.2142	0.6989	25	-0.3065	0.9898
Last half	Behav. pattern	S-A — J	-1.7523	0.5264	25	-3.3291	0.0135
Last half	Behav. pattern	I — OJ	0.9168	1.1146	25	0.8225	0.8433
Last half	Behav. pattern	I — J	-0.6214	1.0154	25	-0.6120	0.9273
Last half	Behav. pattern	OJ — J	-1.5382	0.6779	25	-2.2689	0.1326

Contrast key: S-A = Subadults-adults; OJ = Older Juveniles; J = juveniles; I = infants

SOM Table 12. Results LME models testing differences in food volume and measures of feeding behavior in of 10,000 randomly sampled feeding sequences. (H5 - volume).

Dataset	Measure of feeding behavior	Contrasts	Percentage of significant tests
Random	Duration	(Intercept)	100.00%
Random	Duration	Age	6.89%
Random	Duration	Age:logFoodvolume	16.13%
Random	Duration	logFoodvolume	100.00%
Random	Duration	S-A — I	0.00%
Random	Duration	S-A — OJ	5.12%
Random	Duration	S-A — J	9.13%
Random	Duration	I — OJ	0.01%
Random	Duration	I — J	0.00%
Random	Duration	OJ — J	0.00%
Random	Behavioral Frequency: Anterior ingestion	(Intercept)	100.00%
Random	Behavioral Frequency: Anterior ingestion	Age	86.01%
Random	Behavioral Frequency: Anterior ingestion	Age:logFoodvolume	30.08%
Random	Behavioral Frequency: Anterior ingestion	logFoodvolume	100.00%
Random	Behavioral Frequency: Anterior ingestion	S-A — I	0.00%
Random	Behavioral Frequency: Anterior ingestion	S-A — OJ	7.22%
Random	Behavioral Frequency: Anterior ingestion	S-A — J	83.16%
Random	Behavioral Frequency: Anterior ingestion	I — OJ	0.11%
Random	Behavioral Frequency: Anterior ingestion	I — J	0.46%
Random	Behavioral Frequency: Anterior ingestion	OJ — J	0.00%
Random	Behavioral Frequency: Posterior ingestion	(Intercept)	100.00%

Random	Behavioral Frequency: Posterior ingestion	Age	74.61%
Random	Behavioral Frequency: Posterior ingestion	Age:logFoodvolume	23.08%
Random	Behavioral Frequency: Posterior ingestion	logFoodvolume	18.31%
Random	Behavioral Frequency: Posterior ingestion	S-A — I	3.08%
Random	Behavioral Frequency: Posterior ingestion	S-A — OJ	0.33%
Random	Behavioral Frequency: Posterior ingestion	S-A — J	49.15%
Random	Behavioral Frequency: Posterior ingestion	I — OJ	0.02%
Random	Behavioral Frequency: Posterior ingestion	I — J	0.00%
Random	Behavioral Frequency: Posterior ingestion	OJ — J	0.08%
Random	Behavioral Frequency: Chew	(Intercept)	100.00%
Random	Behavioral Frequency: Chew	Age	63.80%
Random	Behavioral Frequency: Chew	Age:logVolume	49.14%
Random	Behavioral Frequency: Chew	logVolume	100.00%
Random	Behavioral Frequency: Chew	S-A — I	0.00%
Random	Behavioral Frequency: Chew	S-A — OJ	42.64%
Random	Behavioral Frequency: Chew	S-A — J	75.35%
Random	Behavioral Frequency: Chew	I — OJ	0.00%
Random	Behavioral Frequency: Chew	I — J	0.00%
Random	Behavioral Frequency: Chew	OJ — J	0.05%
Random	Chew Number	(Intercept)	100.00%
Random	Chew Number	Age	1.67%
Random	Chew Number	Age:logFoodvolume	48.32%
Random	Chew Number	logFoodvolume	100.00%
Random	Chew Number	S-A — I	0.00%
Random	Chew Number	S-A — OJ	6.27%

Random	Chew Number	S-A — J	1.96%
Random	Chew Number	I — OJ	0.03%
Random	Chew Number	I — J	0.00%
Random	Chew Number	OJ — J	0.12%
Random	Unique Behavioral pattern	(Intercept)	100.00%
Random	Unique Behavioral pattern	Age	99.92%
Random	Unique Behavioral pattern	Age:logVolume	0.00%
Random	Unique Behavioral pattern	logVolume	0.03%
Random	Unique Behavioral pattern	S-A — I	7.21%
Random	Unique Behavioral pattern	S-A — OJ	0.00%
Random	Unique Behavioral pattern	S-A — J	99.85%
Random	Unique Behavioral pattern	I — OJ	0.63%
Random	Unique Behavioral pattern	I — J	0.00%
Random	Unique Behavioral pattern	OJ — J	53.79%

Contrast key: S-A = Subadults-adults; OJ = Older Juveniles; J = juveniles; I = infants

SOM Table 13. Results of LME models testing differences in each measure of feeding behavior varies with food toughness and elastic modulus (FMPs; H4).

**Toughness-complete dataset**							
**Dataset**	**Measure of feeding behavior**	**All individuals**	**numDF**	**denDF**	**F-value**	**p-value**	
Complete	Duration	(Intercept)	1	455	1729.5142	<.0001	
Complete	Duration	Age	3	25	3.5788	0.0280	
Complete	Duration	logToughness	1	455	34.4314	<.0001	
Complete	Duration	Age:Toughness	3	455	1.7529	0.1554	
**Complete feeding sequence**	**Duration**	**contrast**	**estimate**	**SE**	**df**	**t.ratio**	**p-value**
Complete	Duration	S-A — I	-0.868	0.340	25	-2.554	0.0756
Complete	Duration	S-A — OJ	-0.281	0.220	25	-1.277	0.5855
Complete	Duration	S-A — J	-0.504	0.165	25	-3.048	0.0259
Complete	Duration	I — OJ	0.587	0.370	25	1.588	0.4031
Complete	Duration	I — J	0.365	0.340	25	1.073	0.7090
Complete	Duration	OJ — J	-0.223	0.220	25	-1.011	0.7443
**Dataset**	**Measure of feeding behavior**	**All individuals**	**numDF**	**denDF**	**F-value**	**p-value**	
Complete	Chew frequency	Model failed to converge					
**Dataset**	**Measure of feeding behavior**	**All individuals**	**numDF**	**denDF**	**F-value**	**p-value**	
Complete	Chew numbers	(Intercept)	1	455	163.27603	<.0001	
Complete	Chew numbers	Age	3	25	3.06376	0.0465	
Complete	Chew numbers	logToughness	1	455	0.06943	0.7923	
Complete	Chew numbers	Age:Toughness	3	455	1.11663	0.3419	
**Complete feeding sequence**	**Chew numbers**	**contrast**	**estimate**	**SE**	**df**	**t.ratio**	**p-value**
Complete	Chew numbers	S-A — I	-20.76	21.4	25	-0.971	0.7668

Complete	Chew numbers	S-A — OJ	-24.76	14.6	25	-1.700	0.3448
Complete	Chew numbers	S-A — J	-31.67	10.8	25	-2.938	0.0332
Complete	Chew numbers	I — OJ	-4.00	23.5	25	-0.170	0.9982
Complete	Chew numbers	I — J	-10.91	21.3	25	-0.511	0.9557
Complete	Chew numbers	OJ — J	-6.91	14.5	25	-0.476	0.9637
**Dataset**	**Measure of feeding behavior**	**All individuals**	**numDF**	**denDF**	**F-value**	**p-value**	
Complete	Ant. frequency	Model failed to converge					
**Dataset**	**Measure of feeding behavior**	**All individuals**	**numDF**	**denDF**	**F-value**	**p-value**	
Complete	Post. frequency	Model failed to converge					
**Dataset**	**Measure of feeding behavior**	**All individuals**	**numDF**	**denDF**	**F-value**	**p-value**	
Complete	Behavioral pattern	(Intercept)	1	360	331.2200	<.0001	
Complete	Behav. pattern	Age	3	25	5.8804	0.0035	
Complete	Behav. pattern	logToughness	1	360	48.1785	<.0001	
Complete	Behav. pattern	Age:Toughness	3	360	1.1314	0.3363	
**Complete feeding sequence**	**Behavioral Pattern**	**contrast**	**estimate**	**SE**	**df**	**t.ratio**	**p-value**
Complete	Behav. pattern	S-A — I	-4.199	1.562	25	-2.688	0.0571
Complete	Behav. pattern	S-A — OJ	-0.847	1.041	25	-0.814	0.8474
Complete	Behav. pattern	S-A — J	-3.231	0.773	25	-4.180	0.0017
Complete	Behav. pattern	I — OJ	3.352	1.699	25	1.973	0.2250
Complete	Behav. pattern	I — J	0.967	1.549	25	0.624	0.9233
Complete	Behav. pattern	OJ — J	-2.384	1.022	25	-2.332	0.1176
**Elastic modulus-complete dataset**							
**Dataset**	**Measure of**	**All individuals**	**numDF**	**denDF**	**F-value**	**p-value**	

	feeding behavior						
Complete	Duration	(Intercept)	1	455	1770.1085	<.0001	
Complete	Duration	Age	3	25	3.6125	0.0271	
Complete	Duration	logElastic	1	455	1.4612	0.2274	
Complete	Duration	Age:Elastic	3	455	0.7301	0.5344	
**Complete feeding sequence**	**Duration**	**contrast**	**estimate**	**SE**	**df**	**t.ratio**	**p-value**
Complete	Duration	S-A — I	-0.5266	0.329	25	-1.601	0.3963
Complete	Duration	S-A — OJ	-0.2942	0.217	25	-1.359	0.5358
Complete	Duration	S-A — J	-0.5129	0.163	25	-3.141	0.0209
Complete	Duration	I — OJ	0.2324	0.359	25	0.648	0.9152
Complete	Duration	I — J	0.0137	0.329	25	0.042	1.0000
Complete	Duration	OJ — J	-0.2187	0.217	25	-1.008	0.7460
**Dataset**	**Measure of feeding behavior**	**All individuals**	**numDF**	**denDF**	**F-value**	**p-value**	
Complete	Chew frequency	(Intercept)	1	435	146.5071	<0.001	
Complete	Chew frequency	Age	3	25	6.304431	0.002468	
Complete	Chew frequency	logElastic	1	435	0.96246	0.327113	
Complete	Chew frequency	Age:Elastic	3	435	2.826872	0.03828	
**Complete feeding sequence**	**Chew frequency**	**contrast**	**estimate**	**SE**	**df**	**t.ratio**	**p-value**
Complete	Chew frequency	S-A — I	-3.82665	2.741531	25	-1.39581	0.51353
Complete	Chew frequency	S-A — OJ	-4.14416	1.709459	25	-2.42425	0.098235
Complete	Chew frequency	S-A — J	-5.51573	1.272287	25	-4.33529	0.001124
Complete	Chew frequency	I — OJ	-0.31751	2.967623	25	-0.10699	0.999549
Complete	Chew frequency	I — J	-1.68908	2.739206	25	-0.61663	0.925808
Complete	Chew frequency	OJ — J	-1.37157	1.705727	25	-0.80409	0.851864
**Dataset**	**Measure of**	**All individuals**	**numDF**	**denDF**	**F-value**	**p-value**	

	**feeding behavior**						
Complete	Chew numbers	(Intercept)	1	455	164.87680	<.0001	
Complete	Chew numbers	Age	3	25	3.09585	0.045	
Complete	Chew numbers	logElastic	1	455	0.95494	0.329	
Complete	Chew numbers	Age:Elastic	3	455	3.08724	0.027	
**Complete feeding sequence**	**Chew numbers**	**contrast**	**estimate**	**SE**	**df**	**t.ratio**	**p-value**
Complete	Chew numbers	S-A — I	-14.11	20.9	25	-0.676	0.9052
Complete	Chew numbers	S-A — OJ	-25.49	14.5	25	-1.758	0.3164
Complete	Chew numbers	S-A — J	-31.75	10.7	25	-2.960	0.0316
Complete	Chew numbers	I — OJ	-11.38	23.0	25	-0.495	0.9596
Complete	Chew numbers	I — J	-17.64	20.8	25	-0.846	0.8318
Complete	Chew numbers	OJ — J	-6.26	14.4	25	-0.433	0.9722
**Dataset**	**Measure of feeding behavior**	**All individuals**	**numDF**	**denDF**	**F-value**	**p-value**	
Complete	Ant. frequency	(Intercept)	1	450	48.87247	<.0001	
Complete	Ant. frequency	Age	3	25	6.40048	0.0023	
Complete	Ant. frequency	logElastic	1	450	0.00008	0.9929	
Complete	Ant. frequency	Age:Elastic	3	450	0.46227	0.7088	
**Complete feeding sequence**	**Ant. ingestion frequency**	**contrast**	**estimate**	**SE**	**df**	**t.ratio**	**p-value**
Complete	Ant. frequency	S-A — I	-0.05295	3.178279	25	-0.01666	0.999998
Complete	Ant. frequency	S-A — OJ	-4.41789	2.02949	25	-2.17685	0.15735
Complete	Ant. frequency	S-A — J	-6.40843	1.51108	25	-4.24096	0.001427
Complete	Ant. frequency	I — OJ	-4.36494	3.450657	25	-1.26496	0.592855
Complete	Ant. frequency	I — J	-6.35548	3.173573	25	-2.00262	0.213891
Complete	Ant. frequency	OJ — J	-1.99054	2.022111	25	-0.98439	0.759514
**Dataset**	**Measure of**	**All individuals**	**numDF**	**denDF**	**F-value**	**p-value**	

	feeding behavior						
Complete	Post. frequency	(Intercept)	1	450	116.8369	0	
Complete	Post. frequency	Age	3	25	6.400481	0.002283	
Complete	Post. frequency	logElastic	1	450	7.88E-05	0.99292	
Complete	Post. frequency	Age:Elastic	3	450	0.462272	0.708768	
**Complete feeding sequence**	**Post. ingestion frequency**	**contrast**	**estimate**	**SE**	**df**	**t.ratio**	**p-value**
Complete	Post. frequency	S-A — I	-0.05295	3.178279	25	-0.01666	0.999998
Complete	Post. frequency	S-A — OJ	-4.41789	2.02949	25	-2.17685	0.15735
Complete	Post. frequency	S-A — J	-6.40843	1.51108	25	-4.24096	0.001427
Complete	Post. frequency	I — OJ	-4.36494	3.450657	25	-1.26496	0.592855
Complete	Post. frequency	I — J	-6.35548	3.173573	25	-2.00262	0.213891
Complete	Post. frequency	OJ — J	-1.99054	2.022111	25	-0.98439	0.759514
**Dataset**	**Measure of feeding behavior**	**All individuals**	**numDF**	**denDF**	**F-value**	**p-value**	
Complete	Behavioral pattern	(Intercept)	1	360	315.02355	<.0001	
Complete	Behav. pattern	Age	3	25	5.56920	0.0046	
Complete	Behav. pattern	logElastic	1	360	0.25805	0.6118	
Complete	Behav. pattern	Age:Elastic	3	360	1.50561	0.2127	
**Complete feeding sequence**	**Behavioral pattern**	**contrast**	**estimate**	**SE**	**df**	**t.ratio**	**p-value**
Complete	Behav. pattern	S-A — I	-2.9660	1.536	25	-1.932	0.2408
Complete	Behav. pattern	S-A — OJ	-0.7434	1.065	25	-0.698	0.8969
Complete	Behav. pattern	S-A — J	-2.9817	0.792	25	-3.767	0.0047
Complete	Behav. pattern	I — OJ	2.2226	1.681	25	1.322	0.5580
Complete	Behav. pattern	I — J	-0.0157	1.523	25	-0.010	1.0000
Complete	Behav. pattern	OJ — J	-2.2383	1.046	25	-2.139	0.1685

Toughness-first half dataset							
**Dataset**	**Measure of feeding behavior**	**All individuals**	**numDF**	**denDF**	**F-value**	**p-value**	
First half	Duration	(Intercept)	1	448	143.35219	0	
First half	Duration	Age	3	25	6.91476	0.0015	
First half	Duration	logToughness	1	448	69.87017	8.88e-16	
First half	Duration	Age:Toughness	3	448	3.42971	0.0171	
**First half of feeding sequence**	**Duration**	**contrast**	**estimate**	**SE**	**df**	**t.ratio**	**p-value**
First half	Duration	S-A — I	-1.6397	0.5888	25	-2.7849	0.0464
First half	Duration	S-A — OJ	-1.1654	0.3947	25	-2.9529	0.0321
First half	Duration	S-A — J	-1.1838	0.2923	25	-4.0496	0.0023
First half	Duration	I — OJ	0.4743	0.6452	25	0.7352	0.8820
First half	Duration	I — J	0.4559	0.5882	25	0.7751	0.8650
First half	Duration	OJ — J	-0.0184	0.3938	25	-0.0468	1.0000
**Dataset**	**Measure of feeding behavior**	**All individuals**	**numDF**	**denDF**	**F-value**	**p-value**	
First half	Chew frequency	Models failed to converge					
**Dataset**	**Measure of feeding behavior**	**All individuals**	**numDF**	**denDF**	**F-value**	**p-value**	
First half	Chew numbers	(Intercept)	1	448	72.361368	2.22e-16	
First half	Chew numbers	Age	3	25	2.546701	0.0787	
First half	Chew numbers	logToughness	1	448	0.363147	0.5471	
First half	Chew numbers	Age:Toughness	3	448	0.779231	0.5060	
**First half of feeding sequence**	**Chew numbers**	**contrast**	**estimate**	**SE**	**df**	**t.ratio**	**p-value**
First half	Chew numbers	S-A — I	-5.8591	10.5831	25	-0.5536	0.9447

First half	Chew numbers	S-A — OJ	-13.5074	7.2958	25	-1.8514	0.2741
First half	Chew numbers	S-A — J	-13.6977	5.3650	25	-2.5531	0.0757
First half	Chew numbers	I — OJ	-7.6483	11.6626	25	-0.6558	0.9125
First half	Chew numbers	I — J	-7.8386	10.5627	25	-0.7421	0.8791
First half	Chew numbers	OJ — J	-0.1903	7.2662	25	-0.0262	1.0000
**Dataset**	**Measure of feeding behavior**	**All individuals**	**numDF**	**denDF**	**F-value**	**p-value**	
First half	Ant. frequency	Models failed to converge					
**Dataset**	**Measure of feeding behavior**	**All individuals**	**numDF**	**denDF**	**F-value**	**p-value**	
First half	Post. frequency	Models failed to converge					
**Dataset**	**Measure of feeding behavior**	**All individuals**	**numDF**	**denDF**	**F-value**	**p-value**	
First half	Behavioral pattern	(Intercept)	1	297	245.52006	0	
First half	Behav. pattern	Age	3	25	4.00774	0.0185	
First half	Behav. pattern	logToughness	1	297	4.64990	0.0319	
First half	Behav. pattern	Age:Toughness	3	297	0.33008	0.8036	
**First half of feeding sequence**	**Post. ingestion frequency**	**contrast**	**estimate**	**SE**	**df**	**t.ratio**	**p-value**
First half	Behav. pattern	S-A — I	-1.5184	1.2165	25	-1.2482	0.6031
First half	Behav. pattern	S-A — OJ	-0.9325	0.8085	25	-1.1534	0.6608
First half	Behav. pattern	S-A — J	-2.1815	0.6129	25	-3.5590	0.0078
First half	Behav. pattern	I — OJ	0.5858	1.3088	25	0.4476	0.9695
First half	Behav. pattern	I — J	-0.6631	1.1979	25	-0.5536	0.9447
First half	Behav. pattern	OJ — J	-1.2489	0.7802	25	-1.6008	0.3964

Elastic modulus-First half dataset							
**Dataset**	**Measure of feeding behavior**	**All individuals**	**numDF**	**denDF**	**F-value**	**p-value**	
First half	Duration	(Intercept)	1	448	148.49516	0.000	
First half	Duration	Age	3	25	7.15330	0.0013	
First half	Duration	logElastic	1	448	0.55062	0.4585	
First half	Duration	Age:Elastic	3	448	1.35527	0.2559	
**First half of feeding sequence**	**Duration**	**contrast**	**estimate**	**SE**	**df**	**t.ratio**	**p-value**
First half	Duration	S-A — I	-1.1324	0.5712	25	-1.9823	0.2214
First half	Duration	S-A — OJ	-1.2258	0.3841	25	-3.1917	0.0186
First half	Duration	S-A — J	-1.2153	0.2861	25	-4.2471	0.0014
First half	Duration	I — OJ	-0.0935	0.6260	25	-0.1493	0.9988
First half	Duration	I — J	-0.0829	0.5711	25	-0.1452	0.9989
First half	Duration	OJ — J	0.0106	0.3839	25	0.0275	1.0000
**Dataset**	**Measure of feeding behavior**	**All individuals**	**numDF**	**denDF**	**F-value**	**p-value**	
First half	Chew frequency	Model failed to converge					
**Dataset**	**Measure of feeding behavior**	**All individuals**	**numDF**	**denDF**	**F-value**	**p-value**	
First half	Chew numbers	(Intercept)	1	448	72.502765	2.22E-16	
First half	Chew numbers	Age	3	25	2.563421	0.0774	
First half	Chew numbers	logElastic	1	448	4.589744	0.0327	
First half	Chew numbers	Age:Elastic	3	448	6.899142	0.0002	
**First half of feeding sequence**	**Chew numbers**	**contrast**	**estimate**	**SE**	**df**	**t.ratio**	**p-value**
First half	Chew numbers	S-A — I	-6.9426	10.3818	25	-0.6687	0.9079

First half	Chew numbers	S-A — OJ	-14.3621	7.3057	25	-1.9659	0.2275
First half	Chew numbers	S-A — J	-13.8134	5.3649	25	-2.5748	0.0724
First half	Chew numbers	I — OJ	-7.4196	11.4852	25	-0.6460	0.9159
First half	Chew numbers	I — J	-6.8708	10.3595	25	-0.6632	0.9099
First half	Chew numbers	OJ — J	0.5487	7.2739	25	0.0754	0.9998
**Dataset**	**Measure of feeding behavior**	**All individuals**	**numDF**	**denDF**	**F-value**	**p-value**	
First half	Ant. frequency	Model failed to converge					
**Dataset**	**Measure of feeding behavior**	**All individuals**	**numDF**	**denDF**	**F-value**	**p-value**	
First half	Post. frequency	Model failed to converge					
**Dataset**	**Measure of feeding behavior**	**All individuals**	**numDF**	**denDF**	**F-value**	**p-value**	
First half	Behavioral pattern	(Intercept)	1	297	246.08395	0	
First half	Behav. pattern	Age	3	25	4.01602	0.0184	
First half	Behav. pattern	logElastic	1	297	2.79190	0.0958	
First half	Behav. pattern	Age:Elastic	3	297	0.94974	0.4169	
**First half of feeding sequence**	**Behavioral pattern**	**contrast**	**estimate**	**SE**	**df**	**t.ratio**	**p-value**
First half	Behav. pattern	S-A — I	-0.8673	1.1560	25	-0.7503	0.8757
First half	Behav. pattern	S-A — OJ	-0.9088	0.8058	25	-1.1277	0.6763
First half	Behav. pattern	S-A — J	-2.1196	0.6103	25	-3.4728	0.0096
First half	Behav. pattern	I — OJ	-0.0414	1.2534	25	-0.0330	1.0000
First half	Behav. pattern	I — J	-1.2522	1.1376	25	-1.1008	0.6923
First half	Behav. pattern	OJ — J	-1.2108	0.7792	25	-1.5539	0.4220

Toughness-Last half dataset							
**Dataset**	**Measure of feeding behavior**	**All individuals**	**numDF**	**denDF**	**F-value**	**p-value**	
Last half	Duration	(Intercept)	1	455	1588.7774	0.000	
Last half	Duration	Age	3	25	3.2606	0.0382	
Last half	Duration	logToughness	1	455	18.9225	1.68E-05	
Last half	Duration	Age:Toughness	3	455	2.0721	0.1031	
**Last half of feeding sequence**	**Duration**	**contrast**	**estimate**	**SE**	**df**	**t.ratio**	**p-value**
Last half	Duration	S-A — I	-0.8334	0.3129	25	-2.6631	0.0603
Last half	Duration	S-A — OJ	-0.2224	0.1963	25	-1.1329	0.6731
Last half	Duration	S-A — J	-0.4158	0.1487	25	-2.7960	0.0453
Last half	Duration	I — OJ	0.6110	0.3385	25	1.8052	0.2946
Last half	Duration	I — J	0.4176	0.3132	25	1.3332	0.5513
Last half	Duration	OJ — J	-0.1934	0.1968	25	-0.9825	0.7606
**Dataset**	**Measure of feeding behavior**	**All individuals**	**numDF**	**denDF**	**F-value**	**p-value**	
Last half	Chew frequency	Model failed to converge					
**Dataset**	**Measure of feeding behavior**	**All individuals**	**numDF**	**denDF**	**F-value**	**p-value**	
Last half	Chew numbers	(Intercept)	1	455	279.874759	0.000	
Last half	Chew numbers	Age	3	25	3.380038	0.0339	
Last half	Chew numbers	logToughness	1	455	0.529801	0.4671	
Last half	Chew numbers	Age:Toughness	3	455	1.363659	0.2533	
**Last half of feeding sequence**	**Chew numbers**	**contrast**	**estimate**	**SE**	**df**	**t.ratio**	**p-value**
Last half	Chew numbers	S-A — I	-14.8492	11.3499	25	-1.3083	0.5664

Last half	Chew numbers	S-A — OJ	-12.9253	7.3290	25	-1.7636	0.3138
Last half	Chew numbers	S-A — J	-17.0574	5.5076	25	-3.0971	0.0231
Last half	Chew numbers	I — OJ	1.9239	12.3393	25	0.1559	0.9986
Last half	Chew numbers	I — J	-2.2082	11.3525	25	-0.1945	0.9973
Last half	Chew numbers	OJ — J	-4.1321	7.3329	25	-0.5635	0.9419
**Dataset**	**Measure of feeding behavior**	**All individuals**	**numDF**	**denDF**	**F-value**	**p-value**	
Last half	Ant. frequency	Model failed to converge					
**Dataset**	**Measure of feeding behavior**	**All individuals**	**numDF**	**denDF**	**F-value**	**p-value**	
Last half	Post. frequency	Model failed to converge					
**Dataset**	**Measure of feeding behavior**	**All individuals**	**numDF**	**denDF**	**F-value**	**p-value**	
Last half	Behavioral pattern	(Intercept)	1	313	309.7162	0	
Last half	Behav. pattern	Age	3	25	4.2570	0.0147	
Last half	Behav. pattern	logToughness	1	313	12.4637	0.0005	
Last half	Behav. pattern	Age:Toughness	3	313	1.4476	0.2289	
**Last half of feeding sequence**	**Behavioral pattern**	**contrast**	**estimate**	**SE**	**df**	**t.ratio**	**p-value**
Last half	Behav. pattern	S-A — I	-2.1030	1.0542	25	-1.9949	0.2167
Last half	Behav. pattern	S-A — OJ	-0.1491	0.6965	25	-0.2141	0.9964
Last half	Behav. pattern	S-A — J	-1.7734	0.5271	25	-3.3644	0.0124
Last half	Behav. pattern	I — OJ	1.9539	1.1348	25	1.7218	0.3339
Last half	Behav. pattern	I — J	0.3297	1.0394	25	0.3172	0.9887
Last half	Behav. pattern	OJ — J	-1.6242	0.6739	25	-2.4102	0.1010

Elastic modulus-Last half dataset							
**Dataset**	**Measure of feeding behavior**	**All individuals**	**numDF**	**denDF**	**F-value**	**p-value**	
Last half	Duration	(Intercept)	1	455	1619.5219	0.000	
Last half	Duration	Age	3	25	3.2879	0.0372	
Last half	Duration	logElastic	1	455	0.7426	0.3893	
Last half	Duration	Age:Elastic	3	455	0.8982	0.4420	
**Last half of feeding sequence**	**Duration**	**contrast**	**estimate**	**SE**	**df**	**t.ratio**	**p-value**
Last half	Duration	S-A — I	-0.5166	0.3008	25	-1.7175	0.3361
Last half	Duration	S-A — OJ	-0.2310	0.1938	25	-1.1922	0.6372
Last half	Duration	S-A — J	-0.4231	0.1472	25	-2.8741	0.0382
Last half	Duration	I — OJ	0.2856	0.3265	25	0.8747	0.8178
Last half	Duration	I — J	0.0935	0.3012	25	0.3105	0.9894
Last half	Duration	OJ — J	-0.1921	0.1944	25	-0.9880	0.7575
**Dataset**	**Measure of feeding behavior**	**All individuals**	**numDF**	**denDF**	**F-value**	**p-value**	
Last half	Chew frequency	(Intercept)	1	432	144.3554	<0.001	
Last half	Chew frequency	Age	3	25	6.35186	0.002375	
Last half	Chew frequency	logElastic	1	432	0.807638	0.36932	
Last half	Chew frequency	Age:Elastic	3	432	2.707505	0.044861	
**Last half of feeding sequence**	**Chew frequency**	**contrast**	**estimate**	**SE**	**df**	**t.ratio**	**p-value**
Last half	Chew frequency	S-A — I	-3.80808	2.7547	25	-1.38239	0.521563
Last half	Chew frequency	S-A — OJ	-4.11915	1.718988	25	-2.39626	0.103811
Last half	Chew frequency	S-A — J	-5.5047	1.280529	25	-4.29877	0.001233
Last half	Chew frequency	I — OJ	-0.31107	2.982159	25	-0.10431	0.999582

Last half	Chew frequency	I — J	-1.69662	2.752837	25	-0.61632	0.92591
Last half	Chew frequency	OJ — J	-1.38555	1.716	25	-0.80743	0.850324
**Dataset**	**Measure of feeding behavior**	**All individuals**	**numDF**	**denDF**	**F-value**	**p-value**	
Last half	Chew numbers	(Intercept)	1	455	287.456603	0.000	
Last half	Chew numbers	Age	3	25	3.451813	0.0316	
Last half	Chew numbers	logElastic	1	455	0.018425	0.8921	
Last half	Chew numbers	Age:Elastic	3	455	1.526731	0.2068	
**Last half of feeding sequence**	**Chew numbers**	**contrast**	**estimate**	**SE**	**df**	**t.ratio**	**p-value**
Last half	Chew numbers	S-A — I	-7.1141	10.8931	25	-0.6531	0.9135
Last half	Chew numbers	S-A — OJ	-13.1953	7.2201	25	-1.8276	0.2845
Last half	Chew numbers	S-A — J	-17.0233	5.4311	25	-3.1344	0.0212
Last half	Chew numbers	I — OJ	-6.0812	11.8903	25	-0.5114	0.9556
Last half	Chew numbers	I — J	-9.9093	10.8970	25	-0.9094	0.8000
Last half	Chew numbers	OJ — J	-3.8280	7.2259	25	-0.5298	0.9510
**Dataset**	**Measure of feeding behavior**	**All individuals**	**numDF**	**denDF**	**F-value**	**p-value**	
Last half	Ant. frequency	Model failed to converge					
**Dataset**	**Measure of feeding behavior**	**All individuals**	**numDF**	**denDF**	**F-value**	**p-value**	
Last half	Post. frequency	(Intercept)	1	151	121.2715	0	
Last half	Post. frequency	Age	3	25	2.543315	0.079009	
Last half	Post. frequency	logElastic	1	151	4.654017	0.032563	
Last half	Post. frequency	Age:Elastic	3	151	2.27139	0.082579	
**Last half of feeding sequence**	**Post. ingestion frequency**	**contrast**	**estimate**	**SE**	**df**	**t.ratio**	**p-value**

Last half	Post. frequency	S-A — I	-0.19104	1.542144	25	-0.12388	0.999301
Last half	Post. frequency	S-A — OJ	-0.781	0.926169	25	-0.84326	0.833347
Last half	Post. frequency	S-A — J	-1.87278	0.696306	25	-2.6896	0.056967
Last half	Post. frequency	I — OJ	-0.58996	1.635021	25	-0.36083	0.983543
Last half	Post. frequency	I — J	-1.68175	1.516689	25	-1.10883	0.68755
Last half	Post. frequency	OJ — J	-1.09179	0.883134	25	-1.23626	0.610393
**Dataset**	**Measure of feeding behavior**	**All individuals**	**numDF**	**denDF**	**F-value**	**p-value**	
Last half	Behavioral pattern	(Intercept)	1	313	304.45757	0	
Last half	Behav. pattern	Age	3	25	4.18428	0.0157	
Last half	Behav. pattern	logElastic	1	313	0.40271	0.5262	
Last half	Behav. pattern	Age:Elastic	3	313	1.23894	0.2956	
**Last half of feeding sequence**	**Behavioral pattern**	**contrast**	**estimate**	**SE**	**df**	**t.ratio**	**p-value**
Last half	Behav. pattern	S-A — I	-1.7505	1.0143	25	-1.7258	0.3320
Last half	Behav. pattern	S-A — OJ	-0.0999	0.7006	25	-0.1426	0.9989
Last half	Behav. pattern	S-A — J	-1.6576	0.5294	25	-3.1310	0.0214
Last half	Behav. pattern	I — OJ	1.6506	1.0999	25	1.5006	0.4521
Last half	Behav. pattern	I — J	0.0929	0.9997	25	0.0929	0.9997
Last half	Behav. pattern	OJ — J	-1.5578	0.6792	25	-2.2935	0.1266

Contrast key: S-A = Subadults-adults; OJ = Older Juveniles; J = juveniles; I = infants

SOM Table 14. Results LME models testing differences in food toughness and elastic modulus and measures of feeding behavior in of 10,000 randomly sampled feeding sequences (H4 - FMP).

Dataset	Measure of feeding behavior	Contrasts	Percentage of significant tests
Toughness			
Random	Duration	(Intercept)	100.00%
Random	Duration	Age	3.47%
Random	Duration	Age:logtoughness	8.30%
Random	Duration	logtoughness	1.06%
Random	Duration	S-A — I	0.15%
Random	Duration	S-A — OJ	0.31%
Random	Duration	S-A — J	1.55%
Random	Duration	I — OJ	0.00%
Random	Duration	I — J	0.00%
Random	Duration	OJ — J	0.00%
Random	Behavioral Frequency: Anterior ingestion	Models failed to converge	
Random	Behavioral Frequency: Posterior ingestion	Models failed to converge	
Random	Behavioral Frequency: Chew	Models failed to converge	
Random	Chew Number	(Intercept)	100.00%
Random	Chew Number	Age	0.15%
Random	Chew Number	Age:logtoughness	0.01%
Random	Chew Number	logtoughness	99.93%
Random	Chew Number	S-A — I	0.00%

Random	Chew Number	S-A — OJ	0.07%
Random	Chew Number	S-A — J	0.00%
Random	Chew Number	I — OJ	0.00%
Random	Chew Number	I — J	0.00%
Random	Chew Number	OJ — J	0.00%
Random	Unique Behavioral pattern	(Intercept)	100.00%
Random	Unique Behavioral pattern	Age	99.90%
Random	Unique Behavioral pattern	Age:logToughness	0.13%
Random	Unique Behavioral pattern	logToughness	0.40%
Random	Unique Behavioral pattern	S-A — I	12.11%
Random	Unique Behavioral pattern	S-A — OJ	0.01%
Random	Unique Behavioral pattern	S-A — J	99.88%
Random	Unique Behavioral pattern	I — OJ	2.62%
Random	Unique Behavioral pattern	I — J	0.06%
Random	Unique Behavioral pattern	OJ — J	56.68%
Elastic Modulus			
Random	Duration	(Intercept)	100.00%
Random	Duration	Age	3.46%
Random	Duration	Age:logelastic	31.42%
Random	Duration	logelastic	31.04%
Random	Duration	S-A — I	0.04%
Random	Duration	S-A — OJ	0.76%
Random	Duration	S-A — J	1.91%
Random	Duration	I — OJ	0.00%

Random	Duration	I — J	0.00%
Random	Duration	OJ — J	0.00%
Random	Behavioral Frequency: Anterior ingestion	(Intercept)	100.00%
Random	Behavioral Frequency: Anterior ingestion	Age	77.35%
Random	Behavioral Frequency: Anterior ingestion	Age:logelastic	0.00%
Random	Behavioral Frequency: Anterior ingestion	logelastic	2.51%
Random	Behavioral Frequency: Anterior ingestion	S-A — I	0.00%
Random	Behavioral Frequency: Anterior ingestion	S-A — OJ	2.30%
Random	Behavioral Frequency: Anterior ingestion	S-A — J	64.34%
Random	Behavioral Frequency: Anterior ingestion	I — OJ	0.03%
Random	Behavioral Frequency: Anterior ingestion	I — J	0.12%
Random	Behavioral Frequency: Anterior ingestion	OJ — J	0.00%
Random	Behavioral Frequency: Posterior ingestion	(Intercept)	100.00%
Random	Behavioral Frequency: Posterior ingestion	Age	74.88%
Random	Behavioral Frequency: Posterior ingestion	Age:logelastic	12.87%
Random	Behavioral Frequency: Posterior ingestion	logelastic	20.64%
Random	Behavioral Frequency: Posterior ingestion	S-A — I	27.68%
Random	Behavioral Frequency: Posterior ingestion	S-A — OJ	0.09%
Random	Behavioral Frequency: Posterior ingestion	S-A — J	50.81%
Random	Behavioral Frequency: Posterior ingestion	I — OJ	2.70%
Random	Behavioral Frequency: Posterior ingestion	I — J	0.52%
Random	Behavioral Frequency: Posterior ingestion	OJ — J	0.12%
Random	Behavioral Frequency: Chew	(Intercept)	100.00%
Random	Behavioral Frequency: Chew	Age	54.93%

Random	Behavioral Frequency: Chew	Age:logelastic	18.43%
Random	Behavioral Frequency: Chew	logelastic	7.53%
Random	Behavioral Frequency: Chew	S-A — I	0.00%
Random	Behavioral Frequency: Chew	S-A — OJ	22.02%
Random	Behavioral Frequency: Chew	S-A — J	50.09%
Random	Behavioral Frequency: Chew	I — OJ	0.00%
Random	Behavioral Frequency: Chew	I — J	0.00%
Random	Behavioral Frequency: Chew	OJ — J	0.01%
Random	Chew Number	(Intercept)	100.00%
Random	Chew Number	Age	0.20%
Random	Chew Number	Age:logelastic	55.62%
Random	Chew Number	logelastic	45.97%
Random	Chew Number	S-A — I	0.00%
Random	Chew Number	S-A — OJ	0.51%
Random	Chew Number	S-A — J	0.05%
Random	Chew Number	I — OJ	0.00%
Random	Chew Number	I — J	0.00%
Random	Chew Number	OJ — J	0.01%
Random	Unique Behavioral pattern	(Intercept)	100.00%
Random	Unique Behavioral pattern	Age	99.94%
Random	Unique Behavioral pattern	Age:logElastic	1.57%
Random	Unique Behavioral pattern	logElastic	0.00%
Random	Unique Behavioral pattern	S-A — I	11.97%
Random	Unique Behavioral pattern	S-A — OJ	0.00%

Random	Unique Behavioral pattern	S-A — J	99.89%
Random	Unique Behavioral pattern	I — OJ	1.54%
Random	Unique Behavioral pattern	I — J	0.00%
Random	Unique Behavioral pattern	OJ — J	59.28%

Contrast key: S-A = Subadults-adults; OJ = Older Juveniles; J = juveniles; I = infants

SOM Table 15. Comparison of results for each hypothesis using the continuous and discontinuous datasets (NS = not significant, S = significant). The tally column compares how many of the discontinuous results match the results from the continuous dataset. The random tests were considered "significant" if over 50% of the 10,000 samples were significant.

**H1-4**	**Explanatory variable**	**Feeding variable**	**Dataset**	**All**[1,2]	**S-A**[1,2]	**OJ**[1,2]	**J**[1,2]	**I**[1,2]			**Tally**
H1	Sex	Duration	Complete	NS	NS	NS	NS	NS			
H1	Sex	Duration	First half	NS	NS	NS	NS	NS			5/5
H1	Sex	Duration	Last half	NS	NS	NS	NS	NS			5/5
H1	Sex	Duration	Random	NS	NS	NS	NS	NS			5/5
H1	Sex	Anterior ingestion frequency	Complete	NS	NS	NS	NS	NS			
H1	Sex	Anterior ingestion frequency	First half	NS	NS	NS	NS	NS			5/5
H1	Sex	Anterior ingestion frequency	Last half	NS	NS	NS	NS	NS			5/5
H1	Sex	Anterior ingestion frequency	Random	NS	NS	NS	NS	NS			5/5
H1	Sex	Posterior ingestion frequency	Complete	NS	NS	NS	NS	NS			
H1	Sex	Posterior ingestion frequency	First half	NS	NS	NS	NS	NS			5/5
H1	Sex	Posterior ingestion frequency	Last half	NS	NS	NS	NS	NS			5/5
H1	Sex	Posterior ingestion frequency	Random	NS	NS	NS	NS	NS			5/5
H1	Sex	Chew frequency	Complete	NS	NS	NS	NS	NS			
H1	Sex	Chew frequency	First half	NS	NS	NS	NS	NS			5/5
H1	Sex	Chew frequency	Last half	NS	NS	NS	NS	NS			5/5
H1	Sex	Chew frequency	Random	NS	NS	NS	NS	NS			5/5

H1	Sex	Chew number	Complete	NS	NS	NS	NS	NS			
H1	Sex	Chew number	First half	NS	NS	NS	NS	NS			5/5
H1	Sex	Chew number	Last half	NS	NS	NS	NS	NS			5/5
H1	Sex	Chew number	Random	NS	NS	NS	NS	NS			5/5
H1	Sex	Unique behavioral pattern	Complete	NS	NS	NS	NS	NS			
H1	Sex	Unique behavioral pattern	First half	NS	NS	NS	NS	NS			5/5
H1	Sex	Unique behavioral pattern	Last half	NS	NS	NS	NS	NS			5/5
H1	Sex	Unique behavioral pattern	Random	NS	NS	NS	NS	NS			5/5
**H1-4**	**Explanatory variable**	**Feeding variable**	**Dataset**	**[1,2]All**	**S-A / OJ[1,2]**	**S-A / J[1,2]**	**S-A / I[1,2]**	**OJ / J[1,2]**	**OJ / I[1,2]**	**J / I[1,2]**	**Tally**
H2	Age	Duration	Complete	S	NS	NS	NS	S	NS	NS	
H2	Age	Duration	First half	S	S	NS	NS	S	NS	NS	6/7
H2	Age	Duration	Last half	S	NS	NS	NS	S	NS	NS	7/7
H2	Age	Duration	Random	NS	NS	NS	NS	NS	NS	NS	5/7
H2	Age	Anterior ingestion frequency	Complete	S	NS	NS	NS	S	NS	NS	
H2	Age	Anterior ingestion frequency	First half	S	NS	NS	NS	S	NS	NS	7/7
H2	Age	Anterior ingestion frequency	Last half	NS	NS	NS	NS	NS	NS	NS	5/7
H2	Age	Anterior ingestion frequency	Random	S	NS	S	NS	NS	NS	NS	5/7
H2	Age	Posterior ingestion frequency	Complete	S	NS	NS	S	S	NS	NS	
H2	Age	Posterior ingestion frequency	First half	S	NS	NS	S	S	NS	NS	7/7
H2	Age	Posterior ingestion frequency	Last half	S	NS	NS	NS	NS	NS	NS	6/7

H2	Age	Posterior ingestion frequency	Random	S	NS	NS	NS	NS	NS	NS	5/7
H2	Age	Chew frequency	Complete	S	S	NS	NS	S	NS	NS	
H2	Age	Chew frequency	First half	NS	NS	NS	NS	NS	NS	NS	5/7
H2	Age	Chew frequency	Last half	S	S	NS	NS	S	NS	NS	7/7
H2	Age	Chew frequency	Random	S	NS	NS	NS	NS	NS	NS	5/7
H2	Age	Chew number	Complete	S	S	NS	NS	S	NS	NS	
H2	Age	Chew number	First half	S	S	NS	NS	NS	NS	NS	6/7
H2	Age	Chew number	Last half	S	S	NS	NS	S	NS	NS	7/7
H2	Age	Chew number	Random	NS	NS	NS	NS	NS	NS	NS	4/7
H2	Age	Unique behavioral pattern	Complete	S	NS	S	S	S	NS	NS	
H2	Age	Unique behavioral pattern	First half	S	NS	NS	NS	S	NS	NS	6/7
H2	Age	Unique behavioral pattern	Last half	S	NS	S	NS	S	NS	NS	7/7
H2	Age	Unique behavioral pattern	Random	S	NS	S	NS	S	NS	NS	6/7
**H1-4**	**Explanatory variable**	**Feeding variable**	**Dataset**	**[1,2]All**	**S-A / OJ[1,2]**	**S-A / J[1,2]**	**S-A / I[1,2]**	**OJ / J[1,2]**	**OJ / I[1,2]**	**J / I[1,2]**	**Tally**
H3	Volume	Duration	Complete	S	NS	S	NS	NS	NS	NS	
H3	Volume	Duration	First half	S	S	S	NS	NS	NS	NS	6/7
H3	Volume	Duration	Last half	S	NS	NS	NS	NS	NS	NS	6/7
H3	Volume	Duration	Random	S	NS	NS	NS	NS	NS	NS	6/7
H3	Volume	Anterior ingestion frequency	Complete	S	S	S	NS	NS	NS	NS	
H3	Volume	Anterior ingestion frequency	First half	S	S	S	NS	NS	NS	NS	7/7

H3	Volume	Anterior ingestion frequency	Last half	-	NS	S	NS	NS	NS	NS	6/6
H3	Volume	Anterior ingestion frequency	Random	S	NS	S	NS	NS	NS	NS	6/7
H3	Volume	Posterior ingestion frequency	Complete	NS	NS	NS	NS	NS	NS	NS	
H3	Volume	Posterior ingestion frequency	First half	NS	NS	S	NS	NS	NS	NS	6/7
H3	Volume	Posterior ingestion frequency	Last half	NS	NS	NS	NS	NS	NS	NS	7/7
H3	Volume	Posterior ingestion frequency	Random	NS	NS	NS	NS	NS	NS	NS	7/7
H3	Volume	Chew frequency	Complete	S	S	S	NS	NS	NS	NS	
H3	Volume	Chew frequency	First half	S	NS	NS	NS	NS	NS	NS	5/7
H3	Volume	Chew frequency	Last half	S	S	S	NS	NS	NS	NS	7/7
H3	Volume	Chew frequency	Random	S	NS	S	NS	NS	NS	NS	6/7
H3	Volume	Chew number	Complete	S	NS	S	NS	NS	NS	NS	
H3	Volume	Chew number	First half	S	NS	S	NS	NS	NS	NS	7/7
H3	Volume	Chew number	Last half	S	NS	S	NS	NS	NS	NS	7/7
H3	Volume	Chew number	Random	S	NS	NS	NS	NS	NS	NS	6/7
H3	Volume	Unique behavioral pattern	Complete	S	NS	S	NS	NS	NS	NS	
H3	Volume	Unique behavioral pattern	First half	S	NS	S	NS	NS	NS	NS	7/7
H3	Volume	Unique behavioral pattern	Last half	S	NS	S	NS	NS	NS	NS	7/7
H3	Volume	Unique behavioral pattern	Random	NS	NS	S	NS	S	NS	NS	5/7
**H1-4**	**Explanatory variable**	**Feeding variable**	**Dataset**	**[1,2]All**	**S-A / OJ[1,2]**	**S-A / J[1,2]**	**S-A / I[1,2]**	**OJ / J[1,2]**	**OJ / I[1,2]**	**J / I[1,2]**	**Tally**
H4	Toughness	Duration	Complete	S	NS	S	NS	NS	NS	NS	

H4	Toughness	Duration	First half	S	S	S	S	NS	NS	NS	5/7
H4	Toughness	Duration	Last half	S	NS	S	NS	NS	NS	NS	7/7
H4	Toughness	Duration	Random	NS	NS	NS	NS	NS	NS	NS	5/7
H4	Toughness	Anterior ingestion frequency	Complete	-	-	-	-	-	-	-	
H4	Toughness	Anterior ingestion frequency	First half	-	-	-	-	-	-	-	-
H4	Toughness	Anterior ingestion frequency	Last half	-	-	-	-	-	-	-	-
H4	Toughness	Anterior ingestion frequency	Random	-	-	-	-	-	-	-	-
H4	Toughness	Posterior ingestion frequency	Complete	-	-	-	-	-	-	-	
H4	Toughness	Posterior ingestion frequency	First half	-	-	-	-	-	-	-	-
H4	Toughness	Posterior ingestion frequency	Last half	-	-	-	-	-	-	-	-
H4	Toughness	Posterior ingestion frequency	Random	-	-	-	-	-	-	-	-
H4	Toughness	Chew frequency	Complete	-	-	-	-	-	-	-	
H4	Toughness	Chew frequency	First half	-	-	-	-	-	-	-	-
H4	Toughness	Chew frequency	Last half	-	-	-	-	-	-	-	-
H4	Toughness	Chew frequency	Random	-	-	-	-	-	-	-	-
H4	Toughness	Chew number	Complete	S	NS	S	NS	NS	NS	NS	
H4	Toughness	Chew number	First half	NS	NS	NS	NS	NS	NS	NS	5/7
H4	Toughness	Chew number	Last half	NS	NS	S	NS	NS	NS	NS	6/7
H4	Toughness	Chew number	Random	S	NS	NS	NS	NS	NS	NS	6/7
H4	Toughness	Unique behavioral pattern	Complete	S	NS	S	NS	NS	NS	NS	

H4	Toughness	Unique behavioral pattern	First half	S	NS	S	NS	NS	NS	NS	7/7
H4	Toughness	Unique behavioral pattern	Last half	S	NS	S	NS	NS	NS	NS	7/7
H4	Toughness	Unique behavioral pattern	Random	NS	NS	S	NS	S	NS	NS	4/7
**H1-4**	**Explanatory variable**	**Feeding variable**	**Dataset**	**[1,2]All**	**S-A / OJ[1,2]**	**S-A / J[1,2]**	**S-A / I[1,2]**	**OJ / J[1,2]**	**OJ / I[1,2]**	**J / I[1,2]**	**Tally**
H4	Modulus	Duration	Complete	NS	NS	S	NS	NS	NS	NS	
H4	Modulus	Duration	First half	NS	S	S	NS	NS	NS	NS	6/7
H4	Modulus	Duration	Last half	NS	NS	S	NS	NS	NS	NS	7/7
H4	Modulus	Duration	Random	NS	NS	NS	NS	NS	NS	NS	6/7
H4	Modulus	Anterior ingestion frequency	Complete	NS	NS	S	NS	NS	NS	NS	
H4	Modulus	Anterior ingestion frequency	First half	-	-	-	-	-	-	-	-
H4	Modulus	Anterior ingestion frequency	Last half	-	-	-	-	-	-	-	-
H4	Modulus	Anterior ingestion frequency	Random	NS	NS	S	NS	NS	NS	NS	7/7
H4	Modulus	Posterior ingestion frequency	Complete	NS	NS	S	NS	NS	NS	NS	
H4	Modulus	Posterior ingestion frequency	First half	-	-	-	-	-	-	-	-
H4	Modulus	Posterior ingestion frequency	Last half	NS	NS	NS	NS	NS	NS	NS	6/7
H4	Modulus	Posterior ingestion frequency	Random	NS	NS	S	NS	NS	NS	NS	7/7
H4	Modulus	Chew frequency	Complete	NS	NS	S	NS	NS	NS	NS	
H4	Modulus	Chew frequency	First half	NS	-	-	-	-	-	NS	2/2
H4	Modulus	Chew frequency	Last half	NS	NS	S	NS	NS	NS	NS	7/7
H4	Modulus	Chew frequency	Random	NS	NS	S	NS	NS	NS	NS	7/7

H4	Modulus	Chew number	Complete	NS	NS	S	NS	NS	NS	NS	
H4	Modulus	Chew number	First half	NS	NS	NS	NS	NS	NS	NS	6/7
H4	Modulus	Chew number	Last half	NS	NS	S	NS	NS	NS	NS	7/7
H4	Modulus	Chew number	Random	NS	NS	NS	NS	NS	NS	NS	6/7
H4	Modulus	Unique behavioral pattern	Complete	NS	NS	S	NS	NS	NS	NS	
H4	Modulus	Unique behavioral pattern	First half	NS	NS	S	NS	NS	NS	NS	7/7
H4	Modulus	Unique behavioral pattern	Last half	NS	NS	S	NS	NS	NS	NS	7/7
H4	Modulus	Unique behavioral pattern	Random	NS	NS	S	NS	S	NS	NS	6/7
